# A Phase II Exploratory Study to Identify Biomarkers Predictive of Clinical Response to Regorafenib in Patients with Metastatic Colorectal Cancer Who Have Failed First-Line Therapy

**DOI:** 10.3390/ijms25010043

**Published:** 2023-12-19

**Authors:** Karen Gambaro, Maud Marques, Suzan McNamara, Mathilde Couetoux du Tertre, Cyrla Hoffert, Archana Srivastava, Anna Schab, Thierry Alcindor, Adrian Langleben, Lucas Sideris, Mahmoud Abdelsalam, Mustapha Tehfe, Felix Couture, Gerald Batist, Petr Kavan

**Affiliations:** 1 Canadian National Centres of Excellence-Exactis Innovations, Montreal, QC H3T 1Y6, Canada; kgambaro@exactis.ca (K.G.); mmarques@exactis.ca (M.M.); smcnamara@exactis.ca (S.M.);; 2 Consortium de Recherche en Oncologie Clinique du Québec (Q-CROC), Quebec, QC G1V 3X8, Canada; 3 Segal Cancer Centre-Jewish General Hospital, Montreal, QC H3T 1E2, Canada; 4 McGill University Health Centre, Montreal, QC H4A 3J1, Canada; 5 St. Mary’s Hospital, Montreal, QC H3T 0A2, Canada; 6 Hôpital Maisonneuve Rosemont, Montreal, QC H1T 2M4, Canada; 7 The Moncton Hospital, Moncton, NB E1C 6Z8, Canada; 8 Hematology-Oncology, Oncology Center-Centre Hospitalier de l’Université de Montreal, Montreal, QC H2X 0C1, Canada; 9 Hôtel-Dieu de Québec, Quebec, QC G1R 2J6, Canada

**Keywords:** metastatic colorectal cancer (mCRC), biomarkers, regorafenib, acquired resistance (ARES), intrinsic resistance (IRES), progression-free survival (PFS)

## Abstract

Single-agent regorafenib is approved in Canada for metastatic colorectal cancer (mCRC) patients who have failed previous lines of therapy. Identifying prognostic biomarkers is key to optimizing therapeutic strategies for these patients. In this clinical study (NCT01949194), we evaluated the safety and efficacy of single-agent regorafenib as a second-line therapy for mCRC patients who received it after failing first-line therapy with an oxaliplatin or irinotecan regimen with or without bevacizumab. Using various omics approaches, we also investigated putative biomarkers of response and resistance to regorafenib in metastatic lesions and blood samples in the same cohort. Overall, the safety profile of regorafenib seemed similar to the CORRECT trial, where regorafenib was administered as ≥ 2 lines of therapy. While the mutational landscape showed typical mutation rates for the top five driver genes (APC, KRAS, BRAF, PIK3CA, and TP53), KRAS mutations were enriched in intrinsically resistant lesions. Additional exploration of genomic-phenotype associations revealed several biomarker candidates linked to unfavorable prognoses in patients with mCRC using various approaches, including pathway analysis, cfDNA profiling, and copy number analysis. However, further research endeavors are necessary to validate the potential utility of these promising genes in understanding patients’ responses to regorafenib treatment.

## 1. Introduction

Colorectal cancer (CRC) is the third most common cancer in Canada and the United States and is the second most common cause of cancer mortality [1]. Approximately 25% to 50% of patients diagnosed with colorectal cancer will develop liver metastases at some point, either at the time of initial presentation or during disease recurrence [2]. While surgical resection is the preferred course of action, few patients with hepatic metastases are eligible for it, and most patients undergo a treatment approach combining chemotherapy and targeted therapy.

First-line therapy for metastatic colorectal cancer (mCRC) in Canada is a combination of chemotherapy drugs. FOLFOX includes fluorouracil (5-FU) as a 46 h infusion, folinic acid, and oxaliplatin, whereas XELOX contains the prodrug capecitabine instead of 5-FU. In the FOLFIRI combination, oxaliplatin is replaced with the topoisomerase inhibitor irinotecan, and it is administered alone or in combination with the anti-angiogenic agent bevacizumab. Anti-angiogenic agents have been incorporated into clinical practice to treat mCRC, improving PFS and median overall survival (mOS) [3]. Although first-line systemic chemotherapy improves mOS and PFS, clinical resistance is almost inevitable for advanced mCRC within 6 to 12 months of any given therapy [4]. The second-line therapy for mCRC is typically either FOLFOX or FOLFIRI in combination or not with bevacizumab or aflibercept.

The mitogen-activated protein kinase (MAPK) signaling pathway (RAS/RAF/MEK/ERK) plays an important role in mediating responses to growth signals and angiogenic factors [5]. This pathway is frequently disrupted or excessively activated in human tumors as a result of various factors such as the presence of activated RAS, mutations in the BRAF gene, or overexpression of growth factor receptors [6]. In colorectal cancer, mutant BRAF is found with a 5–12% frequency, and activated RAS is found in approximately 38% of CRC subjects [7,8]. Therefore, blocking this signaling cascade could offer clinical advantages in particular CRC cases.

Regorafenib is a multikinase inhibitor, targeting both tumor cell proliferation/survival and tumor vasculature, including vascular endothelial growth factor (VEGF). Regorafenib deactivates tumors across three dimensions: angiogenesis, oncogenesis, and stromagenesis [9]. Specifically, it inhibits angiogenic receptor tyrosine kinases (RTK), including vascular endothelial growth factor receptors (VEGFR 1-3) and endothelial-specific receptor tyrosine kinases with immunoglobulin-like loops and epidermal growth factor homology domains-2 (TIE2). It also hinders oncogenic receptors, including KIT, RET, and BRAF receptor tyrosine kinases. Also, it targets stromal RTKs like platelet-derived growth factor receptor (PDGFR) and fibroblast growth factor receptor (FGFR). Regorafenib transcends its structural relative, sorafenib, with an evolved role as an inhibitor targeting RAF1 among a wider array of kinases. This expanded inhibitory capacity allows for a broader therapeutic attack on various pathways. The core of its therapeutic impact is rooted in its capacity to obstruct angiogenesis and to sculpt the tumor microenvironment through a plethora of molecular interactions. By simultaneously inhibiting VEGF receptors and TIE2, regorafenib enforces a combined anti-angiogenic thrust while potentially orchestrating a novel form of vascular stabilization [10]. This multipronged approach extends to intercepting pathways that tumors may exploit to evade VEGF inhibitor effects, with regorafenib maintaining its anti-angiogenic stance even against VEGF-resistant tumor cells. In preclinical studies, regorafenib exhibited superior anti-tumor efficacy compared to other targeted angiogenic inhibitors, attributable to its extensive range of kinase inhibition. Despite the tangible preclinical benefits associated with regorafenib, the identification of reliable biomarkers that can predict therapeutic responses in metastatic colorectal cancer treatments lags behind [11]. Single-agent regorafenib has been approved in Canada since March 2013 for patients with mCRC who have been previously treated with fluoropyrimidine-, oxaliplatin-, and/or irinotecan-based chemotherapy, an anti-vascular endothelial growth factor (VEGF) therapy, and if RAS wild type, an anti-epidermal growth factor receptor (EGFR) therapy. The approval of regorafenib was based on the results of the CORRECT study, an international randomized (2:1), double-blind, placebo-controlled trial that enrolled 760 mCRC patients treated with regorafenib or placebo after failure of standard therapy [12]. The trial demonstrated a statistically significant improvement in mOS and PFS in patients who received regorafenib compared to placebo (in the regorafenib arm, the mOS was 6.4 months compared to 5.0 months in the placebo arm). Additionally, the median PFS was 1.9 months with regorafenib and 1.7 months with placebo. The most frequently observed treatment-emergent adverse events (TEAEs) of Grade 3 or higher in the regorafenib group were hand-foot skin reaction (17%), fatigue (9%), diarrhea (7%), hypertension (7%), and rash or desquamation (6%). Significantly, more patients in the regorafenib group (67%) than in the placebo group (23%) had dose modifications due to adverse events (AEs). The incidence of AEs leading to permanent treatment discontinuation with regorafenib was relatively low (17.6%) compared to placebo (12.6%), indicating that most adverse reactions in the regorafenib-treated patients could be managed by dose modifications [12]. Other trials with regorafenib are in progress, including its administration in combination with FOLFIRI as second-line therapy (study NCT01298570) [13].

Currently, no clinically validated biomarkers can be used to direct the treatment of mCRC patients receiving anti-angiogenic agents. The quantification of angiogenesis, both through circulating biomarkers such as VEGF and VEGFR2 and through the analysis of angiogenesis biomarkers and other microvascular density indicators in tumor tissue, has been thoroughly investigated [14]. Despite these efforts, these approaches have not consistently demonstrated predictive value. By identifying predictive biomarkers, we can customize therapies for mCRC patients who would benefit most while protecting patients by avoiding unnecessary treatment-related toxicities. The value of biomarkers is well demonstrated by the discovery that the KRAS mutation predicted resistance to anti-EGFR antibody therapy in mCRC [15]. This represents a pioneering instance of utilizing a resistance marker in solid tumors to identify patients who would not experience any significant advantages from an expensive and potentially harmful treatment.

The efficacy of regorafenib in treating mCRC in the context of RAS or BRAF mutations remains a subject of varied and sometimes conflicting findings. Studies like those by Garcia-Alfonso et al. [16] and the subgroup study of the CORRECT trial by Tabernero et al. [17] suggest an association between these mutations and poorer outcomes or reported KRAS and PIK3CA mutations as having predictive value for treatment response, respectively. However, other research, including Ebinç et al. [18] and real-life studies by Goktas et al. [19] and Unseld et al. [20], presents differing views, ranging from no significant impact of RAS mutations on treatment efficacy to identifying RAS/RAF mutations as prognostic factors for better progression-free survival. This divergence highlights the complexity of the issue and the need for further research to attain a clearer consensus.

Similarly, our goal is to establish a prognostic biomarker signature of clinical response or resistance to regorafenib in mCRC patients that can be rapidly translated to clinical settings. To achieve this goal, we analyzed metastatic tumor tissue and serial blood samples from 47 mCRC patients who received single-agent regorafenib as second-line therapy after failing first-line therapy with an oxaliplatin and/or irinotecan-containing regimen with or without bevacizumab. This trial (Q-CROC-06) is a sequential second-line trial based on our previous Q-CROC-01 observational trial [21]. The objective of our phase II exploratory study is first to evaluate the safety and efficacy of single-agent regorafenib in second-line therapy for mCRC patients and second to identify biomarkers associated with clinical response in the same cohort using various omics approaches.

## 2. Results

The overview of study design and the consort diagram of subject disposition are presented in the supplementary information (Figures S1 and S2). The baseline demographic and clinical characteristics of mCRC patients are summarized in Table 1.

### 2.1. Efficacy

**Figure 1:**
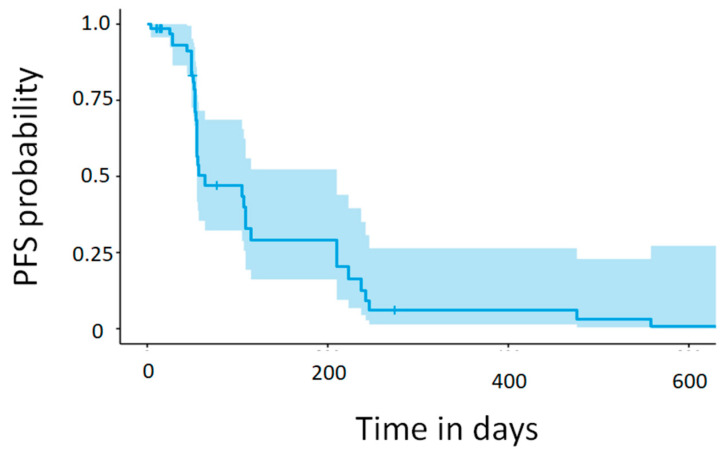
Kaplan-Meier curve for Progression-Free Survival Probability. The progression-free survival probability is depicted over time (in days) by a solid blue line. The blue shaded area indicates the 95% confidence interval and the small vertical marks on the line denote censored patients.

Of the 47 patients, 42 (89.4%) had at least one radiological evaluation after regorafenib administration. None of the patients experienced a complete response (CR); three (7.1%) had a partial response (PR), 17 (40.5%) had stable disease (SD), and 22 (52.4%) had progression of disease (PD) as their best response. The resulting objective response rate (ORR) and disease control rate (DCR) were 7.1% and 47.6%, respectively. Five patients were not evaluated as they discontinued treatment prior to their first radiological assessment (see Table S1). PFS is graphically displayed using a Kaplan-Meier curve and shown in Figure 1, and the median PFS from the curve point estimate was 1.84 months. A summary of the overall best response and response rate, DCR and PFS, is shown in Table 2.

Out of the 47 patients included in the analysis population, 37 (78.7%) provided a biopsy sample before regorafenib administration. Of these 37, three patients underwent post-regorafenib therapy biopsies, none from the same lesion as the pre-biopsy sample. Overall, a total of 40 biopsies were collected, and the majority (29, 72.5%) were collected from the liver. Lesion response was measured on pre-biopsy samples from 28 patients and on pre- and post-biopsies from different lesions from 3 patients. Each lesion’s response was objectively assessed as described in supplementary information S1 [21]. Overall, pre-biopsied lesion responses were assigned to 31 out of the 37 pre-biopsied samples as follows: 8 as intrinsic resistance (IRES), 11 as acquired resistance (ARES), and 12 as SD, representing 25.8%, 35.5%, and 38.7% of the total number of pre-biopsied lesions with an evaluable response, respectively. The lesion characteristics used for response assessment are summarized in Table S2.

### 2.2. Regorafenib Administration and Safety

Regorafenib was administered as a second-line therapy; a total of 48 patients initiated at least one cycle of regorafenib. The average number of cycles initiated per patient was 5.65, and the median was 3. The mean duration of a cycle was 29.46 days per patient. The average duration of regorafenib treatment was 5.46 months. The lowest and highest treatment durations were 0.16 and 27.29 months, respectively.

The total dose of regorafenib administered per cycle considering a 160 mg daily dose was 3360 mg, and the mean dose of regorafenib administered per cycle was 2258.01 mg. On average, the number of dose delays per cycle was 0.6 per patient. Treatment was never delayed for 8 (16.7%) patients. Thirty-one (31, 64.6%) out of 48 patients had no more than one dose delay per cycle, 6 (12.5%) had no more than two dose delays per cycle, and three (6.3%) had ≥3 dose delays per cycle. Over half of the dose delays were due to adverse events (AEs). Out of 48 patients, 9 (18.8%) had no more than one dose reduction per cycle, three patients (6.3%) had no more than two dose reductions per cycle, and one (2.1%) had ≥3 dose reductions per cycle. Regorafenib dose reduction was observed, on average, 0.24 times per cycle per patient. Thirty-five (72.9%) out of 48 patients had no dose reduction. Regorafenib dosage was reduced at least once for 9 (18.8%) patients. Three (6.3%) had a maximum of two dose reductions per cycle, and one (2.1%) had ≥3 dose delays per cycle. The number of dose escalations per cycle per patient is 0.14, and 7 (14.6%) patients had at least one dose escalation.

### 2.3. Adverse Events

All patients treated with regorafenib (N = 48) reported at least one AE, and a total of 818 AEs were reported. 760 AEs were treatment-emergent adverse events (TEAEs), with all patients (N = 48) experiencing at least one TEAE (see Table S3). TEAEs were reported most commonly from gastrointestinal disorders (38 out of 48 patients; 79.2%), general disorders and administration site conditions (36 out of 48 patients; 75.0%), skin and subcutaneous tissue disorders (32 of 48 patients; 66.7%), and musculoskeletal and connective tissue disorders (28 out of 48 patients; 58.3%). Table 3 summarizes the AEs of all dosed subjects throughout this study.

542. TEAEs were deemed related (possibly, probably, or definitely) to regorafenib and were experienced by 47 (97.9%) out of 48 patients (Table S3). A total of 43 (89.6%) patients had their treatment discontinued, interrupted, or dose reduced due to at least one TEAE. Specifically, out of 48 patients, 26 (54.2%) were discontinued from regorafenib due to at least one TEAE, 20 (41.7%) had dose reductions due to at least one TEAE, and 33 patients (68.8%) had interrupted treatment due to at least one TEAE.

Out of 760 TEAEs reported, a total of 85 (11.2%) and 14 (1.8%) were in Grade 3 and Grade 4, respectively. Most TEAEs reported were Grade 1 (443, 58.3%) and Grade 2 (174, 22.9%). Forty-four (44, 5.8%) TEAEs were graded as unknown. Two (4.2%) patients experienced only TEAE of Grade 1 severity. The highest severity of any TEAE experienced by 10 (20.8%), 29 (60.4%), and 7 (14.6%) patients was in Grade 2, Grade 3, and Grade 4, respectively. Thus, most patients experienced at least once a TEAE of Grade 3 (see Table S4).

The most frequently occurring Grade ≥3 TEAEs reported by maximum severity per patient are presented in Table 4. Overall, the most commonly reported (≥5%) Grade 3 and Grade 4 TEAE reported by patients were hypophosphatemia (8, 16.7%), hypertension (8, 16.7%), fatigue (8, 16.7%), palmar-erythrodysesthesia syndrome (7, 14.6%), diarrhea (6, 12.5%), proteinuria (5, 10.4%), platelet count decreased (4, 8.3%), and lipase increase (3, 6.3%).

A total of 19 serious adverse events (SAEs) were reported by 16 (33.3%) out of 48 patients who had received regorafenib. All SAEs were treatment-emergent (TESAE) (see Table S5). Every patient with at least one TESAE experienced withdrawal, reduction, or interruption of their study treatment. Specifically, out of 48 patients, 8 (16.7%) patients were discontinued from regorafenib due to at least one TESAE, 1 (2.1%) patient had a dose reduction due to at least one TESAE, and 9 (18.8%) patients had treatment interruptions due to at least one TESAE. The most common reported TESAEs were platelet count decreases (3 patients; 6.3%) and cholecystitis (2 patients; 4.2%).

Twenty-three (23) deaths were reported in electronic data capture (EDC). One death occurred before treatment initiation, while the other 22 occurred after treatment discontinuation. No deaths occurred within 13 days after the last dose administration and, thus, were not accounted for in the PFS analysis. The times of death from the time of treatment discontinuation are as follows: fourteen (14, 93.3%) out of the 15 deaths that occurred within first year of the last dose administered were due to the progression of the disease, and six (85.7%) out of the seven deaths that occurred after one year were due to the progression of the disease—the distribution of death time after treatment discontinuation is summarized in Table S6.

Metastatic biopsies were obtained from 37 (77.1%) out of the 48 patients treated with regorafenib before the initiation of study treatment. Biopsied lesions were collected from the liver, lung, lymph node, abdomen, or other locations. Seven (18.9%) patients out of the 37 biopsied-dosed patients reported a total of 8 AEs related to the biopsy that consisted of pain and/or bruising. None were considered serious; all were Grade 1 or 2, and none prevented the initiation of study treatment.

### 2.4. Association between Clinical Characteristics and Duration of Response

For all the patients with available PFS, we assessed the correlation between baseline characteristics and duration of response using Cox regression to evaluate possible confounding variables in the biomarker analysis. First, univariate analysis showed that patients with their primary tumor resection (*p* = 0.0042), no liver metastasis (*p* = 0.023), or not treated with bevacizumab (*p* = 0.023) had significantly higher PFS (See Table S7). Subsequently, after conducting a univariate analysis to identify significant variables, a multivariable analysis was carried out using the three variables that showed significance. Among these variables, only two, namely the presence of liver metastasis and bevacizumab treatment, remained independent predictors of PFS in the present cohort (Table S7). These predictors were then tested within a subset of the cohort (n = 29) using omics. However, in this subset analysis, none of these variables retained their statistical significance. This suggests that while the initial multivariable analysis highlighted the independent predictors of PFS, these predictors did not maintain their importance in the context of results derived from molecular data.

### 2.5. Mutational Landscape

In total, 34 metastatic specimens from 29 patients, prior to regorafenib treatment and passing quality control (histology, quality, and yield), were profiled to define the somatic mutational landscape of the cohort using whole exome sequencing (WES).

It is largely recognized that CRC cells hold driver mutations in specific genes (i.e., APC, KRAS, BRAF, PIK3CA, and TP53). Therefore, we decided to investigate the mutation spectrum of these genes in our cohort. Using the TCGA database [22], we compared the frequency of mutations of these genes in the TCGA dataset (224 primary CRC tissues) and our cohort (29 metastatic tissues from CRC patients). We observed a strong concordance between the mutation frequency in our cohort and the TCGA dataset (see Table 5). PIK3CA is the only gene in this study for which we found a lower mutation frequency than in the TCGA.

**Figure 2:**
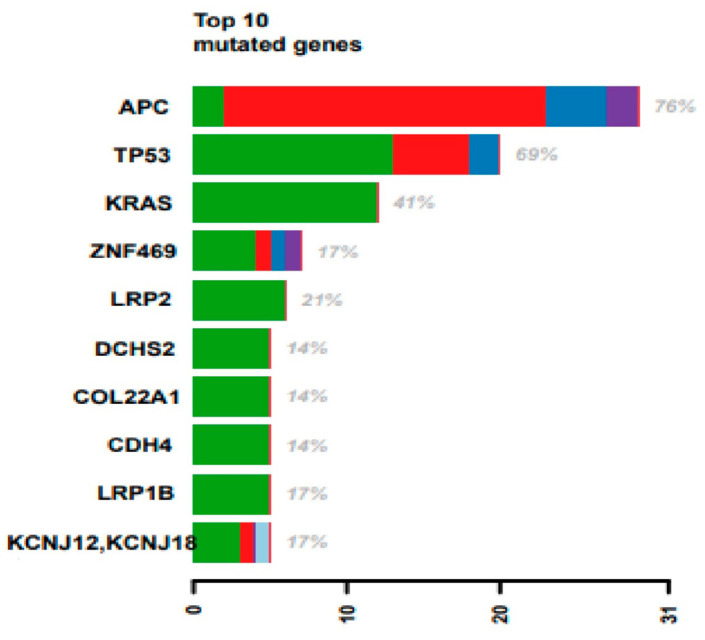
Top 10 mutated genes in the Q-CROC-06 cohort. Each row represents a gene, and the *x*-axis is the number of samples with a mutation in the gene. Green are missense mutations, blue are frameshift deletions, red are nonsense mutations, and purple are frameshift insertions (N = 29).

Despite being known drivers in CRC, BRAF and PIK3CA mutation frequencies were lower than other genes shown in Figure 2. ZNF469, LRP2, LRP1B, and KCNJ12 mutations were observed in at least 5 out of 29 patients.

The relationship between mutated genes and PFS was evaluated; none were identified as significantly associated. This could be due to the small sample size of our cohort; however, we observed that KRAS mutations in amino acids 12 or 13 were identified in 5 out of 6 patients with IRES lesions, compared to 7 out of 17 in all other lesions. A mutation in amino acid 146 was only detected in ARES lesions in 2 out of 4 patients.

To expand our analysis, we looked at the number of mutations affecting genes in the same well-described oncogenic pathways: TP53, MYC, Cell Cycle, TGF-β, PI3K, Hippo, WNT, NOTCH, and RTK-RAS. In our cohort, TGF-β is the pathway most affected by mutations (Figure S3A,B). However, we could not identify any relationship between the duration of response to regorafenib or lesion responses and these oncogenic pathways.

### 2.6. VEGF Polymorphism

The inhibition of angiogenesis through the VEGF/VEGFR pathway is part of the standard of care for most mCRC patients. Regorafenib primarily acts by targeting this angiogenic pathway. Previous publications have investigated the relationship between VEGF and VEGFR gene polymorphisms and clinical responses to anti-angiogenesis therapies [23]. This analysis aimed to report the impact of VEGF and VEGFR polymorphisms on the PFS of our cohort treated with regorafenib.

DNA from 47 patients was extracted, and eight single nucleotide polymorphisms (SNPs) located on five genes were investigated. None of the SNPs showed a significant relationship to response to regorafenib in the present cohort (see Table S8).

### 2.7. Cell-Free DNA (cfDNA) Analysis

Blood samples were taken at different time points from each patient, and cfDNA was extracted from plasma. For this analysis, only the baseline sample was profiled when patients did not provide any blood samples between the blood collection at baseline and clinical progression. For patients with an ARES overall response (i.e., one evaluation with a PR or SD overall response before PD), three time points were profiled: baseline, responses, and progression. In total, 70 samples from 39 patients had enough DNA to be profiled using the Oncomine Colon cfDNA assay, including a panel of 14 genes. Following sequencing quality control, 55 samples were left from 31 patients, and at least one mutation was detected in 38 samples from 23 patients (see Figure S4A). The lack of detectable mutations within these 17 samples could potentially signify either the absence of anomalies across the 14 genes or could be attributed to a ctDNA concentration below the assay’s detection threshold or even the restricted scope of genes included in this panel. Since not all of these patients had tumor tissue profiled by WES, it was impossible to establish a comparison of mutations identified in both assays.

**Figure 3:**
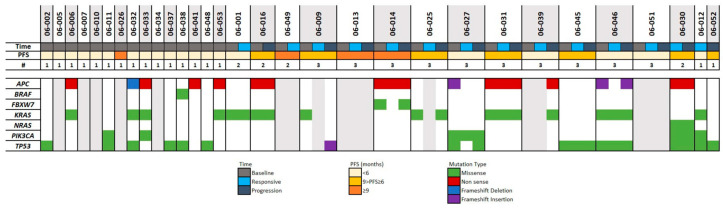
Cell-free DNA sequencing analysis. A total of 55 samples from 31 patients are presented; patients’ samples with no mutation detected despite passing all sequencing QC are depicted as a gray column, and samples with detected mutations that passed all sequencing are displayed in white. The color code for the time of blood collection, PFS, and mutation type are indicated in the legend above.

The median PFS of the cohort from the start of regorafenib treatment was 1.84 months. Interestingly, among the 31 patients with adequate cfDNA samples, we observed four patients with a remarkably durable response (PFS ≥ 9 months). When distributed in two groups based on their PFS using nine months as a cutoff (see Figure S4B), about ~18% of the patients (blue, n = 5) did not show detectable levels of ctDNA when their PFS was < 9 months, while ~70% (orange, n = 3) of the patients did not show detectable levels of ctDNA when their PFS was ≥9 months. Similar proportions were observed when ctDNA level detection was compared at the sample level using the same PFS cutoff value. The group with longer PFS (≥9 months, orange, n = 9) had a higher proportion of samples (n = 6, 67%) without detectable ctDNA than those with shorter PFS (<9 months, blue, 11 out of 46, 24%). Figure 3 shows the results for the 55 samples passing sequencing QC.

In our analysis of the six patients with serial sampling, all of whom harbored the same mutation at multiple timepoints, we closely monitored the changes in variant allelic fraction (VAF) throughout the treatment regimen. For each patient, we identified at least one mutation whose VAF diminished concurrently with the clinical response to therapy. Conversely, at the point of disease progression, these mutations exhibited an increase in VAF, as illustrated in Figure S5.

### 2.8. Copy Number Aberration (CNA) Landscape of mCRC Samples

**Figure 4:**
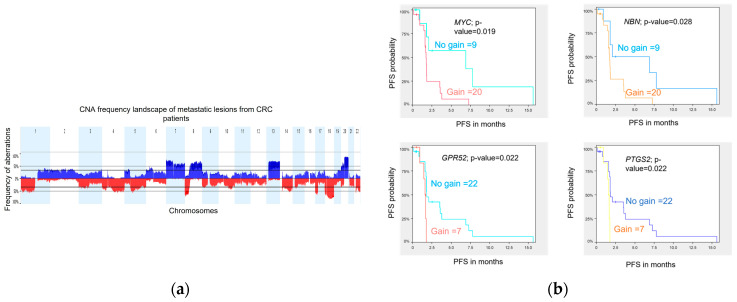
Copy number aberration profile of CRC metastatic samples and association with PFS. (**a**) The aberration frequency plot of 29 metastatic lesions shows the frequency (*y* axis) of gains in blue and losses in red as a function of the chromosome region (*x* axis). (**b**) Kaplan-Meier analyses for CN gain versus no gain of MYC, NBN, GPR52, and PTGS2.

DNA variations at the copy number level were assessed in 29 metastatic biopsies obtained prior to treatment from 29 patients. Tumor profiling was performed using WES, and analyses were conducted using Nexus Copy Number Software (version 8.0, BioDiscovery, El Segundo, CA, USA) as previously described in Gambaro et al. 2021. The genome-wide CNA landscape of the cohort is presented in Figure 4a. Overall, the chromosomal aberration frequencies were similar to those reported by the TCGA [24] and the CApecitabine, IRinotecan, and Oxaliplatin in advanced colorectal cancer (CAIRO study) [25] for primary CRC samples and also by Sveen et al. [26] for liver metastases. The most common chromosome (chr) arm amplifications were observed on chr 7p, 7q, 8q, 13q, and 20q, and the most frequent deletions were on chr 1p, 1q, 4q, 8p, 17p, 18p, 18q, and 22q. Twenty (20) significant focal aberrations (9 gains and 11 losses) were identified using the Genomic Identification of Significant Targets in Cancer (GISTIC) method [27] (Table S9).

### 2.9. Copy Number Variation Association with PFS

We investigated whether CNA in metastatic lesions could be associated with patient outcome. Using a log-rank statistic test, we identified 9 and 29 regions of copy number loss and gain, respectively, associated with PFS (permutated *p*-value ≤ 0.05). Together, these 38 significant CNA segments harbor 563 genes and miRNAs (Table S10).

Three genes, GPR52, PTGS2, and MYC, are classified as drug resistance genes in the Drug-Gene Interaction Database (DGIdb) [28], and the gene NBN, classified as clinically actionable genes, are all lying on amplified chr regions identified to be associated with a shorter PFS. Kaplan-Meier curves for each of the four genes illustrate the association between genomic amplification and shorter PFS in our cohort (Figure 4b).

### 2.10. Gene Expression

RNAseq was performed on 31 tumor tissue specimens from 29 patients. Only the 22 specimens originating from liver metastasis at baseline (prior treatment) were used for the differential expression analysis. This approach was chosen to mitigate potential noise in the analysis that could arise from incorporating tissues obtained from disparate locations (Table S11). First, we looked at differential expression between lesion response groups. Nineteen (19) of the 22 liver lesions profiled for RNAseq had evaluable responses, as follows: 7 were SD, 7 were ARES, and 5 were IRES. In one of the comparisons, SD and ARES lesions were included in the same group, as all ARES lesions could be considered SD or PR at one point during treatment. A total of 313 significantly differentially expressed genes were identified across the four comparisons (Tables S12 and S13).

**Figure 5:**
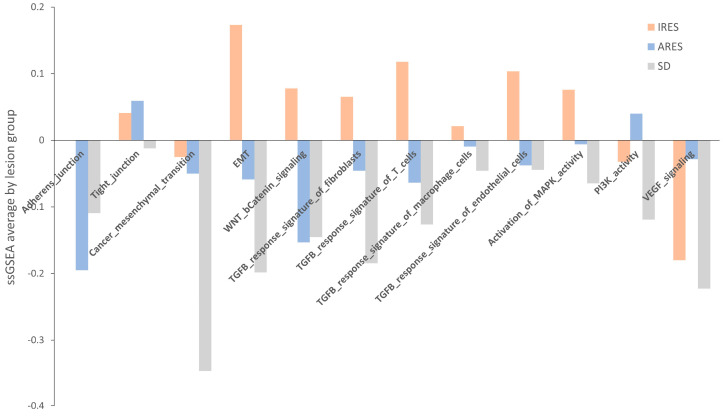
Average score of relevant gene sets per lesion response group. Twelve gene sets were investigated using the gene set enrichment analysis approach for each group of lesion responses. While no statistical differences were noted, the score in IRES lesions surpassed that of ARES and SD in several gene sets, including lesions adherens junction, EMT, TGF−β response signature, and MAPK activity.

Next, we performed a pathway enrichment analysis using a webtool “https://www.genome.jp/kegg/ (accessed on 10 June 2019)” for each group’s list of genes differentially expressed (Table S13). Overall, TGF-β signaling, MAPK signaling, and gap junction appear to be the three main pathways showing changes in gene expression between resistant and responsive lesions. Another approach to identifying a change in pathway between groups is to use a method called single sample gene set enrichment analysis (GSEA). Relevant gene sets were investigated for a difference in enrichment per lesion response. These gene sets include adherens and tight junctions, cancer mesenchymal transition, EMT, WNT, and β-catenin signaling, TGF-β response signature, activation of MAPK kinase activity, PI3K activity, and VEGF signaling (Table S14) [29,30]. A score was computed for each sample, and then the average per group was plotted and shown in Figure 5. None of the comparisons reached statistical significance, potentially due to the small sample size. However, for numerous gene sets (adherens junction, EMT, TGF-β response signature, and MAPK activity)*,* the average score in IRES lesions was superior to that of the ARES and SD lesions. In some cases, a gradient between the group scores was observed (EMT, TGF-β response signatures, and MAPK activity). Putting these results in perspective with the pathway analysis performed on differentially expressed gene lists, activation of the MAPK and the TGF-β signaling pathways were found as markers of poor response to regorafenib in both analyses.

Next, we used the same approach to identify pathways directly linked to rapid progression after regorafenib treatment. Patients were categorized into two groups according to their PFS: the “High” group comprised those with PFS ≥ 3 months, while the “Low” group encompassed patients with PFS < 3 months. One pathway, adherens junction, was identified as significantly enriched with a false discovery rate (FDR) of 0.01 in the “Low” group.

In 2015, a consensus molecular subtypes (CMS) was developed based on expression data to classify colorectal cancer patients with clear biological and clinical interpretability [31]. We used this classifier on the 22 CRC specimens originating from liver metastasis. The distribution of the four CMS was as follows: CMS1 (4.5%), CMS2 (59.1%), CMS3 (13.6%), and CMS4 (22.7%). Then, we looked at the association between CMS classification and the patient’s best overall response. No clear trend can be observed as the proportion of each subtype was very similar between patients with stable disease (SD) and progression of disease (PD). Thoroughly addressing this question may necessitate further examination involving a larger cohort.

Finally, when we look at the distribution of CMS within the Response Evaluation Criteria in Solid Tumors (RECIST) lesion response groups, the proportion of CMS4 was more prevalent in ARES (2 out of 7, 28.6%) and IRES (3 out of 5, 60%) lesions compared to stable lesions (0 out of 7, 0%) (Figure S6).

### 2.11. miRNA

Sixteen (16) liver specimens from 16 patients were used for miRNA differential expression analysis. First, we looked at differential expression between lesion response groups. Thirteen (13) of the 16 lesions profiled for miRNA had evaluable response lesions; 5 were SD, 4 were ARES, and 4 were IRES. Overall, a small number of miRNAs (n = 5) are differentially expressed in stable lesions compared to resistance lesions. Specifically, miR-365a-5p, miR-6510-3p, and miR-934 were found to be under expressed in SD in comparison to ARES or IRES, while miR-9-5p and miR-204-5p are overexpressed in SD relative to ARES or IRES. Lesions with intrinsic and acquired resistance have similar expression profiles for these miRNAs (Table S15).

## 3. Discussion

Large phase III randomized clinical trials indicated that regorafenib improved PFS and OS in refractory mCRC, with 1.4 to 2.5 months of survival and manageable adverse events [32]. The main effect of regorafenib seems to be disease stabilization rather than tumor shrinkage [12,33]. Dose modifications and discontinuations were frequent during regorafenib treatment [32].

According to the 2023 ESMO guidelines, regorafenib is recommended as a third- and further-line treatment in patients pre-treated with fluoropyrimidines, oxaliplatin, irinotecan, and biologics, if available, or in earlier lines of therapy following oxaliplatin and irinotecan regimen failure [34]. Regorafenib was also evaluated for safety and efficacy compared to other agents such as trifluridine/tipiracil (TAS-102) and fruquintinib [35,36,37]. Regorafenib and TAS-102 appeared to have similar efficacy; however, regorafenib was associated with more toxicity compared with TAS-102 [35]. The findings from the network meta-analysis of five randomized clinical trials that included 2604 pre-treated mCRC patients showed no significant difference between OS or ORR between regorafenib, fruquintinib, and TAS-102, and fruquintinib was associated with a significantly higher risk of SAEs when compared with TAS-102 or regorafenib [37]. In real-world practice, the combination of regorafenib and PD-1 inhibitors was adopted as the second-line treatment in mCRC patients and seemed to have a longer overall survival than regorafenib alone [38]. Because of regorafenib’s clinical significance as a personalized therapy, biomarker strategies are critical to guiding clinicians in patient selection.

It is important to acknowledge that our study is exploratory in nature and encompasses a limited number of patients. In this study, the observed median PFS of 1.84 months for regorafenib as a second-line therapy aligns closely with the PFS outcomes reported in the regorafenib phase III CORRECT trial (1.9 months) [12]. While challenging due to variations in patient population size, disparities in baseline characteristics, and differences in prior treatment exposure, we nonetheless observed a consistent response in both trials. It might suggest that the number of prior treatment lines received before initiating regorafenib therapy may not significantly influence treatment response. Our study demonstrated a slightly higher objective response rate than the CORRECT trial (ORR: 7.1% vs. 1.0%) and a similar disease control rate (DCR: 47.6% vs. 41%). In this study, all patients experienced at least one TEAE, and 75% of them reported a Grade 3 or 4 TEAE. The most experienced high-grade TEAEs are hypophosphatemia, hypertension, fatigue, palmar-erythrosesthesia syndrome, and diarrhea. Overall, the safety profile of regorafenib as a second-line treatment did not present unexpected findings compared to previously reported data. Our data substantiates the significance of rigorous clinical monitoring to proactively prevent and promptly identify adverse events, thereby optimizing the therapeutic benefits of regorafenib.

Overall, our study provided consistent insights into the safety profile of regorafenib as a second-line treatment while also reaffirming the feasibility of acquiring metastasis biopsies within this setting, ensuring their adequacy for subsequent comprehensive molecular profiling.

Our study has identified a list of potential prognostic biomarker candidates for response or resistance to regorafenib based on PFS and objective tumor response. At the RNA level, activation of TGF-β and MAPK signaling pathways was identified as marker candidates for poor lesion response to regorafenib, while the adherens junction pathway was significantly enriched in lesions from patients with shorter PFS. The revelation that the TGF-β pathways play a central role in conferring resistance to a variety of cancer treatments is consistent with a decade of research. Notably, this pathway has been implicated in resistance to sorafenib in hepatocellular carcinoma, among other therapies [39]. The literature describes multiple mechanisms through which TGF-β may promote resistance to BRAFi/MEKi, EGFRi, HER2i, and CDK4/6i by activating alternative signaling routes, including proliferative and anti-apoptotic pathways across different cancer types [40,41,42,43,44,45,46]. This body of work collectively suggests that the hyperactivation of alternative kinases or cellular pathways such as TGF-β and MAPK could be a key mechanism underlying the development of resistance to multikinase inhibitors [47]. However, the potential use of TGF-β and MAPK signaling activation as indicators of regorafenib resistance or response remains to be confirmed in a larger cohort.

Despite being known to be associated with a poorer prognosis, there is no clear association between CMS4 and the best overall response to regorafenib in the present cohort. The lack of a direct association between the CMS4 subtype and patient response suggests that the interplay between various genetic and molecular factors is complex and requires further exploration in larger cohorts. However, intrinsically resistant lesions were highly enriched for the CMS4 subtype. Interestingly, this study also shed light on the heightened expression of three miRNAs (miR-365a-5p, miR-6510-3p, and miR-934) and a heightened incidence of mutations affecting amino acids 12 or 13 in KRAS within resistant lesions, providing potential candidates for future research into predictors of regorafenib treatment response. Two of the miRNAs (miR-365a-5p and miR-934) have already been described as targeting genes involved in cellular proliferation and cell signaling pathways [48,49]. Their higher level of expression in resistant lesions could affect downstream targets of regorafenib inhibition and cancel its action. In the same way, KRAS mutations in resistant lesions could affect regorafenib’s efficacy by either enhancing the activation of downstream proteins, thereby overriding the drug’s inhibition, or by diminishing its impact on upstream tyrosine kinases due to the altered function of mutated KRAS [50].

The dynamics of ctDNA throughout treatment or post-tumor resection have been established as predictive indicators of patient outcome and response to therapy, as evidenced by recent studies [51,52,53,54]. Consistent with these findings, our study demonstrated a discernible relationship between the reduction in ctDNA VAF and favorable responses to regorafenib, as well as an association between increased ctDNA VAF and the progression of the disease. The observations underscore the potential of ctDNA mutation profiling as a valuable biomarker for monitoring and predicting patient responses to regorafenib over time. Complementing these insights, the proteotranscriptomic analysis presented in Papaccio et al., 2023, explored advanced CRC patient-derived organoids as a model to predict drug sensitivity and further emphasized the importance of multi-omic approaches in personalizing treatment strategies [55].

At the copy number level, we report a list of CNAs associated with PFS that can serve in the future as a basis for studies investigating chromosome aberrations as potential markers of treatment response. Among several other gene candidates, MYC amplification was associated with a shorter PFS. Amplification is the most common mechanism of alteration of MYC in solid tumors and is reported to be found in approximately 10% of CRC patients [56,57]. Previous studies have associated MYC amplification with resistance to EGFR inhibition in NSCLC and CRC [58], and it has been identified as a potential mechanism of primary resistance to the ALK inhibitor, crizotinib. As a shorter PFS is associated with primary resistance, this biomarker could also be involved in resistance to regorafenib, which warrants further validation in a larger sample set [59,60].

Our multi-omics analysis of lesions from mCRC patients treated with regorafenib as a second-line therapy has revealed several potential biomarkers indicative of poor prognosis and resistance to regorafenib. Intriguingly, many of these identified candidates have previously been linked to adverse outcomes and resistance not only in colorectal cancer but also in response to kinase inhibitors in a variety of other cancer types. This recurring pattern underscores the possibility of shared molecular mechanisms underlying resistance across different cancers and kinase inhibitor therapies. The candidates identified in our analysis, while promising, warrant further validation through studies involving larger sample sizes. This additional research is essential to confirm the reliability and clinical applicability of these biomarkers to improve patient outcomes in the context of regorafenib treatment for mCRC.

## 4. Materials and Methods

### 4.1. Study Design

This open-label, single-arm, phase II multicenter exploratory study (Q-CROC-06) enrolled patients with mCRC who had failed first-line therapy with an oxaliplatin and/or irinotecan-containing regimen with or without bevacizumab. A pre-treatment biopsy (pre-biopsy) of a liver metastatic lesion was performed on eligible mCRC patients unless they were previously enrolled in the multicenter phase IV clinical study Q-CROC-01 (NCT00984048) [61] and had provided a biopsy of a metastatic lesion after relapsing on first-line treatment. An optional biopsy at treatment resistance (post-biopsy) was also collected to explore additional putative biomarkers of resistance, and serial blood samples were collected at baseline on-study and at the time of progression. Tumor response was measured using Response Evaluation Criteria in Solid Tumors (RECIST v. 1.1). Molecular analyses were performed on patient tissue and blood samples to assess alterations prior to and following regorafenib therapy.

### 4.2. Regorafenib Treatment

A total dose of 160 mg regorafenib was administered per os once daily following a light meal for 21 days of every 28-day cycle (i.e., 21 days on, 7 days off study treatment) until one of the criteria for treatment discontinuation was met. The dose may have been delayed and/or reduced to 120 mg or 80 mg for toxicity, but it was not to be reduced by more than two dose levels. Following dose reduction, dose re-escalation was considered (up to 160 mg) at the treating physician’s discretion, provided that the toxicity had resolved to baseline.

### 4.3. Sample Size and Population

A total of 54 subjects from 6 different sites in Quebec, Canada, consented between 22 October 2013, and 27 March 2019, and 51 were eligible and enrolled for this study. Patients were recruited from multiple sites, including Jewish General Hospital (JGH), St. Mary’s Hospital (SMH), The Moncton Hospital (TMH), Hospital Notre-Dame (HND), Centre Hospitalier de l’Université de Montréal (CHUM), and McGill University Health Center (MUHC). Patients’ inclusion and exclusion criteria are listed in supplementary information S2. Three patients failed screening; one died, one withdrew consent prior to the pre-treatment biopsy, and the investigator withdrew one. Therefore, the safety population includes a total of 48 patients who received at least one dose of regorafenib. Out of the 48 patients who received regorafenib, one patient was excluded from the analysis population due to a major protocol deviation impacting response evaluations. Therefore, 47 patients were included in the efficacy and biomarker analysis populations.

### 4.4. Tissue and Blood Sample Acquisition

Tumor tissue from the largest (≥2 cm) or most easily accessible metastatic lesion (liver or other site if previously approved by the sponsor-investigator) was collected by percutaneous needle core biopsy (NCB) under radiological guidance using a biopsy gun fitted with a 16- or 18-gauge needle. A minimum of three NCBs were collected from the periphery of the lesion in an attempt to avoid the typically necrotic core. The first two biopsies were transferred to either an empty cryovial and immediately snap frozen in liquid nitrogen or a vial filled with RNA-Later RNA stabilizing reagent (Qiagen, Montreal, QC, Canada). The third biopsy was placed in a jar of neutrally buffered formalin. Samples were shipped the same day under appropriate conditions to the Q-CROC/Exactis central laboratory, where they were processed (including embedding in optimal cutting temperature [OCT] medium) for histological assessment and extraction of genomic material. Peripheral blood samples (approximately 8–16 mL) were collected in 4 mL BD Vacutainer^®^ k-EDTA blood collection tubes and immediately processed on site. Each collection tube was filled and gently inverted ten times to allow for the appropriate mixing of anticoagulant. For plasma and buffy coat collection, tubes were centrifuged (within 60 min of collection) at 1500× *g* for 15 min at room temperature. Depending on the site and their facility, either “EDTA” or “EDTA platelet poor” plasma was prepared. For “EDTA” plasma, the plasma was collected and aliquoted (500 µL aliquots) into 2.0 mL cryovials. For “EDTA platelet poor” plasma, the collected plasma was transferred to 2 mL microcentrifuge tubes and centrifuged for an additional 15 min at 2500× *g* prior to aliquoting. All aliquots were stored at −80 °C immediately after processing. Buffy coat (middle phase, grey/white interface band containing white blood cell fraction) was also collected on only one occasion (usually baseline) per patient and aliquoted into one or two cryovials prior to storage at −80 °C. Whole blood samples were also collected at baseline and stored in two 5 mL cryovials at −80 °C.

### 4.5. Sample Processing: Pathology Review and Tumor Tissue

RNAlater-submerged samples were stored at 4 °C for 72 h and then washed with RNAse-free phosphate-buffered saline for 6 min on dry ice. This last step was repeated three times. The biopsies were placed in the center of a 15 mm × 15 mm Tissue Tek disposable cryomold (Somagen, Edmonton, AB, Canada) to embed them in the OCT compound. OCT compound (Surgipath) was poured into the cryomold until the specimen was covered entirely. The cryomold was submerged for 30 s in a beaker containing 2-methylbutane (Fisher Scientific, Waltham, MA, USA) pre-cooled on dry ice, using tweezers to hold the cryomold to ensure that the sample remained horizontal. After the OCT solidified, the block was immediately stored at −80 °C. The preparation of tissue cryosections was performed using standard pathology laboratory procedures. Briefly, OCT blocks were placed for at least 30 min inside a cryostat at −20 °C prior to cutting to ensure the block’s temperature was optimal for cryosectioning. Sections 4–5 µm thick were cut onto a SuperFrost glass slide (Fisher) and stained with hematoxylin and eosin (H&E) in order to ensure the enrichment of genetic material from neoplastic cells in our samples. Histology quality control (HQC) and validation were performed on both paraffin and OCT embedded NCBs; H&E-stained sections were examined by a pathologist to determine the percentage of normal cells, tumor cells, and necrotic areas in each NCB. The threshold for acceptable tumor cell area for DNA/RNA extraction was set at >60% of the specimen, including <20% of necrotic cells, as suggested by the TCGA [24]. When needed, biopsies were macrodissected to reach these thresholds.

### 4.6. DNA and RNA Extraction from Frozen Tissue Samples

All procedures were conducted in an RNA-free environment. The OCT blocks were positioned on cold Petri dishes (placed on dry ice) with the tissue surface facing up. A sterile scalpel blade was used to remove the tumor tissue from the OCT block. If macrodissection was recommended by the pathologist (for tumor enrichment), only the portion delineated by the pathologist on the reference H&E slide was carved out and used for the extraction of genomic material. The frozen tissue was gently separated from the OCT and immediately placed in a pre-chilled Precellys^®^ CKMix homogenizing tube containing Qiagen’s RLT Plus Lysis buffer (containing 1% of 14.3 M β-mercaptoethanol). The tissue was homogenized immediately for 20s at 5000 revolutions per minute on the Precellys^®^ Evolution homogenizer (Bertin Instruments, Montigny-le-Bretonneux, France). DNA and RNA were to be isolated using the AllPrep DNA/RNA/miRNA Universal kit (Qiagen) following the manufacturer’s instructions and as described previously in Diaz et al. [62]. The NanoDrop spectrophotometer (ThermoFisher Scientific, Mississauga, ON, Canada), the Qubit v2.0 Fluorometer (ThermoFisher Scientific), the PicoGreen (ThermoFisher Scientific), and the Agilent Bioanalyzer 2100 (Agilent Technologies, Santa Clara, CA, USA) were used to assess the concentration, purity, and degradation of nucleic acid extracts. Samples with sufficient DNA quantity were used for WES profiling, and samples with an RNA integrity number (RIN) >3 were selected for total RNA sequencing (see Section 4.11).

### 4.7. DNA Extraction from Blood Samples

Frozen buffy coats and plasma samples were thawed at 37 °C and then kept on ice. DNA extraction was performed using the Gentra PureGene Blood kit (Qiagen) following the manufacturer’s instructions. The quantity of DNA was assessed using a NanoDrop^®^ spectrophotometer (ThermoFischer Scientific) by measuring absorbance at 230 nm, 260 nm, and 280 nm.

### 4.8. Sample Analysis: Whole Exome Sequencing (WES)

In total, DNA from 34 tumors and 29 buffy coat samples from 29 patients were profiled by WES in 2 batches. The library preparation was performed using SureSelect Low Input v6 + UTRs with 150 ng of DNA. Exome capture was followed by massive parallel sequencing on Illumina HiSeq 2500 and 4000 instruments with 125 base pair reads. Upon data reception, reads were processed using Trimmomatic (v0.35) [22] and the following criteria: adaptor removal, first four bases from the start of each read, and low-quality bases at the end of each read using a 4 bp sliding window to trim where average window quality fell under 30. Trimmed reads < 30 bp were discarded. The clean reads were then aligned to the reference genome hg19 (GRCh37) using BWA-MEM v0.7.13 and the -M parameter [63]. Duplicated reads were marked and filtered out so that only unique DNA fragments were used in the subsequent analysis using Picard Tool v2.1.0 “http://broadinstitute.github.io/picard/, accessed on 15 January 2019)” [64]. Using the Genome Analysis Toolkit, potential Indel were identified with the RealignerTargetCreator, and reads were realigned in these targeted regions using the IndelRealigner [65,66]. Somatic point mutation calling was conducted using MuTect2 using paired normal and tumor samples [67]. Single nucleotide variants (SNVs) were annotated using ANNOVAR [68]. As our main goal was to discover new mutations associated with mCRC response and resistance to regorafenib, we focused on variants that are not found at a high frequency rate in the general population, as reported in the 1000 Genomes Project database and the Exome Aggregation Consortium (ExAC). The following filters were used: minimum read count ≥ 10, minimum alt count ≥ 5, minimum SNV read ratio ≥ 0.05, minimum indel read ratio ≥ 0.15, and maximum MAF ≤ 0.05. Also, mutations in genes often identified in the WES experiment (FLAG genes) were excluded [69].

### 4.9. Analysis of Circulating Tumor DNA (ctDNA)

DNA was extracted from 3 mL of plasma using a QIAmp Circulating Nucleic Acid kit (Qiagen) according to the manufacturer’s instructions. Identification of mutations in 14 genes (AKT1, APC, BRAF, CTNNB1, EGFR, ERBB2, FBXW7, GNAS, KRAS, MAP2K1, NRAS, PIK3CA, SMAD4, TP53) was performed in parallel using the Oncomine Colon cfDNA assay on the Ion S5 sequencing platform. Briefly, starting with 10 ng of cfDNA, manual libraries were prepared following manufacturer instructions. Libraries were quantified using qPCR, and 80 pM of each library (24 per chip) was loaded onto a 540 chip and sequenced. Sequencing data were analyzed using the Oncomine Colon Liquid Biopsy—w1.4—DNA—Single Sample workflow. Samples with at least an average coverage depth of 10,000 passed sequencing QC. For mutations, the following filters were used: variant coverage ≥ 10, variant frequency ≥ 0.1%, and *p*-value ≤ 0.01.

### 4.10. Analysis of Copy Number Variation from WES

BAM files were pre-processed to remove duplicates and PCR artifacts using the Picard Tool function MarkDuplicates before being imported into Nexus Copy NumberTM software (version 8.0, BioDiscovery, El Segundo, CA, USA) for CNA analysis. For each sample analyzed, the matched normal DNA from blood samples derived from the same patients was used in the ngCGH (matched) processing according to the software instructions. Systematic GC wave correction was applied using Quadratic Correction. The robust variance sample quality score threshold was ≤0.2. Numerical CNAs (involving the whole chromosome arm) were manually assessed, while focal CNAs were identified by the GISTIC test (see Section 4.14). The frequency plot was obtained using the View, Aggregate, and Copy number parameters. The Nexus predictive survival tool was used to identify regions associated with PFS. Regions for which less than three patients/group had gains or losses were not included.

### 4.11. RNA Sequencing of Fresh Frozen Tissues

In total, 34 RNA samples from 29 patients were sequenced in 2 batches. All samples showed RIN > 3. Ribosomal depletion and RNA libraries were constructed with Ribo-Zero rRNA-depleted stranded (Illumina, San Diego, CA, USA) and the NEBNext adaptors, starting with 250 ng of total RNA. Libraries were sequenced as 125 bp paired-end reads on Illumina HiSeq 4000 (Genome, Quebec, QC, Canada). Reads were trimmed using Trimmomatic (v0.35) (Bolger, AM 2014 2114), removing the adaptor, the first four bases from the start of each read, and low-quality bases at the end of each read using a 4 bp sliding window to trim where average window quality fell under 30. Trimmed reads < 30 bp were discarded. Clean reads were then aligned to the reference genome hg19 (GRCh37) using STAR v2.3.0 (Dobin, A. 2013; 15). Raw gene counts were obtained using featureCount (Liao, Y. 2014-923), on the UCSC hg19 annotation. Differential expression analysis was performed using DESeq2, and the batch information was included in all designs for outcome or response group-based comparison. Genes were considered significantly differentially expressed if they met the following criteria: average expression across samples ≥ 50, |log2 Fold Change| ≥ 1, and false discovery rate (FDR) ≤ 0.05. CMS classification was performed using the CMSclassifier R package [31].

### 4.12. Small RNAseq from Fresh Frozen Tissues

In total, 24 RNA samples from 19 patients were sent for small RNAseq profiling. Total RNA samples were size-selected for miRNA, and libraries were constructed using NEBNext adaptors, followed by single-read 50 sequencing on the Ilumina HiSeq 4000. Reads were trimmed using Trimmomatic (v0.35) (Bolger, AM 2014 2114), removing adaptors and low-quality bases at the end of each read using a 4 bp sliding window to trim where average window quality fell under 30. Trimmed reads <15 bp were discarded. Clean reads were aligned using novoalign (Novocraft). Small RNA expression levels were obtained using featureCount on the hg19 miRbase annotation. Differential expression analysis was performed using DESeq2. Small RNAs were considered significantly differentially expressed if they met the following criteria: average expression across samples ≥ 50, |log2 Fold Change| ≥ 1, and FDR ≤ 0.05.

### 4.13. VEGF Polymorphism

DNA was extracted from the buffy coat of 47 patients, and 5 ng of genomic DNA was used to measure single nucleotide polymorphisms at the following sites: rs833061, rs699947, rs25648, rs4604006, rs2071559, rs2305948, rs2010963, and rs664393 using TaqMan SNP Genotyping Assays. Briefly, 5 ng of genomic DNA was used for each qPCR reaction in a final volume of 10 µL on the ABI 7500 fast machine. Genotype assignment was performed using the ABI 7500 software (Version 2.0.4).

### 4.14. Statistical Analysis

Areas of the genome with a statistically high frequency of aberration (*p*-value ≤ 0.05) were identified using the Genomic Identification of Significant Targets in Cancer (GISTIC) tool [27] in Nexus Copy Number software. The CNA frequency reported as being significantly different between the two groups met a minimum *p*-value of 0.05 on a two-tailed Fisher’s exact test. Patients who did not experience a PFS event (i.e., disease progression, clinical progression, or death) were censored. As this was a single-arm study, no comparative efficacy analyses were performed. The log-rank statistic was used to identify regions yielding a high degree of progression-free survival prediction [70]. The *p*-value is calculated by permuting the PFS time for each sample and comparing the log-rank statistic for the permuted data to the original data. The threshold used was a *p*-value ≤ 0.05. Kaplan–Meier curves were generated to compare survival times between two groups, and *p*-values were computed using the log-rank test. Cox regression was used to evaluate possible confounding variables for baseline characteristics and duration of response correlation analysis.

## 5. Conclusions

This phase II exploratory study substantiated the safety and efficacy profile of regorafenib when used as a second-line treatment. Molecular investigation of patients metastatic biospecimens pinpointed several potential biomarkers linked to unfavorable prognoses in patients with mCRC using various approaches, including pathways analysis, mutation assessment, and copy number analysis. It is worth noting that many of these identified candidates have already shown associations with poor outcomes in other cancer types or among patients undergoing different treatments. This suggests they might not serve as exclusive predictive biomarkers solely for regorafenib. An intriguing avenue for further exploration lies in delving deeper into these biomarkers’ potential relevance within the context of drug classes like tyrosine kinase inhibitors (TKIs). Since similar drug classes can involve overlapping mechanisms leading to treatment resistance, investigating these biomarkers in TKIs could yield insights into redundant factors contributing to inadequate treatment responses.

## Figures and Tables

**Table 1:** Cohort Characteristics.

Characteristic	Analysis Population N = 47	
	N	%
Age ^1^		
Median	67	N/A
Min-Max	41–89	N/A
Sex		
Male	32	68.1
Female	15	31.9
Ethnicity		
White or Caucasian	41	87.2
Asian	2	4.3
Black or African American	1	2.1
Native Hawaiian or Pacific Islander	1	2.1
Other	2	4.3
ECOG		
0	12	25.5
1	31	66
N/A	4	8.5
Stage		
IVa	13	27.7
IVb	34	72.3
Primary Tumor Histology		
Adenocarcinoma	41	87.2
Mucinous Adenocarcinoma	6	12.8
Tumor Sidedness ^2^		
Left	31	66
Right	10	21.3
N/A	6	12.8
# Metastasis Sites		
1	13	27.7
2	18	38.3
≥3	16	34.0
First-Line Regimen		
Oxaliplatin-based	29	61.7
Irinotecan-based	13	27.7
Other (oxaliplatin and irinotecan-based)	3	6.4
N/A ^3^	2	4.3
Bevacizumab		
+	22	46.8
-	23	48.9
N/A ^3^	2	4.3
Primary KRAS status		
Mutated	15	31.9
Non-mutated	18	38.3
N/A	14	29.8

^1^ Calculated age with day imputed at 15. ^2^ Sidedness was defined as right-sided when primary tumors were located from the cecum up to the transverse colon, left-sided when primary tumors were located from the splenic flexure to the rectum, and N/A for tumors of the transverse colon or of unknown location. ^3^ Patients did not receive 1st line regimen. N/A: Not Applicable or Available.

**Table 2:** Summary of Overall Best Response, Response Rate, Disease Control Rate, and Progression-Free Survival.

	Analysis Population N = 47	
	N	%
Overall Best Response ^1^		
CR	0	0
PR	3/42	7.1
SD	17/42	40.5
PD	22/42	52.4
N/A	5/47	10.6
Objective Response-Rate (CR and PR) ^1^	3/42	7.1
Disease Control Rate (CR, PR, and SD) ^1^	20/42	47.6
Progression-Free Survival		
Median (months) and 95% CI ^2^	1.84	1.74–3.58

^1^ Patients who were only assessed at baseline (N/A) were excluded from response assessment, N = 42. ^2^ The Kaplan-Meier estimate of median PFS is 1.84 months. N/A: Not Applicable.

**Table 3:** Overview of Adverse Events.

Parameter	Safety Population N = 48
Adverse Events reported, (n)	818
Patients with at least one biopsy-related adverse event, n (%)	7 (14.6%)
Treatment-emergent adverse events (TEAEs) reported, n	760
Patient with at least one TEAE, n (%)	48 (100%)
Patients with at least one drug-related TEAE ^1^, n (%)	47 (97.9%)
Patients with at least one TEAE leading to drug withdrawal, dose reduction, or interruption, n (%)	43 (89.6%)
Drug withdrawal ^2^, n (%)	26 (54.2%)
Dose reduction ^2^, n (%)	20 (41.7%)
Drug interruption ^2^, n(%)	33 (68.8%)
Treatment-emergent serious adverse events (SAEs), n	19
Patients with at least one TESAE, n (%)	16 (33.3%)
Patient with at least one drug-related TESAE, n (%)	6 (12.5%)

^1^ A TEAE was defined as study drug-related if its relationship with study drug was assessed by the Investigator as ‘Possibly’, ‘Probably’ or ‘Definitely’. ^2^ These sub-categories are not mutually exclusive.

**Table 4:** Summary of Common (>5%) Grade ≥3 Treatment-Emergent Adverse Events per Patient Using the Maximum Severity Reported (Safety Population, N = 48).

Parameter	Grade 3 n (%)	Grade 4 n (%)	Total n (%)
Hypertension	8 (16.7)	0	8 (16.7)
Hypophosphatemia	8 (16.7)	0	8 (16.7)
Fatigue	7 (14.6)	1 (2.1)	8 (16.7)
Palmar-plantar erythrodysesthesia	6 (12.5)	1 (2.1)	7 (14.6)
Diarrhea	5 (10.4)	1 (2.1)	6 (12.5)
Proteinuria	4 (8.4)	1 (2.1)	5 (10.4)
Decreased platelet count	2 (4.2)	2 (4.2)	4 (8.3)
Increased Lipase	2 (4.2)	1 (2.1)	3 (6.3)

**Table 5:** Driver mutation frequency compared to TCGA.

	Genes				
TCGA reported frequency: % in non-hypermutated tumors (n = 223)	APC	KRAS	BRAF	PIK3CA	TP53
	81%	43%	3%	18%	67%
Quebec Clinical Research Organization in Cancer (Q-CROC-06) frequency (N = 29)	76%	41%	3%	10%	69%

## Data Availability

The data presented in this study are available upon request.

## Supplementary Materials

The following supporting information can be downloaded at: https://www.mdpi.com/article/10.3390/ijms25010043/s1.
