# Supplementary material for: A Phase II Exploratory Study to Identify Biomarkers Predictive of Clinical Response to Regorafenib in Patients with Metastatic Colorectal Cancer Who Have Failed First-Line Therapy
Source: Int J Mol Sci. 2023 Dec 19;25(1):43. doi: 10.3390/ijms25010043 (PMC10778949; doi:10.3390/ijms25010043)
Supplement: Supplementary file 1 [file ijms-25-00043-s001.zip › ijms-2712159-supplementary.pdf]

[1]Supplementary information

A Phase II exploratory study to identify biomarkers predictive of clinical response to regorafenib in patients with metastatic colorectal cancer who have failed first-line therapy.

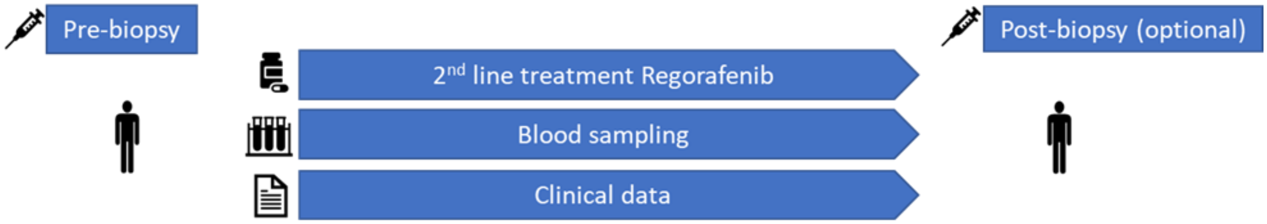

Figure S1. Overview of Study Design

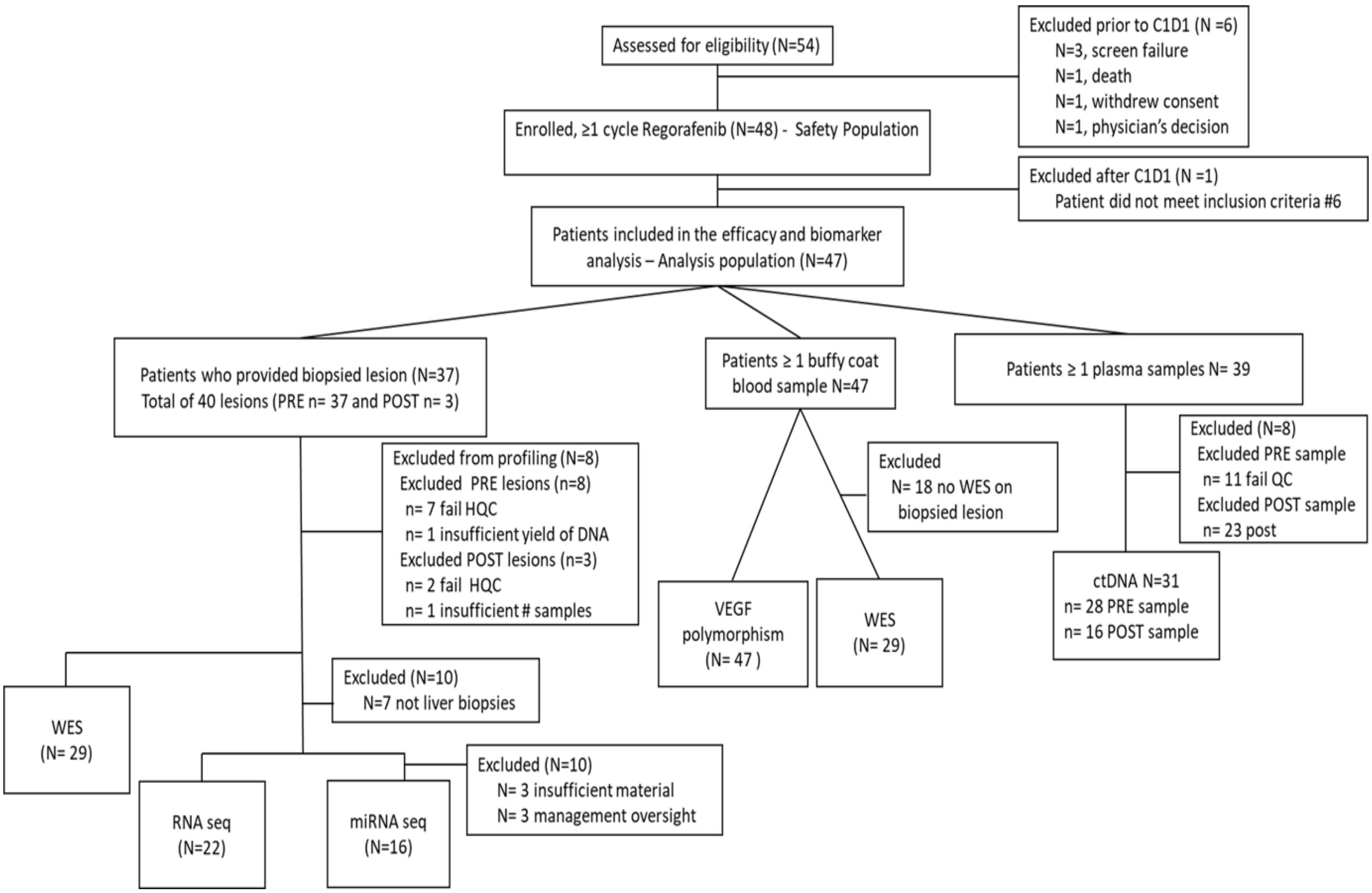

Figure S2. Consort Diagram of Subject Disposition. The number of patients is presented using N and the number of lesions is presented using n. C1D1, Cycle 1 Day 1; HQC, Histopathology Quality Control; WES, Whole Exome Sequencing, ctDNA, Circulating tumour DNA; Seq, Sequencing.

S1: Objective Response of Biopsied Lesions

At each radiological evaluation, the site staff was to capture the size of the lesion sampled at the time of the pre-treatment biopsy collection in order to follow its response to treatment. RECIST v. 1.1 guidelines were used to assess the response of the biopsied lesion as follows:

- PR: a lesion with ≥ 30% decrease in the longest diameter compared to baseline
- SD:
    - a lesion with < 30% decrease in the longest diameter compared to baseline, or
    - a lesion with < 20% increase in comparison to the smallest diameter, or
    - a lesion with ≥ 20% and < 5 mm increase in the longest diameter in comparison to the smallest diameter.
- PD: a lesion with ≥ 20% and ≥ 5 mm increase in the longest diameter in comparison to the smallest diameter.

Lesions were subsequently divided, when applicable, into two subcategories intrinsic resistant (IRES) lesions and acquired resistant (ARES) lesions which were defined as follows:

- IRES: a new lesion or a lesion with ≥ 20% and minimally by ≥ 5 mm increase in the longest diameter at the first 8-week evaluation,
- ARES: a lesion with a PR or SD response followed by a PD response. When pre- and post-biopsies were from different liver segments, the response of the lesion sampled was used.

S2: Inclusion Criteria

Patients were to meet these inclusion criteria to be enrolled in this study:

(1) Signed informed consent obtained before any study-specific procedures. Patients must have been able to understand and be willing to sign a written informed consent.

(2) Male or female patients ≥18 years of age.

(3) Histological documentation of adenocarcinoma of the colon or rectum, with at least one liver metastatic site available for biopsy (other metastatic sites may have been considered but must have been first approved by the Sponsor-Investigator).

(4) Metastatic disease not suitable for upfront curative-intent surgery.

(5) Patients must have received one (and no more than one) prior treatment regimen for metastatic CRC (i.e. FOLFOX, FOLFIRI or XELOX, with/without bevacizumab). Patients who had withdrawn from standard first-line treatment due to unacceptable toxicity before progression of disease were to be allowed into the study

(6) Measurable disease according to RECIST v. 1.1.

(7) ECOG status of ≤1.

(8) Life expectancy of at least 3 months.

(9) Women of childbearing potential and men must have agreed to use adequate contraception since signing of the informed consent form until at least 3 months after the last study drug administration. The investigator or a designated associate was requested to advise the patient on how to achiean adequate birth control. Adequate contraception was defined in the study as any medically recommended method (or combination of methods) as per the standard of care.

(10) Adequate bone marrow, liver, and renal function as assessed by the following laboratory requirements conducted within 7 days of starting to study treatment:
• Total bilirubin ≤1.5 × the upper limit of normal (ULN)
• Alanine aminotransferase (ALT) and aspartate aminotransferase (AST) ≤2 × ULN (≤5 × ULN for patients with liver involvement of their cancer)
• Alkaline phosphatase limit ≤2.5 × ULN (≤5 × ULN for patients with liver involvement of their cancer) • Amylase and lipase ≤1.5 × ULN
• Serum creatinine ≤1.5 × ULN
• International normalized ratio (INR) and partial thromboplastin time (PTT) ≤1.5 × ULN.
• Platelet count ≥100 x 109 /L, hemoglobin ≥90 g/L, absolute neutrophil count (ANC) >1.5 x 109 /L. 4.4.2.

Exclusion Criteria
Patients were to be excluded from this study if they met any of the following criteria:
(1) Previous treatment with regorafenib.
(2) Previous or concurrent cancer that was distinct in primary site or histology from colorectal cancer within 5 years before randomization, EXCEPT for curatively treated cervical cancer in situ, non-melanoma skin cancer and superficial bladder tumours.
(3) Extended field radiotherapy within 4 weeks or limited field radiotherapy within 2 weeks prior to registration. Patients had to have recovered from all therapy-related toxicities. The site of previous therapy was to have evidence of progressive disease if this was the only site of disease.
(4) Major surgical procedure or significant traumatic injury within 28 days before start of study treatment (day of first dose).
(5) Pregnancy or breast-feeding.
(6) Congestive heart failure ≥ New York Heart Association (NYHA) class 2.
(7) Unstable angina (angina symptoms at rest), new-onset angina (began within the last 3 months). Myocardial infarction less than 6 months before start of study treatment (day of first dose).
(8) Cardiac arrhythmias requiring anti-arrhythmic therapy (beta blockers or digoxin were permitted).
(9) Uncontrolled hypertension (systolic blood pressure >150 mmHg or diastolic pressure >90 mmHg despite optimal medical management).
(10) Phaeochromocytoma.
(11) Pleural effusion or ascites that caused respiratory compromise (≥ Grade 2 dyspnea per Common Terminology Criteria for Adverse Events [CTCAE] v. 4.0 or higher).
(12) Arterial or venous thrombotic or embolic events such as cerebrovascular accident (including transient ischemic attacks), deep vein thrombosis or pulmonary embolism within the 3 months before start of study treatment (day of first dose).
(13) Ongoing uncontrolled infection > Grade 2 per CTCAE.
(14) Known history of human immunodeficiency virus (HIV) infection.
(15) Active hepatitis B or C, or chronic hepatitis B or C requiring antiviral therapy.
(16) Seizure disorder requiring medication.
(17) Any history of or currently known brain metastases (patients with stable brain metastases ≥ 3 months may be eligible for the study).
(18) History of organ allograft.
(19) Evidence or history of severe bleeding diathesis.
(20) Non-healing wound, ulcer, or bone fracture.

(21) Renal failure requiring hemodialysis, peritoneal dialysis or baseline GFR < 30 mL/min/1.73m2 .
(22) Dehydration ≥ Grade 2, as per CTCAE.
(23) Substance abuse or medical, psychological, or social conditions that could have interfered with the patient's participation in the study or evaluation of the study results.
(24) Known hypersensitivity to regorafenib, regorafenib class of drugs, or excipients in the formulation.
(25) Any illness or medical conditions that were unstable or could have jeopardized the safety of the patient and his or her compliance in the study in the opinion of the investigator.
(26) Interstitial lung disease with ongoing signs and symptoms at the time of informed consent.
(27) Persistent proteinuria ≥ Grade 3 per CTCAE (i.e. >3.5 g/24 hours).
(28) Inability to swallow oral medications.
(29) Any malabsorption condition.
(30) Unresolved toxicity ≥ Grade 1, attributed to any prior therapy/procedure, excluding alopecia and oxaliplatin neurotoxicity ≤ Grade 2 (CTCAE).

A

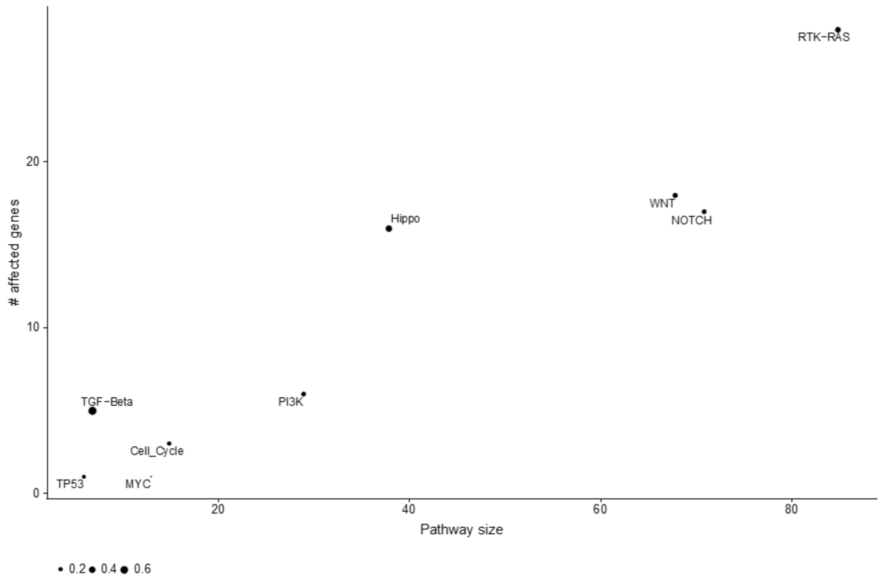

B

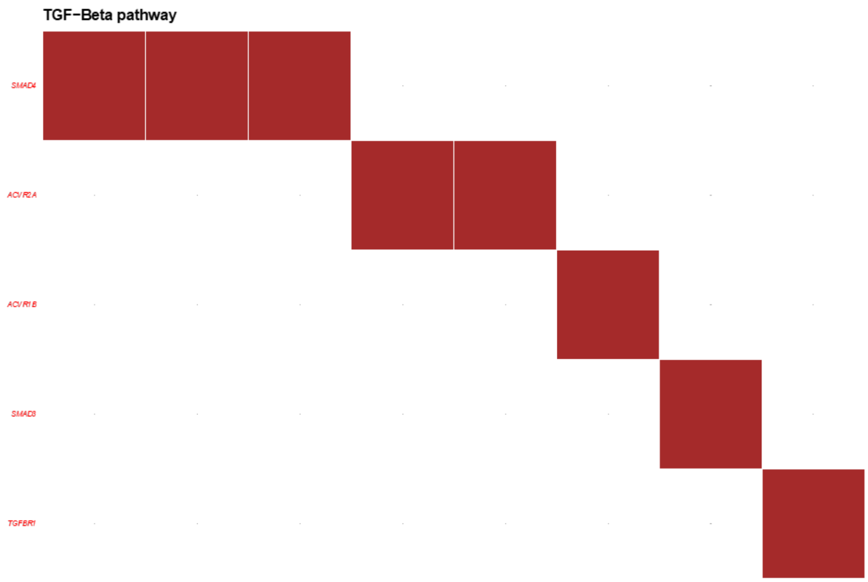

Figure S3. Visualization of mutations present in metastatic samples of the cohort by oncogenic pathways. A: The x-axis shows the number of genes in the pathways. The y-axis shows the number of genes altered in the present cohort. The size of the dot represents the proportion of genes mutated in Q-CROC-06 cohort per the total number of genes in the pathway (N=29). B. Visualization of the TGF-β pathways. Each column represents a patient and each row a gene in the TGF-β pathway. A red square is present when the gene is mutated in the patient sample.

A

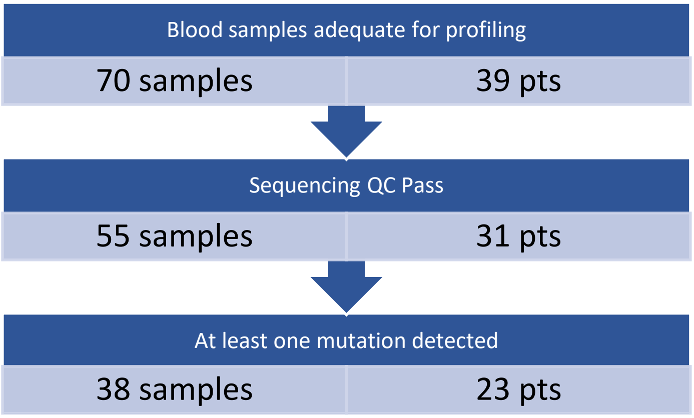

B

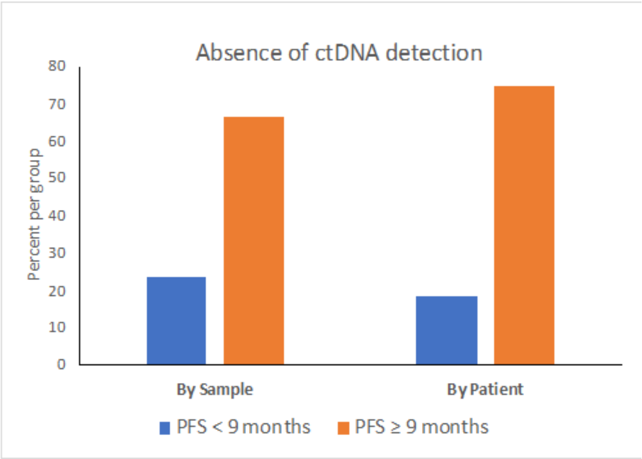

Figure S4. Cell-free DNA analysis.

A. Attrition flowchart for cfDNA profiling assay; B. Proportion of samples and patients with no ctDNA detected within each group defined using a PFS cutoff of 9 months.

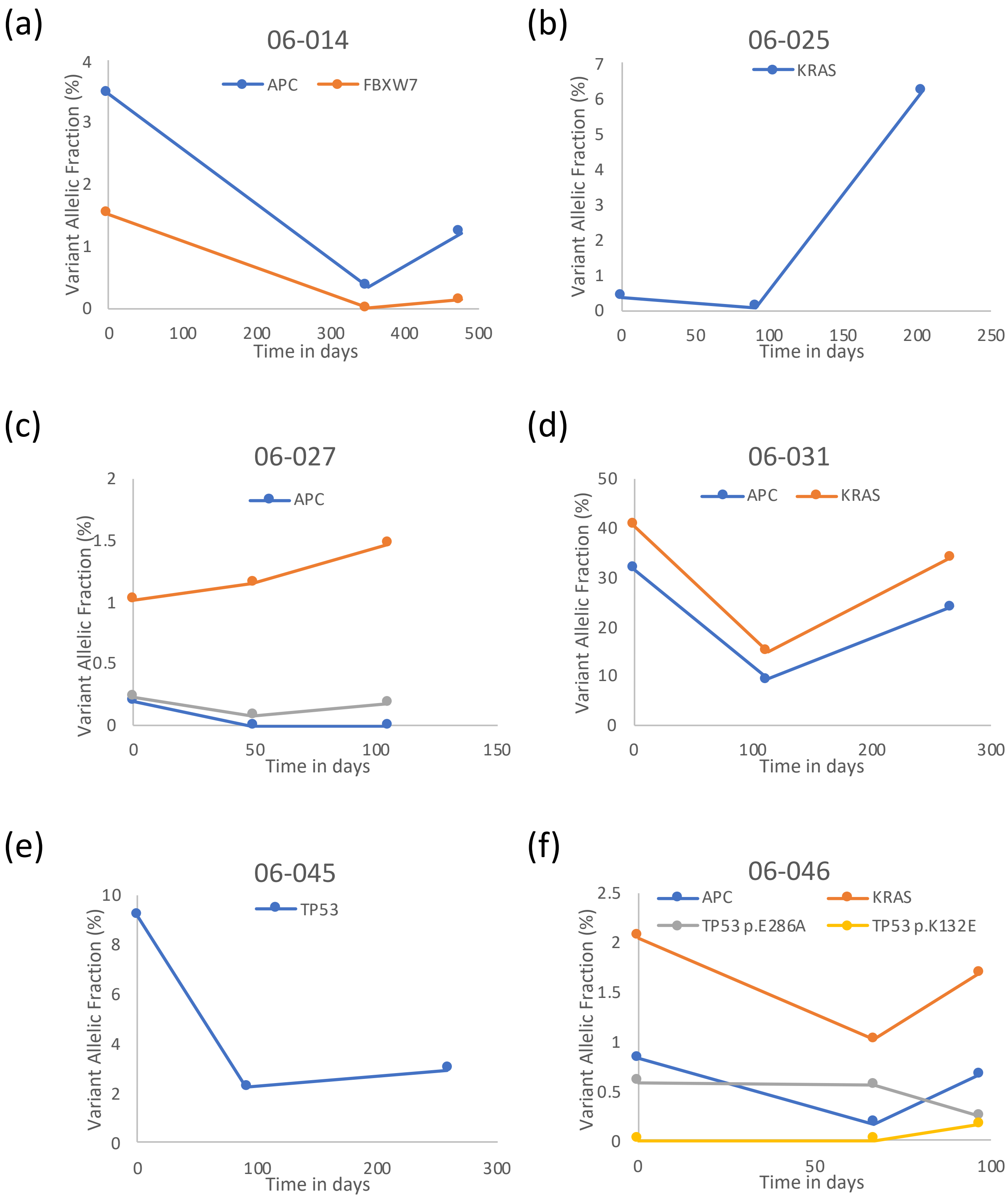

Figure S5. Variant allelic fraction dynamics during the course of treatment

a) patient 06-14, b) patient 06-025, c) patient 06-027, d) patient 06-031, e) patient 06-045, and f) patient 06-046. On the x-axis is the time in days and on the y-axis is the VAF. Each line represents a specific mutation detected as

shown in each patient legend. The first point represents time at baseline, the second time at response and the third, time of progression.

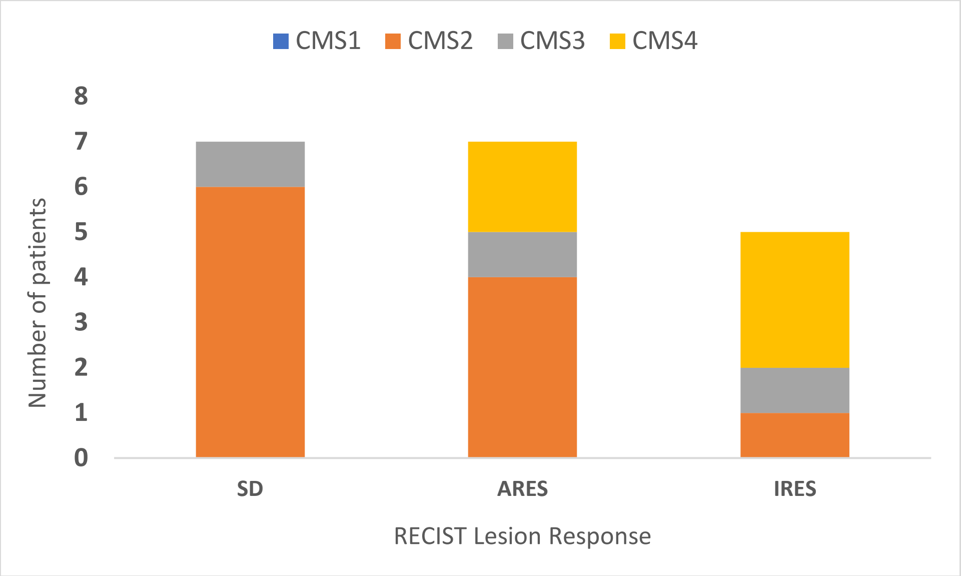

Figure S6: Distribution of Consensus Molecular Subtypes (CMS) within RECIST lesion Response Groups.

Table S1: Reasons for treatment discontinuation

Category	Overall
**Dosed patients, N**	48
**Patients withdrawn due to progression of disease, n (%)**	34 (70.8%)
**Occurrence of an unacceptable AE/toxicity, n (%)**	10 (20.8%)
**Patients withdrawn due to clinical progression, n (%)**	1 (2.1%)
**Patients request (withdrew consent), n (%)**	1 (2.1%)
**Investigator decision, n (%)**	1 (2.1%)
**Other, n (%)**	1 (2.1%)

Table S2: Summary of biopsied lesion and lesion response assessment

LESION ASSESSMENT	ANALYSIS POPULATION N=47	
	N or n[1]	%
**# Patients with > 1 biopsy sample**	N=47	
Yes	37	78.7%
No	10	21.3%

**# PRE and POST Biopsied Lesions [2]**	n=40	
prior to regorafenib initiation	37	92.5%
after regorafenib discontinuation [3]	3	7.5%
**PRE Biopsied Lesion Location[2]**	n=37	
Liver	27	73.0%
Other	9	24.3%
Unknown	1	2.7%
**PRE Biopsied Lesion Response [3,4]**	n=31	
SD	12	38.7%
ARES	11	35.5%
IRES	8	25.8%
N/A	6	N/A

[1] N refers to the number of patients and n refers to the number of biopsy samples

[2] Calculated from the total number of biopsied lesions (n=40)

[3] Pre- and post-regorafenib biopsies of the same patient (n=3) are from different lesions. Post regorafenib biopsies responses were IRES, ARES, and SD and were not used for translational analysis

[4] Calculated from the total number of pre-biopsied lesions (n=37)

[5] Calculated from the total number of pre-biopsied lesions with an evaluable response (n=31)

Table S3. Summary of Treatment-Emergent Adverse Events by SOC Experienced by ≥10% of patients.

System Organ Class<br>CTCAE Term	Safety Population (N=48)<br>n (%)
**Patients with at least one TEAE**	48 (100 %)
**Gastrointestinal Disorders**	38 (79.2 %)
**Diarrhea**	21 (43.8 %)
**Abdominal pain**	16 (33.3 %)
**Nausea**	10 (20.8 %)
**Vomiting**	10 (20.8 %)
**Mucositis oral**	8 (16.7 %)
**Constipation**	7 (14.6 %)
**Rectal hemorrhage**	6 (12.5 %)
**General disorders and administration site conditions**	36 (75.0 %)
**Fatigue**	26 (54.2 %)
**Fever**	15 (31.3 %)
**Chills**	10 (20.8 %)

System Organ Class<br>CTCAE Term	Safety Population (N=48)<br>n (%)
**Skin and subcutaneous tissue disorders**	32 (66.7 %)
Palmar-plantar erythrodysesthesia syndrome	19 (39.6 %)
Rash maculo-papular	7 (14.6 %)
Alopecia	6 (12.5 %)
Dry skin	5 (10.4 %)
Pruritus	5 (10.4 %)
Other	5 (10.4 %)
**Musculoskeletal and connective tissue disorders**	28 (58.3 %)
Arthralgia	11 (22.9 %)
Back Pain	9 (18.8 %)
Pain in extremity	9 (18.8 %)
Myalgia	8 (16.7 %)
**Metabolism and nutrition disorders**	25 (52.1 %)
Anorexia	12 (25.0 %)
Hypophosphatemia	10 (20.8 %)
Dehydration	5 (10.4 %)
**Respiratory, thoracic and mediastinal disorders**	25 (52.1 %)
Hoarseness	12 (25.0 %)
Dyspnea	8 (16.7 %)
Epistaxis	6 (12.5 %)
Cough	5 (10.4 %)
**Investigations**	24 (50.0 %)
Blood bilirubin increased	8 (16.7 %)

System Organ Class<br>CTCAE Term	Safety Population (N=48)<br>n (%)
**ALT increased**	6 (12.5 %)
**AST increased**	6 (12.5 %)
**Lipase increased**	5 (10.4 %)
**Platelet count decreased**	5 (10.4 %)
**Infections**	21 (43.8%)
**Urinary tract infection**	8 (16.7 %)
**Nervous system disorders**	21 (43.8 %)
**Headache**	10 (20.8 %)
**Paresthesia**	7 (14.6 %)
**Peripheral motor neuropathy**	5 (10.4 %)
**Vascular disorders**	19 (39.6 %)
**Hypertension**	17 (35.4 %)
**Renal and urinary disorders**	11 (22.9 %)
**Proteinuria**	8 (16.7 %)

Table S4: Incidence of Treatment-Emergent Adverse Events by Maximum Severity by Patient

Parameter	Safety Population<br>N = 48<br>n (%)
Patients with at least one TEAE [1]	47 (97.9 %)
Grade 1	2 (4.2%)
Grade 2	10 (20.8%)
Grade 3	29 (60.4 %)
Grade 4	7 (14.6%)
Grade 5	0

[1] Each patient is counted once for the maximum severity experienced (any TEAE), unknown TEAEs were not included.

Table S5: Summary of Treatment-Emergent Serious Adverse Event.

Parameter	Safety Population N = 48
Serious Adverse Events reported (n)	19
Treatment-emergent serious adverse events (n)	19
Patients with at least one biopsy-related serious adverse event (n[%])	0 (0 %)
Treatment-emergent serious adverse events reported (n)	19
Patients with at least one TESAE (n[%])	16 (33.3 %)
Patients with at least one drug-related TESAE (n[%])[1]	6 (12.5 %)
Patients with at least one TESAE leading to drug withdrawal, dose reduction or interruption (n[%])	16 (33.3 %)
Patients with at least one TESAE leading to dose interruption[1] (n[%])	9 (18.8%)
Patients with at least one TESAE leading to drug withdrawal[1] (n[%])	8 (16.7%)
Patients with at least one TESAE leading to dose reduction[1] (n[%])	1 (2.1%)

[1] Patients could have been included in more than one category.

Table S6: Distribution of Patients' Time of Death After Treatment Discontinuation (Safety Population N=48)

Months elapsed since regorafenib's last dose	Patients[1] n (%)
< 1 month	3 (6.3%)
≥ 1 and < 3 months	6 (12.5%)
≥ 3 and < 6 months	5 (10.4%)
≥ 6 and < 12 months	1 (2.1%)
≥ 12 months	7 (14.6%)

[1]For some patients, death was reported independently of study discontinuation.

Table S7: Univariate and multivariable analysis using PFS as outcome.

		UNIVARIATE		MULTIVARIABLE	
	N	HR (95% CI)	P	HR (95% CI)	P
**Age**					
< 65y	21	1			
≥ 65y	26	1.1 (0.58 – 2.2)	0.72		
**Sex**					
Female	15	1			
Male	32	1.2 (0.57 – 2.6)	0.6		
**BMI**					
< 30	30	1			
≥ 30	17	0.54 (0.27-1.1)	0.073		
**ECOG**					
0	12	1			
1	31	1.1 (0.54 – 2.4)	0.72		
**Primary Resected**					
No	9	1			
Yes	38	0.28 (0.12 – 0.67)	*0.0042**	0.5 (0.19 – 1.3)	0.158
**Sidedness**					
Left	13	1			
Right	34	1.3 (0.48 – 3.5)	0.61		
**Number of metastatic Sites**					
< 3	31	1			
3 +	16	1.9 (0.97 – 3.8)	0.062		
**Liver Metastases**					
No	12	1			
Yes	35	3.1 (1.2 – 8.1)	*0.023**	4.0 (1.40 – 11.7)	*0.01**
**Lung Metastases**					
No	23	1			
Yes	24	1.2 (0.63 – 2.4)	0.55		
**Peritoneum Metastases**					
No	37	1			
Yes	10	1 (0.46 – 2.2)	0.98		
**Other Metastatic Site**					
No	24	1			
Yes	23	1.2 (0.63 – 2.3)	0.58		
**Bevacizumab**					
No	23	1			
Yes	22	2.2 (1.1 – 4.3)	*0.023**	2.8 (1.26 – 6.1)	*0.011**

* P ≤ 0.05
HR: Hazard Ratio
CI: Confidence interval

Table S8: Summary of the genotype and univariate analysis performed on 47 patients.

Gene	SNP	Genotype	# Patient	%	Univariate	HR (95% CI)	P
VEGFA	rs25648	TT	2	4.1	CC vs TT+TC	0.93 (0.47-1.9)	0.85
		CT	15	30.6			
		CC	30	61.2			
VEGFA	rs833061	CC	10	20.4	TT+TC vs CC	1.2 (0.54-2.6)	0.67
		CT	25	51.0			
		TT	12	24.5			
VEGFA	rs699947	AA	10	20.4	CC vs AA+AC	0.93 (0.44-2)	0.86
		AC	25	51.0			
		CC	12	24.5			
VEGFA	rs2010963	CC	4	8.2	CC vs GG+GC	1.4 (0.41-4.7)	0.59
		CG	20	40.8			
		GG	23	46.9			
VEGFC	rs4604006	CC	32	65.3	TT+TC vs CC	0.99 (0.48-2.1)	0.99
		CT	15	30.6			
		TT	0	0.0			
FLT1	rs664393	CC	38	77.6	CC+CT vs TT	0.51 (0.12-2.2)	0.36
		CT	7	14.3			
		TT	2	4.1			
VEGFR2	rs2071559	AA	18	36.7	GG+AG vs AA	0.9 (0.44-1.8)	0.76
		AG	17	34.7			
		GG	12	24.5			
KDR	rs2305948	CC	41	83.7	CC vs CT+TT	1.6 (0.61-4.2)	0.34
		CT	6	12.2			
		TT	0	0.0			

Table S9. GISTIC analysis showing region focally gained or lost in 29 CRC metastatic lesions.

Only significant regions with Q-bound (FDR adjusted p-value) value ≤ 0.05 are shown. G-score: measure of the frequency of occurrence of the aberration and the magnitude of the copy number change (log ratio intensity) at each location in the aggregate of all samples in the data set.

Region	Extended Region	Type	Q-Bound	G-Score
chr12:3,603,686-4,155,279	chr12:121,792-5,153,110	CN Gain	0.00824022	8.05155565
chr13:32,402,096-32,750,036	chr13:25,264,913-98,600,139	CN Gain	1.69E-07	14.4018502
chr20:25,365,450-25,657,629	chr20:24,163,644-25,893,207	CN Gain	7.26E-05	11.0571811
chr20:34,205,363-34,740,875	chr20:29,963,172-45,811,961	CN Gain	9.44E-11	17.9885341
chr7:130,526,248-131,233,775	chr7:127,484,260-142,041,334	CN Gain	0.01169375	7.82692424
chr7:77,471,344-78,075,687	chr7:63,518,434-82,578,860	CN Gain	0.00527846	8.35501357
chr7:27,285,501-27,449,776	chr7:9,289,724-38,261,159	CN Gain	1.09E-04	10.8448921
chr8:43,033,260-43,057,692	chr8:41,753,513-43,665,344	CN Gain	3.64E-04	10.0897836
chr8:85,861,666-86,351,201	chr8:79,174,768-103,918,616	CN Gain	1.99E-06	13.1085476
chr1:57,893,600-58,948,410	chr1:42,628,447-94,578,484	CN Loss	0.00413668	8.53380572
chr10:89,728,892-90,122,400	chr10:89,631,252-93,579,279	CN Loss	8.71E-04	9.44427241
chr14:20,201,425-20,219,310	chr14:19,123,663-20,586,298	CN Loss	0.00166615	9.09233989
chr17:11,701,132-11,900,314	chr17:2,966,482-18,292,633	CN Loss	1.68E-05	11.5199467
chr18:13,491,557-13,694,281	chr18:2,537,503-15,270,970	CN Loss	1.00E-04	10.5997541
chr18:48,474,120-48,584,469	chr18:44,336,731-50,686,159	CN Loss	4.96E-13	20.2771852
chr22:16,346,117-16,449,729	chr22:16,171,502-16,688,967	CN Loss	0.00142717	9.17590884
chr4:134,111,761-138,440,204	chr4:100,851,518-158,257,339	CN Loss	0.01710582	7.52708145
chr5:69,817,965-70,070,758	chr5:69,521,493-70,671,993	CN Loss	0.01335331	7.71814656
chr8:11,987,652-11,990,735	chr8:0-21,705,632	CN Loss	5.45E-08	15.1886766
chr9:21,623,432-22,169,612	chr9:13,945,142-22,370,932	CN Loss	0.02920405	7.05118589

Table S10: Copy number variation significantly associated with progression-free survival in 29 CRC metastatic lesions.

Displaying only significant regions with a permutated p-value ≤ 0.05.

Region	Event	Length	Number of samples with the event	Median PFS (days) with the event	Number of samples without the event	Median PFS (days) without the event)	Perm P-Value	Gene Symbols	miRNAs
chr18:34304267-34802552	CN Loss	498286	23	56	6	44	0.0019	FHOD3, TPGS2, LOC105372069, KIAA1328	
chr11:48367375-48370611	CN Loss	3237	3	28	26	55	0.006568144	OR4C45	

chr1:16890840-16901409	CN Gain	10570	3	25	26	55	0.010946907	NBPF1	
chr2:240098365-240497905	CN Loss	399541	3	28	26	55	0.013683634	MGC16025, HDAC4, MIR4269, MIR2467, LOC101928111	hsa-mir-4269
chr8:120918570-121473447	CN Gain	554879	18	53	11.5	137	0.0172	DEPTOR, COL14A1, MRPL13, MTBP	
chr10:30024628-32802256	CN Gain	2777629	5	28	24	55	0.0207	SVIL, JCAD, LOC101929279, MTPAP, GOLGA2P6, MIR7162, MAP3K8, LYZL2, SVIL2P, LOC105376480, ZNF438, LOC101929352, ZEB1-AS1, ZEB1, ARHGAP12, KIF5B, EPC1, LOC102031319, LOC101929431, CCDC7	
chr1:169572294-169890531	CN Gain	318238	8	51	21	64	0.0208	SELP, SELL, SELE, METTL18, C1orf112, SCYL3, KIFAP3	
chr4:49098865-49244797	CN Gain	145933	9	49	20	64	0.0221		
chr1:155911616-156103255	CN Gain	191640	10	51	19	56	0.0229	RXFP4, MIR6738, ARHGEF2, SSR2, UBQLN4, LAMTOR2, RAB25, MEX3A, LMNA	
chr6:139036828-158520157	CN Loss	19483330	6	49	23	56	0.0233	GVQW2, CCDC28A, ECT2L, REPS1, ABRACL, HECA, TXLNB, CITED2, LINC01625, FILNC1, LOC103352541, LOC100507477, MIR3668, MIR4465, NMBR, GJE1, VTA1, ADGRG6, LOC153910, HIVEP2, LINC01277, AIG1, ADAT2, PEX3, FUCA2, PHACTR2-AS1, PHACTR2, LTV1, ZC2HC1B, PLAGL1, HYMAI, SF3B5, STX11, SNORA98, UTRN, EPM2A, FBXO30, LOC100507557, SHPRH, GRM1, RAB32, LOC101928661, ADGB, KATNBL1P6, STXBP5-AS1, LUADT1, STXBP5, SAMD5, SASH1, UST, UST-AS1, LOC105378047, TAB2-AS1, TAB2, SUMO4, ZC3H12D, PPIL4, GINM1, RPS18P9, KATNA1, LATS1, LOC645967, NUP43, PCMT1, LRP11, RAET1E, RAET1E-AS1, RAET1G, LOC105378052, ULBP2, ULBP1, RAET1K, RAET1L, ULBP3, PPP1R14C, IYD, PLEKHG1, MTHFD1L, LOC102723831, AKAP12, ZBTB2, RMND1, ARMT1, CCDC170, ESR1, MIR3163, SYNE1, SYNE1-AS1, MYCT1, VIP, FBXO5, MTRF1L, RGS17, OPRM1, IPCEF1, CNKSR3, SCAF8, MIR1273C, TIAM2, CLDN20, TFB1M, NOX3, LOC105378068, MIR1202, SNORD28B, MIR4466, ARID1B, TMEM242, ZDHHC14, MIR3692, SNX9, SYNJ2-IT1, SYNJ2	hsa-mir-1273c, hsa-mir-1202
chr6:158733977-159548166	CN Loss	814190	6	49	23	56	0.0233	SNORA116, TULP4, TMEM181, MIR7161, DYNLT1, SYTL3, MIR3918, EZR, EZR-AS1, OSTCP1, C6orf99, RSPH3, TAGAP	
chr4:49244857-49319646	CN Gain	74791	3.5	36	25.5	55	0.02583618		
chr6:51742394-53787700	CN Gain	2045309	12	51	17	56	0.0259	PKHD1, MIR206, LINCMD1, MIR133B, IL17A, IL17F, MCM3, PAQR8, EFHC1, TRAM2, TRAM2-AS1, LOC730101, TMEM14A, GSTA7P, GSTA2, GSTA1, GSTA5, GSTA3, GSTA4, ICK, FBXO9, GCM1, MIR5685, ELOVL5, RPS16P5, GCLC, LOC101927136, LINC01564, KLHL31, LRRC1	hsa-mir-206, hsa-mir-133b
chr1:175329942-178517389	CN Gain	3187449	7.5	50	21.5	60	0.0281	TNR, LINC01657, SCARNA3, COP1, PAPPA2, ASTN1, MIR488, BRINP2, LINC01645, LINC01741, SEC16B, CRYZL2P-SEC16B, CRYZL2P, RASAL2-AS1, RASAL2, TEX35, C1orf220	hsa-mir-488
chr1:181051640-182571579	CN Gain	1519941	7.5	50	21.5	60	0.0281	IER5, LINC01732, LINC01699, CACNA1E, ZNF648, LINC01344, GLUL, TEDDM1, LINC00272, RGSL1, RNASEL, RGS16	
chr8:85861666-86839229	CN Gain	977566	19	53	10	210	0.0289	LRRCC1, LOC102723322, E2F5, C8orf59, CA13, CA1, CA3, CA3-AS1, CA2, REXO1L2P	
chr7:100635850-100645140	CN Gain	9292	17	54	12	109	0.0294	MUC12	
chr8:126136286-135642910	CN Gain	9506634	20	53	9.5	175.2	0.02954	NSMCE2, TRIB1, LINC00861, LOC101927657, FAM84B, PCAT1, PCAT2, PRNCR1, CASC19, CCAT1, CASC21, CASC8, CCAT2, POU5F1B, CASC11, MYC*, MIR1204, TMEM75, PVT1, MIR1205, MIR1206, MIR1207, MIR1208, LINC00824, LINC00976, LINC00977, MIR3686, CCDC26, GSDMC, FAM49B, MIR5194, ASAP1-IT2, ASAP1, ASAP1-IT1, ADCY8, EFR3A, OC90, HHLA1, KCNQ3, HPYR1, LRRC6, TMEM71, PHF20L1, TG, MIR7848, PTCSC1, SLA, WISP1, NDRG1, ST3GAL1, LOC105375773, LOC101927798, LOC101927822, ZFAT, ZFAT-AS1	hsa-mir-1204, hsa-mir-1205, hsa-mir-1206, hsa-mir-1207, hsa-mir-1208
chr8:141568561-141889251	CN Gain	320693	17.3	53.7	11.7	210	0.029833333	AGO2, PTK2	hsa-mir-151
chr6:57213464-58609380	CN Gain	1395920	6.5	49	22.5	56	0.030925	PRIM2, MIR548U, GUSBP4, LINC00680-GUSBP4, LINC00680	hsa-mir-548u
chr18:35067671-38973978	CN Gain	3906308	3	28	26	55	0.031472359	CELF4, MIR4318, MIR924HG, MIR924, MIR5583-2, MIR5583-1, LINC01902, LINC01477	hsa-mir-4318, hsa-mir-924

chr7:101865172-101892455	CN Gain	27284	15	53	14	109	0.033	CUX1	
chr1:69736-232213	CN Gain	162478	4	49	25	55	0.0331	OR4F5, LOC729737	
chr19:11979616-12573085	CN Gain	593470	4	49	25	55	0.0331	ZNF439, ZNF69, ZNF700, ZNF763, ZNF433-AS1, ZNF433, ZNF878, ZNF844, ZNF788P, ZNF20, ZNF625-ZNF20, ZNF625, ZNF136, LOC100289333, ZNF44, ZNF563, ZNF442, ZNF799, ZNF443, ZNF709	
chr8:86841900-96166177	CN Gain	9324284	20	53	9.43	126.6	0.034614286	PSKH2, ATP6V0D2, SLC7A13, WWP1, RMDN1, CPNE3, CNGB3, CNBD1, DCAF4L2, MMP16, LOC101929709, RIPK2, OSGIN2, NBN**, DECR1, CALB1, LINC00534, LINC01030, TMEM64, NECAB1, C8orf88, PIP4P2, OTUD6B-AS1, OTUD6B, LRRC69, MIR4661, SLC26A7, RUNX1T1, LOC102724710, FLJ46284, TRIQK, MIR8084, C8orf87, LINC00535, FAM92A, RBM12B, RBM12B-AS1, TMEM67, MIR378D2, PDP1, CDH17, GEM, RAD54B, FSBP, VIRMA, LOC100288748, ESRP1, DPY19L4, INTS8, CCNE2, TP53INP1, NDUFAF6, MIR3150BHG, MIR3150B, MIR3150A, PLEKHF2	hsa-mir-3150
chr8:11990735-12009128	CN Loss	18394	19	53	10	115	0.0349	USP17L7, FAM66D, USP17L2	
chr19:8974148-10112806	CN Gain	1138661	3.7	49	25.3	55	0.035111731	MUC16, OR1M1, OR7G2, OR7G1, OR7G3, ZNF317, OR7D2, OR7D4, OR7E24, ZNF699, ZNF559, ZNF559-ZNF177, ZNF177, ZNF266, ZNF560, ZNF426, ZNF426-DT, ZNF121, ZNF561, ZNF561-AS1, ZNF562, ZNF812P, ZNF846, LOC100505555, FBXL12, UBL5, PIN1, OLFM2, COL5A3	
chr1:173056437-173154765	CN Gain	98329	7	49	22	56	0.0354	TNFSF4	
chr1:173953664-175126323	CN Gain	1172660	7	49	22	56	0.0354	RC3H1, LOC102724601, GPR52*, RABGAP1L, LOC101928696, CACYBP, MRPS14, TNN, KIAA0040	
chr1:186054498-186957787	CN Gain	903290	7	49	22	56	0.0354	HMCN1, PRG4, TPR, ODR4, OCLM, PDC, LOC102724919, PTGS2*, PACERR, PLA2G4A	
chr19:22319640-24346108	CN Gain	2026470	3.5	49	25.5	55	0.036117597	ZNF676, ZNF729, ZNF98, LOC101929124, LINC01233, GOLGA2P9, LOC100996349, LINC01785, ZNF492, ZNF99, ZNF723, ZNF728, LINC01859, LINC01858, ZNF730, ZNF724, IPO5P1, ZNF91, LINC01224, ZNF675, ZNF681, RPSAP58, ZNF726, LOC100505851, ZNF254, HAVCR1P1	
chr11:89406870-89777672	CN Gain	370803	5	49	24	55	0.0366	FOLH1B, TRIM77, TRIM49, TRIM53AP, TRIM51EP, TRIM64B, TRIM49D1, TRIM49D2, TRIM49D2, TRIM49D1, TRIM64, TRIM51EP, TRIM53AP, TRIM49C	
chr4:49244857-49320199	CN Loss	75343	3	49	26	55	0.037219485		
chr8:96289202-103404337	CN Gain	7115137	20	53	9.5	64	0.03865	C8orf37-AS1, LOC100500773, GDF6, UQCRB, MTERF3, PTDSS1, LOC102724804, SDC2, CPQ, LOC101927066, TSPYL5, SNORD3H, MTDH, LAPTM4B, MATN2, SNORA72, RPL30, ERICH5, RIDA, POP1, NIPAL2, KCNS2, STK3, OSR2, VPS13B, MIR599, MIR875, COX6C, SNORD77B, RGS22, FBXO43, POLR2K, SPAG1, RNF19A, MIR4471, ANKRD46, SNX31, MIR7705, PABPC1, YWHAZ, FLJ42969, ZNF706, NACA4P, GRHL2, NCALD, LOC104054148, MIR5680, RRM2B, UBR5-AS1, UBR5	hsa-mir-599, hsa-mir-875, hsa-mir-1273
chr7:74580831-74954762	CN Gain	373933	15	52	14.5	82.5	0.0421	NCF1C, GTF2IP4, GTF2IP1, SPDYE10P, SPDYE17, SPDYE11, SPDYE13P, SPDYE14P, SPDYE15P, CASTOR2, PMS2P5, SPDYE8P	
chr8:114388948-116424864	CN Gain	2035917	18	54	11	210	0.0438	CSMD3, TRPS1	
chr18:121171-778209	CN Loss	657041	12.7	51.7	16.3	90	0.047933333	ROCK1P1, USP14, THOC1, COLEC12, LINC01925, CETN1, CLUL1, TYMSOS, TYMS, ENOSF1, YES1	
chr4:49095653-49098814	CN Loss	3162	5	49	24	55	0.048		

Permutated p-value calculated by permuting the survival time for each sample and comparing the log-rank statistic for the permuted data to the original data.

*Drug resistance genes, ** Clinically actionable gene (based on the DGIdb[21])

Table S11. Distribution of patients per analysis and their associated PFS, lesion and overall objective response.

Pt ID	Best Overall Response	PRE_ lesion	POST_ lesion	PRE_ lesion response	POST_ lesion response	PRE_Lesion location	PFS (months)	PFS-Event Censored	WES	RNAseq	miRNA	VEGF	CTDNA_ PRE	CTDNA_ POST
01-001	SD	X	N/A	ARES	N/A	Liver, Seg. 8	3.78	No	Y	Y	N	Y	Y	Y
01-002	PD	X	N/A	SD	N/A	Liver, Seg. 4b	1.68	No	Y	Y	N	Y	Y	N
01-003	PD	X	N/A	SD	N/A	Liver, Seg. 2	0.92	No	Y	Y	N	Y	N	N
01-004	N/A	X	N/A	N/A	N/A	Liver, Seg. 1	0.46	Yes	Y	Y	Y	Y	N/A	N/A
01-005	SD	X	N/A	SD	N/A	Right Rectus Lesion	2.53	Yes	Y	Y	N	Y	Y	N
01-006	PD	X	N/A	IRES	N/A	Liver, Seg. 2 jction Seg. 3	1.84	No	Y	Y	N	Y	Y	N
01-007	PD	X	N/A	IRES	N/A	Liver, Seg. 6	1.61	No	Y	Y	Y	Y	Y	N
01-008	PD	X	N/A	IRES	N/A	Liver, Seg. 6	1.45	No	Y	Y	Y	Y	N	N
01-009	SD	N/A	N/A	N/A	N/A	N/A	7.95	No	N/A	N/A	N/A	Y	Y	Y
01-010	PD	X	N/A	N/A	N/A	Lymph node	1.61	No	Y	Y	Y	Y	Y	N
01-011	PD	X	N/A	IRES	N/A	Liver, Seg. 2/3	1.61	No	N/A	N/A	N/A	Y	Y	N
01-012	SD	N/A	N/A	N/A	N/A	N/A	3.58	No	N/A	N/A	N/A	Y	N	Y
01-013	PR	N/A	N/A	N/A	N/A	N/A	18.33	No	N/A	N/A	N/A	Y	Y	Y
01-014	SD	X	N/A	ARES	N/A	Liver, Seg. 5	15.64	No	Y	Y	N	Y	Y	Y
01-015	PD	X	N/A	IRES	N/A	Liver, Seg. 4b	1.74	No	N/A	N/A	N/A	Y	N/A	N/A
01-016	SD	X	N/A	ARES	N/A	Liver, Seg. 7	6.9	No	Y	Y	N	Y	Y	Y
16-017	PD	X	N/A	SD	N/A	Liver, Seg. 3	1.74	No	N/A	N/A	N/A	Y	N	N
15-018	N/A	X	N/A	N/A	N/A	Left upper quadrant periteonal mass	0.33	Yes	Y	Y	Y	Y	N/A	N/A

Pt ID	Best Overall Response	PRE_ lesion	POST_ lesion	PRE_ lesion response	POST_ lesion response	PRE_Lesion location	PFS (months)	PFS-Event Censored	WES	RNAseq	miRNA	VEGF	CTDNA_ PRE	CTDNA_ POST
15-020	PD	X	N/A	IRES	N/A	Liver, right lobe	0.52	Yes	Y	Y	Y	Y	N/A	N/A
01-022	PD	X	N/A	SD	N/A	Liver, Seg. 6/7	1.81	No	Y	Y	Y	Y	N	N
01-023	N/A	X	N/A	N/A	N/A	Liver, Seg. 3	0.53	Yes	Y	Y	Y	Y	N/A	N/A
06-024	PD	X	N/A	SD	N/A	Abdominal wall	1.81	No	Y	Y	Y	Y	N	N
06-025	SD	X	N/A	ARES	N/A	Liver, Seg. 7/8	6.9	No	N/A	N/A	N/A	Y	Y	Y
01-026	SD	N/A	N/A	N/A	N/A	Liver, Seg. 5	9	Yes	N/A	N/A	N/A	Y	Y	N
01-027	SD	X	N/A	ARES	N/A	Liver, Seg. 6/7	3.45	No	N/A	N/A	N/A	Y	Y	Y
06-028	SD	N/A	N/A	N/A	N/A	N/A	0.49	Yes	N/A	N/A	N/A	Y	N/A	N/A
01-030	SD	X	N/A	SD	N/A	Left lung	7.79	No	Y	Y	N	Y	N	Y
01-031	PR	X	N/A	ARES	N/A	Liver, Seg. 2	7.29	No	Y	Y	Y	Y	Y	Y
06-032	PD	N/A	N/A	N/A	N/A	N/A	1.61	No	N/A	N/A	N/A	Y	Y	N
01-033	PD	X	N/A	IRES	N/A	Abdominal wall	0.92	No	Y	Y	N	Y	Y	N
01-034	PD	N/A	N/A	N/A	N/A	N/A	1.81	No	N/A	N/A	N/A	Y	Y	N
01-035	N/A	N/A	N/A	N/A	N/A	N/A	0.36	Yes	N/A	N/A	N/A	Y	N/A	N/A
01-037	SD	N/A	N/A	N/A	N/A	N/A	1.87	No	N/A	N/A	N/A	Y	Y	N
01-038	PD	X	N/A	N/A	N/A	Unknown	1.81	No	Y	Y	N	Y	Y	N
01-039	PD	N/A	N/A	N/A	N/A	N/A	1.71	No	N/A	N/A	N/A	Y	Y	Y
01-040	PD	X	N/A	IRES	N/A	Liver, Seg. 7/8	1.61	No	Y	Y	Y	Y	N	N
06-041	PD	X	X	ARES	IRES	Liver, Seg. 5	2.1	No	Y	Y	Y	Y	Y	N
01-042	N/A	X	N/A	N/A	N/A	Liver, Seg. 8	0.13	No	Y	Y	Y	Y	N/A	N/A
01-045	SD	X	X	ARES	ARES	Transgluteal	8.08	No	N/A	N/A	N/A	Y	Y	Y
06-046	SD	X	N/A	ARES	N/A	Liver, Seg. 8	3.52	No	Y	Y	Y	Y	Y	Y

Pt ID	Best Overall Response	PRE_ lesion	POST_ lesion	PRE_ lesion response	POST_ lesion response	PRE_Lesion location	PFS (months)	PFS-Event Censored	WES	RNAseq	miRNA	VEGF	CTDNA_ PRE	CTDNA_ POST
06-047	PD	X	X	ARES	SD	Liver, Seg. 4a/2	1.77	No	Y	Y	Y	Y	N	N
06-048	PD	X	N/A	SD	N/A	Liver, Seg. 8	0.82	No	Y	Y	Y	Y	Y	N
18-049	PR	X	N/A	ARES	N/A	Omentum	20.73	No	N/A	N/A	N/A	Y	Y	Y
18-050	PD	X	N/A	SD	N/A	Liver, Seg. 5	1.74	No	Y	Y	Y	Y	N	N
18-051	SD	X	N/A	SD	N/A	Liver, Seg. 5	3.58	No	Y	Y	Y	Y	Y	Y
06-052	SD	X	N/A	SD	N/A	Peritoneal carcinomatosis. Nodules along superficial linea alba	7.33	No	N/A	N/A	N/A	Y	N	Y
06-053	SD	X	N/A	SD	N/A	Liver, Seg. 6/7	1.64	Yes	Y	Y	Y	Y	Y	N

X: Biopsy collected
PR: Partial response
SD: Progression of disease
ARES: Acquired resistance lesion
IRES: Intrinsic resistance lesion

Y: Tes, profiled and passed sequencing QC
N: No, profiled and did not pass sequencing QC
NA: Not available, sample not profiled or collected
Liver samples used for the RNAseq or miRNA analysis

Table S12: Significant differential expression of genes between each RECIST response group comparison

GROUP COMPARISON	# OF SIGNIFICANT GENES
**SD (N=7) VS ARES (N=7)**	42
**SD (N=7) VS IRES (N=5)**	76
**ARES (N=7) VS IRES (N=5)**	197
**SD+ARES (N=14) VS IRES (N=5)**	195

Table S13: List of genes differentially expressed

SD vs IRES			IRES vs ARES			SD+ARES vs IRES		
**Genes**	**log2FC**	**FDR**	**Genes**	**log2FC**	**FDR**	**Genes**	**log2FC**	**FDR**
DKK1	-8.06	4.15E-15	MAGEC1	25.24	6.12E-28	PEG10	-6.85	7.44E-11
KRT6A	-9.82	1.53E-10	MAGEC2	27.58	6.12E-28	DKK1	-6.21	3.65E-08
PEG10	-6.81	3.03E-08	MAGEA6	14.77	1.51E-14	TUBB2B	-5.61	3.49E-07
CPLX2	-6.76	5.21E-06	MAGEA2	10.67	1.08E-08	DYNC1I1	-3.70	4.52E-07
TUBB2B	-5.32	7.22E-05	PEG10	6.90	1.94E-08	LYPD3	-4.44	4.52E-07
DYNC1I1	-3.43	1.88E-04	CSAG1	12.97	7.72E-08	KRT6A	-7.73	4.97E-07
DIRAS2	-5.66	1.88E-04	MAGEA3	11.94	8.59E-08	S100A3	-5.45	1.45E-06
SLITRK6	-6.72	1.88E-04	S100A3	6.26	5.16E-07	SCG2	-4.47	2.22E-06
KRT16	-6.62	1.88E-04	DYNC1I1	4.09	8.46E-07	CPLX2	-6.09	2.24E-06
LYPD3	-4.16	1.89E-04	DKK1	5.41	1.38E-06	DIRAS2	-5.45	1.09E-05
S100A3	-5.03	2.63E-04	TUBB2B	5.96	1.49E-06	CALML5	-11.25	1.32E-05
SIX4	-3.96	2.80E-04	SCG2	5.00	4.44E-06	ASXL3	-4.87	1.96E-05
PLA2G4A	-3.21	3.01E-04	LYPD3	4.71	4.46E-06	CNTN1	-6.55	3.44E-05
CDR2L	-2.04	4.02E-04	FGF3	10.34	1.21E-05	NPTX1	-5.19	3.44E-05
ADRA2C	-4.93	5.16E-04	SLC9A4	7.32	1.89E-05	TDRD1	-6.51	4.00E-05
SCG2	-4.09	7.09E-04	ASXL3	5.46	1.89E-05	SLC1A5	-1.86	4.00E-05
TUBB2A	-3.36	9.57E-04	KRT6A	6.97	4.47E-05	TUBB2A	-3.33	5.14E-05
CALML5	-10.63	9.57E-04	IGFBPL1	5.55	4.80E-05	RIMKLA	-2.71	7.03E-05
SLC1A5	-1.85	9.57E-04	RTKN2	2.29	4.92E-05	TBX3	-1.89	9.30E-05
TBX3	-1.94	1.01E-03	TDRD1	7.30	4.92E-05	ADRB1	-4.84	2.08E-04
DEFA5	-8.08	1.35E-03	CALML5	12.32	8.09E-05	KRT5	-6.35	2.26E-04
PRSS56	-8.77	1.66E-03	IGSF1	4.73	1.17E-04	BMP5	-4.49	2.80E-04
FGF19	-6.09	1.74E-03	ANKRD34B	8.35	2.08E-04	SIX4	-3.49	2.80E-04
ASXL3	-4.45	1.74E-03	CHGA	8.29	2.08E-04	IFITM1	2.61	2.98E-04
RIMKLA	-2.63	1.99E-03	FAM46B	4.02	3.76E-04	CDR2L	-1.82	3.56E-04
B3GALT2	-4.48	2.28E-03	CPLX2	5.59	3.76E-04	SLC9A4	-5.77	4.09E-04

**TDRD1**	**-6.04**	**2.28E-03**	**MLLT11**	**3.10**	**5.34E-04**	**ECEL1**	**-5.90**	**5.52E-04**
**INHBB**	**-3.97**	**2.56E-03**	**DIRAS2**	**5.24**	**5.50E-04**	**PCDH18**	**-3.66**	**5.52E-04**
**KRT5**	**-6.39**	**2.63E-03**	**RIMKLA**	**2.79**	**6.26E-04**	**FGF3**	**-7.65**	**5.52E-04**
**AKR1B10**	**-4.75**	**2.81E-03**	**COL11A2**	**4.95**	**6.26E-04**	**MT4**	**-7.14**	**5.52E-04**
**TGFB2**	**-4.12**	**3.01E-03**	**ADAMTS20**	**8.61**	**6.26E-04**	**SRRM3**	**-3.95**	**6.42E-04**
**ADRB1**	**-4.77**	**3.29E-03**	**SOX3**	**10.63**	**7.67E-04**	**CDX2**	**2.44**	**6.42E-04**
**IL17RD**	**-4.02**	**3.68E-03**	**IFITM1**	**-2.88**	**7.67E-04**	**VGLL3**	**-3.36**	**7.51E-04**
**MSX1**	**-3.98**	**3.89E-03**	**SLC1A5**	**1.88**	**7.67E-04**	**NOTUM**	**-4.98**	**7.66E-04**
**PIAS3**	**-2.21**	**7.46E-03**	**ECEL1**	**6.64**	**7.89E-04**	**VSTM2L**	**-4.14**	**7.66E-04**
**VGLL3**	**-3.37**	**7.46E-03**	**PCDH20**	**-3.68**	**7.95E-04**	**KRT16**	**-5.39**	**1.14E-03**
**NOTUM**	**-4.97**	**8.68E-03**	**NEB**	**3.99**	**8.23E-04**	**VSIG8**	**-4.55**	**1.21E-03**
**IGFBP3**	**-2.00**	**9.07E-03**	**TUBB2A**	**3.31**	**1.01E-03**	**CLDN5**	**-3.47**	**1.30E-03**
**PCDH18**	**-3.60**	**9.56E-03**	**PRDX5**	**-2.21**	**1.04E-03**	**TGFB2**	**-3.78**	**1.38E-03**
**DBNDD1**	**-1.56**	**1.09E-02**	**REXO1L1**	**10.79**	**1.26E-03**	**LYPD8**	**6.65**	**1.38E-03**
**TRIM62**	**-2.41**	**1.11E-02**	**LGSN**	**5.31**	**1.56E-03**	**FAM46B**	**-3.33**	**1.55E-03**
**SLC9A4**	**-5.14**	**1.15E-02**	**NEUROD1**	**6.39**	**1.63E-03**	**MLLT11**	**-2.62**	**1.63E-03**
**FGF3**	**-6.93**	**1.15E-02**	**ADRB1**	**4.92**	**1.72E-03**	**SGCE**	**-2.51**	**1.77E-03**
**HIST2H3D**	**-1.94**	**1.20E-02**	**ZNF8**	**1.17**	**1.72E-03**	**CT45A5**	**-7.94**	**1.80E-03**
**PROM2**	**-2.75**	**1.20E-02**	**TBX3**	**1.83**	**2.29E-03**	**RAC3**	**-2.21**	**1.90E-03**
**TMEM158**	**-4.20**	**1.31E-02**	**SOX21**	**7.62**	**2.30E-03**	**GPR155**	**-3.17**	**1.97E-03**
**ECEL1**	**-5.48**	**1.32E-02**	**RASL11B**	**4.01**	**3.04E-03**	**RELL1**	**-2.08**	**1.97E-03**
**GADD45A**	**-2.03**	**1.64E-02**	**KRT5**	**6.32**	**3.04E-03**	**BAMBI**	**-3.60**	**1.97E-03**
**GPR155**	**-3.12**	**1.64E-02**	**VSIG8**	**4.92**	**3.19E-03**	**PIGR**	**5.01**	**2.03E-03**
**FGFRL1**	**-2.13**	**1.64E-02**	**KCNK2**	**7.05**	**3.42E-03**	**TMEM158**	**-4.09**	**2.37E-03**
**RELL1**	**-2.06**	**1.64E-02**	**COMMD3-BMI1**	**1.09**	**3.42E-03**	**PLA2G4A**	**-2.59**	**2.44E-03**
**FSCN1**	**-2.89**	**1.64E-02**	**GPR108**	**-0.96**	**3.42E-03**	**ALDH3B2**	**-3.90**	**2.44E-03**
**DUSP4**	**-2.59**	**1.64E-02**	**SCN5A**	**5.46**	**3.62E-03**	**ARTN**	**-2.78**	**2.56E-03**
**BAMBI**	**-3.56**	**1.64E-02**	**ZNF716**	**8.65**	**3.66E-03**	**PIAS3**	**-2.03**	**2.66E-03**

**VSIG8**	**-4.31**	**1.68E-02**	**CDX2**	**-2.51**	**4.70E-03**	**SLC39A2**	**5.00**	**2.66E-03**
**OSBPL6**	**-3.03**	**1.68E-02**	**ARTN**	**3.05**	**4.83E-03**	**ASPSCR1**	**-2.24**	**2.66E-03**
**SGCE**	**-2.47**	**1.68E-02**	**HHIPL2**	**3.43**	**5.12E-03**	**INHBB**	**-3.41**	**2.78E-03**
**ASPSCR1**	**-2.26**	**1.68E-02**	**SLC4A3**	**3.94**	**5.12E-03**	**C6orf222**	**2.91**	**2.81E-03**
**TTLL7**	**-3.30**	**1.82E-02**	**PCDH18**	**3.73**	**5.12E-03**	**FSCN1**	**-2.81**	**3.19E-03**
**ID3**	**-2.44**	**2.06E-02**	**C18orf54**	**1.77**	**5.12E-03**	**ZIC1**	**-5.22**	**3.31E-03**
**RAC3**	**-2.12**	**2.31E-02**	**SLC38A11**	**4.89**	**5.26E-03**	**GJB6**	**-4.81**	**3.41E-03**
**SMAD6**	**-1.56**	**2.43E-02**	**PTPRZ1**	**6.57**	**5.35E-03**	**APCDD1**	**-3.73**	**3.51E-03**
**SERPINI1**	**-3.28**	**2.44E-02**	**PCDHB2**	**4.02**	**5.35E-03**	**TRIM62**	**-2.24**	**3.55E-03**
**GNAI1**	**-3.11**	**2.44E-02**	**CASP5**	**-3.12**	**5.35E-03**	**AKR1B10**	**-4.07**	**3.56E-03**
**AGPAT9**	**-3.27**	**2.51E-02**	**CRB3**	**-1.03**	**5.35E-03**	**PRDX5**	**1.86**	**3.75E-03**
**RASL11B**	**-3.36**	**2.68E-02**	**COL4A6**	**7.66**	**6.23E-03**	**CYP24A1**	**-5.03**	**4.22E-03**
**APCDD1**	**-3.54**	**3.47E-02**	**ARHGEF18**	**-1.05**	**6.58E-03**	**CASP5**	**2.81**	**4.32E-03**
**FAM46B**	**-2.91**	**3.63E-02**	**VGLL3**	**3.35**	**6.88E-03**	**C18orf54**	**-1.58**	**4.39E-03**
**PRAME**	**-3.91**	**3.63E-02**	**CTPS1**	**1.31**	**7.18E-03**	**B3GNT3**	**1.76**	**4.46E-03**
**FAHD2B**	**-1.81**	**3.78E-02**	**DLX5**	**7.90**	**7.18E-03**	**OSBPL6**	**-2.88**	**4.60E-03**
**MLLT11**	**-2.27**	**3.83E-02**	**RAC3**	**2.29**	**7.32E-03**	**FAHD2B**	**-1.84**	**4.72E-03**
**ARTN**	**-2.55**	**4.25E-02**	**ZNF300**	**3.24**	**7.47E-03**	**PRAME**	**-3.98**	**4.72E-03**
**TMEM163**	**-2.82**	**4.25E-02**	**MRAP2**	**-3.75**	**7.80E-03**	**FIGN**	**-2.69**	**4.80E-03**
**CEP72**	**-2.48**	**4.61E-02**	**SIX4**	**3.15**	**7.80E-03**	**GPD1L**	**1.29**	**4.91E-03**
**GSTA1**	**-3.75**	**4.61E-02**	**NOTUM**	**4.99**	**8.02E-03**	**SLC4A3**	**-3.47**	**5.23E-03**
**TNFRSF19**	**-3.17**	**4.74E-02**	**MOBP**	**5.03**	**8.66E-03**	**PCDH20**	**3.13**	**5.30E-03**
			**SGCE**	**2.57**	**8.66E-03**	**FABP3**	**3.11**	**5.35E-03**
			**NXPE4**	**-5.51**	**8.66E-03**	**B3GALT2**	**-3.69**	**5.35E-03**
			**CDR2L**	**1.63**	**8.66E-03**	**ADAMTS14**	**-3.35**	**5.40E-03**
			**SULT1E1**	**5.54**	**9.04E-03**	**CTPS1**	**-1.16**	**5.45E-03**
			**OR13H1**	**4.90**	**9.64E-03**	**ADRA2C**	**-3.81**	**5.65E-03**
			**APCDD1**	**4.00**	**9.64E-03**	**HIST2H3D**	**-1.75**	**6.37E-03**

			GPR155	3.24	1.05E-02	DBNDD1	-1.39	6.37E-03
			S100A7	7.00	1.06E-02	RASL11B	-3.65	6.75E-03
			SERPINB13	8.68	1.06E-02	LYZL2	-4.14	7.02E-03
			BEST3	4.78	1.09E-02	IGFBPL1	-3.91	7.09E-03
			C6orf222	-3.05	1.15E-02	POU3F2	-5.83	7.51E-03
			BAMBI	3.64	1.22E-02	LNP1	-1.29	8.81E-03
			RELL1	2.10	1.22E-02	DAZ4	-7.74	8.93E-03
			SLC39A2	-5.12	1.24E-02	TMEM163	-2.77	9.53E-03
			TTL	1.36	1.24E-02	AGPAT9	-3.05	9.53E-03
			ADAMTS14	3.61	1.25E-02	NXPE4	4.80	9.61E-03
			SPECC1L	1.21	1.26E-02	TSPYL5	-2.95	9.73E-03
			TMPRSS11E	5.32	1.38E-02	ID3	-2.25	9.93E-03
			LRRC31	-3.04	1.39E-02	IL3RA	1.53	1.04E-02
			SEMA3D	3.49	1.39E-02	FUT1	-1.03	1.04E-02
			SELENBP1	-1.98	1.39E-02	KRTAP19-1	-5.87	1.04E-02
			LRRN1	4.41	1.44E-02	SLC38A5	3.38	1.06E-02
			KITLG	2.11	1.44E-02	GADD45A	-1.81	1.07E-02
			OTOP1	8.27	1.50E-02	SPRR3	-6.76	1.07E-02
			HSD17B14	1.54	1.50E-02	FCGBP	4.71	1.07E-02
			S100A4	2.23	1.51E-02	KITLG	-1.88	1.11E-02
			PIR	1.95	1.56E-02	GRAMD2	2.55	1.11E-02
			B4GALNT3	-2.21	1.64E-02	TRIM40	2.72	1.11E-02
			FRMD1	-3.75	1.70E-02	C10orf99	4.44	1.11E-02
			SLC6A15	9.05	1.71E-02	MISP	1.44	1.13E-02
			ATP4A	5.49	1.71E-02	MSX1	-3.22	1.17E-02
			FUT1	1.10	1.71E-02	GPR98	-3.34	1.17E-02
			PIGR	-4.89	1.76E-02	FRMD1	3.36	1.20E-02
			CXorf48	4.96	1.76E-02	SERPINI1	-2.99	1.20E-02

			B3GNT3	-1.82	1.78E-02	FGF19	-4.64	1.32E-02
			TMEM158	4.00	1.78E-02	HOXC8	-3.69	1.32E-02
			ASPSCR1	2.22	1.78E-02	MCOLN2	2.53	1.40E-02
			PRAME	4.07	1.93E-02	SDR16C5	-3.37	1.40E-02
			FAHD2B	1.87	1.94E-02	G6PD	-1.75	1.40E-02
			SBSN	5.53	1.94E-02	SCN5A	-4.36	1.46E-02
			EPHB3	-2.53	1.96E-02	S100A7	-5.62	1.50E-02
			SRPK1	-0.94	1.96E-02	GNAI1	-2.80	1.50E-02
			AKR1B15	4.50	1.96E-02	NWD1	2.61	1.50E-02
			KRT16	4.71	1.96E-02	CDK5R1	-2.06	1.73E-02
			RAB3D	-1.49	2.10E-02	UGT2B17	4.02	1.74E-02
			S100PBP	1.13	2.11E-02	DLL4	1.21	1.76E-02
			CHRM5	3.20	2.11E-02	RPS28	1.42	1.83E-02
			CCDC88B	-2.41	2.19E-02	SOX21	-5.63	1.87E-02
			FCHO1	-1.93	2.24E-02	HIST1H3E	-2.30	1.93E-02
			CEP192	1.39	2.39E-02	DEFA5	-5.89	2.07E-02
			IL3RA	-1.60	2.53E-02	PTGDR	3.44	2.10E-02
			PTGDR	-3.85	2.53E-02	NAA40	-1.23	2.12E-02
			PDE4A	-1.68	2.53E-02	LMO4	-2.16	2.12E-02
			CERCAM	1.89	2.57E-02	LY6G6F	3.21	2.14E-02
			TGFB2	3.38	2.65E-02	TTLL7	-2.81	2.21E-02
			RFX8	2.12	2.65E-02	DLX5	-6.09	2.21E-02
			CXCR4	1.95	2.65E-02	SMAD6	-1.36	2.21E-02
			NRARP	-1.64	2.65E-02	CEACAM7	4.47	2.21E-02
			UNC13C	5.84	2.65E-02	CYP26B1	-2.18	2.31E-02
			FSCN1	2.69	2.71E-02	TRIP13	-1.24	2.31E-02
			NWD1	-2.79	2.73E-02	DNALI1	-2.39	2.33E-02
			INSM1	5.42	2.73E-02	PRSS56	-6.28	2.42E-02

			FBXL14	-1.78	2.74E-02	CDHR1	3.37	2.42E-02
			CHORDC1	1.16	2.82E-02	C17orf78	-4.88	2.49E-02
			C10orf71	7.52	2.82E-02	EREG	2.66	2.52E-02
			CT45A5	8.91	2.83E-02	AP3M2	-1.12	2.55E-02
			FAIM2	-2.99	2.89E-02	RPL36	1.35	2.55E-02
			TPM4	-0.94	2.91E-02	FCHO1	1.71	2.55E-02
			LNP1	1.33	2.91E-02	CXCR4	-1.71	2.60E-02
			PIAS3	1.87	2.97E-02	SOD3	2.24	2.63E-02
			CT45A4	8.27	2.97E-02	MICU3	-2.00	2.63E-02
			LYZL2	4.33	3.09E-02	FOXD4L2	-3.01	2.72E-02
			KREMEN2	3.09	3.09E-02	POF1B	1.05	2.77E-02
			TMPRSS5	3.86	3.11E-02	PLAGL1	1.88	2.79E-02
			CEACAM7	-4.92	3.14E-02	PTBP2	-1.17	2.85E-02
			DCAF4L2	9.17	3.22E-02	NAPG	-1.22	2.87E-02
			CDH19	3.49	3.25E-02	COL4A6	-5.81	2.88E-02
			SLC38A5	-3.49	3.30E-02	MRAP2	3.01	2.88E-02
			OPRD1	4.29	3.31E-02	CEP72	-2.21	2.90E-02
			TRIM62	2.08	3.31E-02	PPM1L	-1.84	2.94E-02
			NR2C1	1.10	3.56E-02	IL17RD	-3.02	2.95E-02
			CTLA4	3.41	3.70E-02	NOS2	2.80	2.97E-02
			TMEM173	-1.55	3.75E-02	LY6G6D	3.36	3.15E-02
			ANKRD35	4.07	3.80E-02	SLITRK6	-4.27	3.16E-02
			OSBPL6	2.71	3.80E-02	GPAM	-1.55	3.22E-02
			SMIM10	2.12	3.80E-02	CDC45	-1.08	3.33E-02
			NAPG	1.36	3.80E-02	ZDHHC11	-2.42	3.35E-02
			TPRN	-1.42	3.81E-02	TPRN	1.24	3.35E-02
			EMB	2.81	3.93E-02	DIO3	2.65	3.35E-02
			GRAMD2	-2.57	3.96E-02	LPAR2	-2.23	3.35E-02

			ZNF280B	1.56	3.96E-02	DYNC2H1	-1.17	3.44E-02
			GPD1L	-1.23	3.96E-02	GGT6	2.61	3.55E-02
			AKR1B10	3.68	3.96E-02	DUSP4	-2.08	3.62E-02
			TSPYL5	2.94	3.97E-02	NRARP	1.40	3.69E-02
			LCN2	-2.63	4.01E-02	SLC12A2	1.36	3.84E-02
			KLHL14	4.12	4.02E-02	SLC26A2	2.45	3.84E-02
			ARHGEF40	-1.36	4.11E-02	CSRNP3	-2.45	4.13E-02
			NDST4	5.64	4.13E-02	PIR	-1.59	4.13E-02
			C10orf99	-4.48	4.13E-02	EID3	-1.74	4.13E-02
			KHNYN	-0.95	4.17E-02	MTSS1L	-1.57	4.13E-02
			UNC80	4.22	4.26E-02	CDH19	-2.99	4.13E-02
			AP1M2	-1.25	4.26E-02	KIF1A	-4.73	4.16E-02
			CCNE1	1.93	4.26E-02	GPR64	3.80	4.16E-02
			SPRR3	6.97	4.30E-02	CLN3	1.36	4.40E-02
			TMEM163	2.70	4.30E-02	ARL4C	-1.35	4.51E-02
			SOWAHB	-1.25	4.30E-02	SERPINB4	-4.07	4.56E-02
			ATP1B4	7.84	4.30E-02	REEP1	2.90	4.56E-02
			CYP26B1	2.33	4.39E-02	TMPRSS3	-1.75	4.72E-02
			MCOLN2	-2.57	4.45E-02	UBA52	1.02	4.78E-02
			NRSN1	5.52	4.45E-02	HOMER1	-1.53	4.80E-02
			PLA2G4A	2.12	4.47E-02	BATF2	1.97	4.81E-02
			BMI1	1.10	4.50E-02	PTPRZ1	-4.71	4.84E-02
			INHBB	2.98	4.72E-02	CCDC88B	2.00	4.84E-02
			LMO4	2.27	4.79E-02	PRDM7	-3.91	4.84E-02
			B3GALT2	3.31	4.79E-02	LRRN1	-3.42	4.85E-02
			ZDHHC11	2.65	4.79E-02	NSG1	-3.29	4.85E-02
			CDC45	1.16	4.79E-02	ZNF439	1.59	4.85E-02
			DLL4	-1.25	4.89E-02	TRNP1	-2.10	4.88E-02

			NT5DC2	1.58	4.94E-02	SLC38A11	-3.52	4.95E-02
			KIF21B	-1.70	4.98E-02			
			GOLPH3L	-0.92	4.98E-02			

Table S14: List of genes in the gene set

Adherens_junction	Tight_junction	Cancer_mesenchymal_transition	EMT	WNT_bCatenin_signaling	TGFB_response_signature_of_fibroblasts	TGFB_response_signature_of_T_cells	TGFB_response_signature_of_macrophage_cells	TGFB_response_signature_of_endothelial_cells	Activation_of_MAPK_activity	PI3K_activity	VEGF_signaling
NECTIN1	CRB3	ACTA2	ABI3BP	ADAM17	FLT1	TIMP1	C5orf23	ASAP1	ADAM8	ATM	FIGF
NECTIN2	CLDN4	ADAM12	ACTA2	AXIN1	FLT1	RAB31	RAI14	SAV1	ADORA2B	BTC	FLT1
NECTIN3	CLDN3	AEBP1	ADAM12	AXIN2	COL10A1	CLIC4	NA	WWTR1	ADRA2A	CD19	FLT4
NECTIN4	CLDN7	ASPN	ANPEP	CCND2	IGFBP3	MXRA7	AHNAK2	ZNF532	ADRA2B	CD28	KDR
PARD3	CLDN19	BGN	APLP1	CSNK1E	NOX4	SERPINE1	ZNF532	ARL4C	ADRA2C	CD80	NRP1
SRC	CLDN16	C1QTNF3	AREG	CTNNB1	MEX3B	INPP5F	CEP170	PALM2-AKAP2	ALK	CD86	NRP2
FARP2	CLDN14	C7orf10	BASP1	CUL1	GAS1	GEM	POSTN	NRP1	ARRB1	EGF	PDGFC
CDC42	CLDN15	CDH11	BDNF	DKK1	INHBA	RAB31	PALM2-AKAP2	CEP170	AVPI1	EGFR	VEGFA
RAC1	CLDN17	COL10A1	BGN	DKK4	VEGFA	SERPINE1	CEP170	BNIP3L	AXIN1	ERBB2	VEGFB
RAC2	CLDN20	COL11A1	BMP1	DLL1	CDKN2B	ANXA5	SPP1	RBMS1	BIRC7	ERBB3	VEGFC
RAC3	CLDN11	COL1A1	CADM1	DVL2	NA	RAB31	NA	MYOF	BMP2	ERBB4	
WAS	CLDN18	COL1A2	CALD1	FRAT1	FBXO32	LMCD1	VEGFA	TGFB1	C1QTNF2	EREG	
WASL	CLDN22	COL3A1	CALU	FZD1	NA	CST6	ERRFI1	HIP1	C5	FGF1	
IQGAP1	CLDN5	COL5A1	CAP2	FZD8	CALB2	RBPJ	APOE	RBMS1	C5AR1	FGF10	
BAIAP2	CLDN10	COL5A2	CAPG	GNAI1	CTGF	RASGRP3	FBXO32	ANGPT2	CCL19	FGF16	
WASF1	CLDN8	COL6A2	CD44	HDAC11	VEGFA	CLIC4	PCOLCE2	CDH2	CD40LG	FGF17	
WASF2	CLDN6	COL6A3	CD59	HDAC2	VEGFA	PLAU	EHD2	VEGFA	CD74	FGF18	
WASF3	CLDN2	COMP	CDH11	HDAC5	KANK4	LOH3CR2A	NA	UGCG	CD81	FGF19	
AFDN	CLDN1	COPZ2	CDH2	HEY1	NET1	ITGAV	ENO2	PLOD2	CDK1	FGF2	
LMO7	CLDN9	CRISPLD2	CDH6	HEY2	HEY1	MXRA7	KCNJ8	IDS	CHRNA7	FGF20	
SSX2IP	CLDN23	CTSK	COL11A1	JAG1	SERPINE1	MAP4	PLAUR	RNF13	CSPG4	FGF22	

**SORBS1**	**CLDN25**	**DCN**	**COL12A1**	**JAG2**	**ESM1**	**MAP4**	**TANC2**	**SPOCK1**	**CXCR4**	**FGF23**	
**ACTN1**	**CLDN24**	**EDNRA**	**COL16A1**	**KAT2A**	**NET1**	**KLF7**	**VCAN**	**WWC2**	**DAB2IP**	**FGF3**	
**ACTN4**	**OCLN**	**EPYC**	**COL1A1**	**LEF1**	**NOX4**	**ABHD2**	**NA**	**FAP**	**DAXX**	**FGF4**	
**VCL**	**F11R**	**FAP**	**COL1A2**	**MAML1**	**TIMP3**	**BMP1**	**NA**	**RBMS1**	**DBNL**	**FGF5**	
**TJP1**	**JAM2**	**FBN1**	**COL3A1**	**MYC**	**SYNE1**	**KCNK1**	**PLAUR**	**EMP1**	**DRD4**	**FGF6**	
**CDH1**	**JAM3**	**FN1**	**COL4A1**	**NCOR2**	**BHLHE40**	**BMP1**	**CD109**	**COL5A2**	**DUSP5**	**FGF7**	
**CTNND1**	**BVES**	**GLT8D2**	**COL4A2**	**NCSTN**	**PLAUR**	**RGS16**	**NA**	**ALCAM**	**DUSP6**	**FGF8**	
**CTNNB1**	**CDC42**	**GREM1**	**COL5A1**	**NKD1**	**APBB2**	**ATXN1**	**VCAN**	**VCAN**	**DUSP7**	**FGF9**	
**CTNNA3**	**PARD6A**	**HNT**	**COL5A2**	**NOTCH1**	**FGF1**	**CST6**	**CDKN1C**	**VCAN**	**DUSP9**	**FGFR1**	
**CTNNA1**	**PARD6G**	**INHBA**	**COL5A3**	**NOTCH4**	**TIMP3**	**NMB**	**VEGFA**	**CALD1**	**EFNA1**	**FGFR2**	
**CTNNA2**	**PARD6B**	**ITGBL1**	**COL6A2**	**NUMB**	**SERPINE1**	**KCNK1**	**PLA2G16**	**AKAP13**	**EGF**	**FGFR3**	
**ACTB**	**MPP5**	**LGALS1**	**COL6A3**	**PPARD**	**ANGPTL4**	**PLAU**	**AXL**	**CHMP2B**	**EPGN**	**FGFR4**	
**ACTG1**	**MPP4**	**LOX**	**COL7A1**	**PSEN2**	**LMCD1**	**RGS16**	**NA**	**STX7**	**ERCC6**	**FRS2**	
**RHOA**	**TJP3**	**LOXL2**	**COL8A2**	**PTCH1**	**NA**	**MAP4**	**KANK4**	**MAP1B**	**ERN1**	**FYN**	
**PTPRM**	**PATJ**	**LRRC15**	**COMP**	**RBPJ**	**FGF1**	**BMP1**	**PLOD2**	**DCBLD2**	**ERP29**	**GAB1**	
**PTPRB**	**MPDZ**	**LUM**	**COPA**	**SKP2**	**SYNE1**	**EGR2**	**ULBP2**	**PTRF**	**FCER1A**	**GRB2**	
**PTPRF**	**PRKCZ**	**MFAP5**	**CRLF1**	**TCF7**	**PGM2L1**	**IL1RN**	**RASSF8**	**FBN1**	**FGF10**	**HBEGF**	
**PTPN1**	**PRKCI**	**MMP11**	**CTGF**	**TP53**	**KAL1**	**MAP4**	**TBC1D8**	**LRRFIP1**	**FGF2**	**IRS1**	
**PTPN6**	**AMOT**	**MMP2**	**CTHRC1**	**WNT1**	**TNFAIP6**	**ABHD2**	**SERPINE1**	**SLC25A36**	**FPR1**	**IRS2**	
**PTPRJ**	**AMOTL1**	**MXRA5**	**CXCL1**	**WNT5B**	**OSGIN2**	**ATXN1**	**SOX11**	**SULF1**	**FRS2**	**KIT**	
**CSNK2A1**	**AMOTL2**	**NID2**	**CXCL12**	**WNT6**	**PODXL**	**IL1RN**	**VCAN**	**10-Sep**	**GAB1**	**KITLG**	
**CSNK2A2**	**ARHGAP17**	**NOX4**	**CXCL6**		**PCDH9**	**CLIC4**	**DOCK4**	**CALD1**	**GHR**	**KL**	
**CSNK2A3**	**RAC1**	**NUAK1**	**CYR61**		**C13orf33**	**KLF10**	**SULF1**	**ACVR1**	**GHRL**	**KLB**	

**CSNK2B**	**NF2**	**OLFML2B**	**DAB2**		**RASGRP3**	**APOD**	**IER5L**	**PAPPA**	**GNG3**	**LCK**	
**TCF7**	**LLGL2**	**PCOLCE**	**DCN**		**LOH3CR2A**	**IL1RN**	**C15orf52**	**GNB5**	**GRM1**	**NRG1**	
**TCF7L1**	**LLGL1**	**PDGFRB**	**DKK1**		**SPSB1**	**KLF7**	**FGD6**	**CAMSAP1L1**	**GRM4**	**NRG2**	
**TCF7L2**	**DLG1**	**PLAU**	**DPYSL3**		**FN1**	**LMCD1**	**PDLIM7**	**QKI**	**HGF**	**NRG4**	
**LEF1**	**SCRIB**	**POSTN**	**DST**		**GADD45B**	**PIK3IP1**	**EBF1**	**PALLD**	**IKBKG**	**PDGFA**	
**IGF1R**	**PPP2CA**	**PRRX1**	**ECM1**		**TRIB1**	**CRIP1**	**AMIGO2**	**TNFRSF21**	**IL1B**	**PDGFB**	
**INSR**	**PPP2CB**	**RAB31**	**ECM2**		**HEY1**	**ATXN1**	**NA**	**NRP2**	**INSR**	**PDGFRA**	
**MET**	**PPP2R1B**	**RCN3**	**EDIL3**		**STK17B**	**SLC5A3**	**NA**	**UBE2H**	**IQGAP3**	**PDGFRB**	
**EGFR**	**PPP2R1A**	**SERPINF1**	**EFEMP2**		**KLF7**	**NR4A3**	**NA**	**FERMT2**	**IRAK1**	**PIK3C2A**	
**ERBB2**	**PPP2R2A**	**SFRP4**	**ELN**		**LRRC8C**	**EVI2A**	**PTPRM**	**CDC42BPA**	**IRAK2**	**PIK3C2B**	
**FGFR1**	**PPP2R2B**	**SNAI2**	**EMP3**		**FNIP2**	**FAM102A**	**VCAN**	**ELK3**	**ITGA1**	**PIK3C2G**	
**FYN**	**PPP2R2C**	**SPARC**	**ENO2**		**TGFB2**	**TIAM1**	**BHLHE40**	**CALU**	**KIAA1804**	**PIK3C3**	
**YES1**	**PPP2R2D**	**SPOCK1**	**FAP**		**FRMD4A**	**ALOX5AP**	**BACH2**	**OSBPL1A**	**KIT**	**PIK3CA**	
**MAPK1**	**PARD3**	**SULF1**	**FAS**		**NA**	**ATXN1**	**OLFML2B**	**NRP1**	**LPAR1**	**PIK3CB**	
**MAPK3**	**TIAM1**	**THBS2**	**FBLN1**		**CNTN1**	**ZNF365**	**PLAUR**	**CLIC4**	**LPAR3**	**PIK3CD**	
**SNAI2**	**TJP1**	**THY1**	**FBLN2**		**TIMP3**	**MAP4**	**NA**	**SMAD3**	**LRRK2**	**PIK3CG**	
**SNAI1**	**TJAP1**	**TIMP3**	**FBLN5**		**APBB2**	**PIK3IP1**	**APBB2**	**ANGPT2**	**MADD**	**PIK3R1**	
**TGFBR1**	**DLG2**	**TMEM158**	**FBN1**		**NGF**	**SERPINE1**	**MFGE8**	**PICALM**	**MAP2K1**	**PIK3R2**	
**TGFBR2**	**DLG3**	**TNFAIP6**	**FBN2**		**NUAK1**	**MXRA7**	**MYADM**	**PLAU**	**MAP2K2**	**PIK3R3**	
**SMAD2**	**NEDD4**	**VCAN**	**FERMT2**		**NA**	**SLC5A3**	**SLC16A6**	**FN1**	**MAP2K3**	**PIK3R4**	
**SMAD3**	**NEDD4L**		**FGF2**		**TNFAIP6**	**RBPJ**	**NA**	**RHOQ**	**MAP2K5**	**PIK3R5**	
**SMAD4**	**CGN**		**FLNA**		**NA**	**TNFSF4**	**SERPINE1**	**LRP12**	**MAP2K6**	**PIK3R6**	
**CREBBP**	**CGNL1**		**FMOD**		**EFNB2**	**PPAP2A**	**ACTN1**	**FGFR1**	**MAP2K7**	**PTPN11**	

EP300	ARHGEF2		FN1		TSPAN2	PKIA	OLR1	BAG2	MAP3K10	TLR9	
MAP3K7	RHOA		FOXC2		NA	PKIA	DPP4	RHOQ	MAP3K11	TRAT1	
NLK	GATA4		FSTL1		FN1	FBXO11	CD109	CCDC88A	MAP3K2	VAV1	
FER	MARVELD3		FSTL3		CHST11	DCLRE1C	TRNP1	KLF7	MAP3K5		
ACP1	MAP3K1		FUCA1		NA	ABHD2	IGFBP4	ANXA3	MAP3K6		
	MAPK8		FZD8		STK17B	SOX4	B3GALNT1	UBE2H	MAP3K7		
	MAPK10		GADD45A		VEGFA	C18orf1	PCDHB2	PTPRR	MAP3K9		
	MAPK9		GADD45B		BHLHE40	RBPJ	NA	RDX	MAP4K1		
	JUN		GAS1		EGR2	SLC5A3	ABLIM3	MPDZ	MAP4K2		
	CD1A		GEM		DAAM1	BMP1	NA	TRAM1	MAP4K5		
	CD1B		GJA1		PALLD	NPTX1	LMTK3	COL4A2	MAPK1		
	CD1C		GLIPR1		FNIP2	ABHD2	NA	CALD1	MAPK10		
	CD1D		GLT25D1		GPR161	BMP1	HTRA1	STAG2	MAPK11		
	CD1E		GPC1		CALD1	SLC5A3	NA	TPM4	MAPK14		
	CFTR		GPX7		LOC728449	ABHD2	CLDN11	BGN	MAPK3		
	CDK4		GREM1		TIMP3	CSGALNACT1	TMCC3	NOTCH2	MAPK8IP3		
	YBX3		HTRA1		FNIP2	IL1RN	DPYSL3	BMPR2	MAPKAPK2		
	SYMPK		ID2		NA	CSGALNACT1	ADAMTS2	TM6SF1	MAPKAPK3		
	PCNA		IGFBP2		MGC16121	TMOD1	LOC641518	RRAS2	MAPKAPK5		
	CCND1		IGFBP3		HIVEP2	ABHD2	MYH10	MEF2A	MDFI		
	ERBB2		IGFBP4		RHOU	SOX4	CST6	ZNF281	MDFIC		
	RUNX1		IL15		KDM6B	KCNK1	SKI	CALCRL	MOS		
	HSPA4		IL32		FOXP1	SOX4	ELK3	UBE2W	MUC20		

	**SLC9A3R1**		**IL6**		**ELMOD1**	**ATP1B1**	**FGD6**	**PTPN14**	**MUL1**		
	**EZR**		**IL8**		**SEMA7A**	**CCR4**	**PHLDA1**	**DPYSL3**	**NOD1**		
	**RDX**		**INHBA**		**PTHLH**	**CD83**	**NA**	**IGFBP5**	**NOD2**		
	**MSN**		**ITGA2**		**PMEPA1**	**LRIG1**	**SLC16A6**	**GPR176**	**NTF3**		
	**PRKCE**		**ITGA5**		**APBB2**	**FBXO11**	**KAL1**	**FADS3**	**NTRK3**		
	**ACTB**		**ITGAV**		**CDKN2B**	**ADO**	**CCDC88A**	**TRIB2**	**P2RX7**		
	**ACTG1**		**ITGB1**		**DAAM1**	**LRBA**	**CYTH3**	**FN1**	**PAK3**		
	**CACNA1D**		**ITGB3**		**HAS2**	**LMCD1**	**ATP10A**	**COL8A1**	**PDE6G**		
	**MAP3K5**		**ITGB5**		**TGFB2**	**SOX4**	**ITGA5**	**ODZ3**	**PDE6H**		
	**MAP2K7**		**JUN**		**ANGPTL4**	**DIXDC1**	**PLOD2**	**MACF1**	**PEA15**		
	**SRC**		**LAMA1**		**KLF7**	**PPAP2A**	**SLC43A3**	**TMEFF1**	**PIK3CB**		
	**CTTN**		**LAMA2**		**KDM6B**	**SOX4**	**EBF1**	**AKT3**	**PKN1**		
	**HCLS1**		**LAMA3**		**LMCD1**	**IL1RN**	**ZNF281**	**MACF1**	**PLA2G1B**		
	**ACTR2**		**LAMC1**		**PGM2L1**	**SOX4**	**ADAM12**	**MBNL2**	**PLCE1**		
	**ACTR3B**		**LAMC2**		**SNORD114-3**	**SYNJ2**	**HSPG2**	**MACF1**	**PRKAA1**		
	**ACTR3C**		**LEPRE1**		**NA**	**DIXDC1**	**PTPRM**	**LAMA4**	**PROK1**		
	**ACTR3**		**LGALS1**		**GRB14**	**NR4A3**	**MSR1**	**TRAK1**	**PROK2**		
	**WHAMM**		**LOX**		**LIF**	**RIMS3**	**LAMC1**	**ABCA1**	**PTPLAD1**		
	**WAS**		**LOXL1**		**PTHLH**	**JUN**	**APOC1**	**EMCN**	**PTPN1**		
	**WASL**		**LOXL2**		**CHST11**	**CD96**	**PDLIM4**	**AIDA**	**PTPN11**		
	**VASP**		**LRP1**		**TSHZ3**	**DCLRE1C**	**NA**	**FAM129A**	**RIPK1**		
	**PRKACA**		**LRRC15**		**PDLIM4**	**CD96**	**VSIG10L**	**THBS1**	**RIPK2**		
	**PRKACB**		**LUM**		**TSPAN2**	**KLF7**	**PACSIN3**	**RDX**	**RPS27A**		

	PRKACG		MAGEE1		FOXP1	INPP5F	B3GALNT1	PIK3CA	S1PR2		
	RAB13		MATN2		LOC728264	PRKCD	LARP6	SERPINB2	SAA1		
	ARHGEF18		MATN3		TSHZ3	S100PBP	THBS1	PERP	SHC1		
	ROCK1		MCM7		ZNF365	LRBA	CXCR7	MCTP1	SHC2		
	ROCK2		MEST		HAS2	S100PBP	PDLIM7	PDP1	SOD1		
	MYL2		MFAP5		APBB2	HLF	GABARAPL1	OSMR	SPAG9		
	EPB41L4B		MGP		SERPINE1	MAP4	SLC11A1	POLR2K	SYK		
	STK11		MMP1		PDGFC	HLF	NA	MAP1LC3B	TAB1		
	PRKAA1		MMP14		JUNB	ABCC4	APOC1	EZR	TAB2		
	PRKAA2		MMP2		PMEPA1	HLF	GADD45B	FERMT2	TAB3		
	PRKAB1		MMP3		OSGIN2	INPP5F	BTG1	ADAM10	TDGF1		
	PRKAB2		MSX1		CILP	SYNJ2	MMP8	BMPR2	TGFA		
	PRKAG1		MXRA5		PGM2L1	KCNK1	ELK3	ARPP19	TGFB3		
	PRKAG3		MYL9		NA	CXCL13	ITGAV	CLIC4	THBS1		
	PRKAG2		MYLK		KDM6B	PPARG	TANC2	LTBP1	TLR4		
	MYH15		NID2		NA	ABCC4	SPHK1	CLDND1	TNF		
	MYH1		NNMT		NA	ADO	GDF5	DUSP3	TNFSF11		
	MYH2		NOTCH2		BPGM	AHCYL2	ABHD2	TXNIP	TP73		
	MYH3		NT5E		ARHGEF3	FBXO11	HPGD	SUSD5	TRAF6		
	MYH4		NTM		PGBD5	SYNJ2	KIAA1217	ZEB1	UBA52		
	MYH8		OXTR		TAGLN3	AQP3	PECAM1	BACH1	UBB		
	MYH9		PCOLCE		PTHLH	PIK3IP1	APOE	EZR	UBC		
	MYH10		PCOLCE2		TUFT1	BMP1	DPP4	AZIN1	WNT5A		

	**MYH11**		**PDGFRB**		**GPR183**	**AQP3**	**PECAM1**	**MAP4K5**	**ZAK**		
	**MYH7B**		**PDLIM4**		**S1PR5**	**DIXDC1**	**PDE4DIP**	**DGKA**			
	**MYH14**		**PFN2**		**STK17B**	**C18orf1**	**GADD45B**	**LEPR**			
	**MYH13**		**PLAUR**		**CLDN4**	**JUN**	**NA**	**PRKCH**			
	**MYL6B**		**PLOD1**		**MBOAT2**	**TSPAN13**	**FLCN**	**PLEC**			
	**MYL6**		**PLOD2**		**CNNM4**	**MAP4**	**THBS3**	**TCF7L2**			
	**MYL9**		**PLOD3**		**PMEPA1**	**AHCYL2**	**CXCR4**	**TPM4**			
	**MYL12B**		**PMEPA1**		**CALD1**	**SLC1A4**	**GPX3**	**BCAT1**			
	**MYL12A**		**PMP22**		**DNAJB5**	**ADO**	**TM6SF1**	**LBH**			
	**IGSF5**		**POSTN**		**NA**	**TFEB**	**DLC1**	**PEA15**			
	**MAGI1**		**PPIB**		**C3orf52**	**C18orf1**	**FKBP1B**	**LEPR**			
	**SYNPO**		**PRRX1**		**DHRS2**	**ABCC4**	**S100A2**	**SLC39A6**			
	**ACTN1**		**PRSS2**		**SOX4**	**LRIG1**	**BEAN**	**DNAJB4**			
	**ACTN4**		**PTHLH**		**DNAJB5**	**TMOD1**	**NMB**	**APLP2**			
	**MICALL2**		**PTX3**		**NA**	**SYNJ2**	**NAV1**	**PDLIM5**			
	**RAB8A**		**PVR**		**LOC728264**	**SYNJ2**	**NA**	**SAMD9**			
	**RAB8B**		**QSOX1**		**EPHA4**	**ITGAE**	**TREM1**	**DEGS1**			
	**RAPGEF6**		**RGS4**		**COL27A1**	**TFEB**	**PDK4**	**STC2**			
	**RAP1A**		**RHOB**		**SMAD7**	**NR4A3**	**MEF2A**	**MLLT10**			
	**ITGB1**		**SAT1**		**F2RL1**	**APOBEC3G**	**ST3GAL6**	**NMD3**			
	**AFDN**		**SCG2**		**NA**	**SYNJ2**	**NA**	**SLC29A1**			
	**TJP2**		**SDC1**		**NA**	**CDYL**	**CDK14**	**ATP6V1C1**			
	**RAPGEF2**		**SDC4**		**NA**	**ABCC4**	**DPYSL3**	**TLK1**			

	**RAP2C**		**SERPINE1**		**KDM6B**	**JUN**	**SPTBN1**	**PPAP2B**			
	**MARVELD2**		**SERPINE2**		**LOC100128178**	**SLC1A4**	**HPGD**	**FXR1**			
	**TUBA1B**		**SERPINH1**		**NA**	**FBXO11**	**CAMK2N1**	**TGFB2**			
	**TUBA4A**		**SFRP1**		**NA**	**APOBEC3G**	**PHLDA1**	**RANBP9**			
	**TUBA3C**		**SFRP4**		**RASL12**	**AQP3**	**RUNX2**	**PDLIM5**			
	**TUBA1A**		**SGCB**		**SLC35F2**	**LRIG1**	**SLIT3**	**CHMP1B**			
	**TUBA1C**		**SGCD**		**SETBP1**	**IL7R**	**HPGD**	**ATP6V1G1**			
	**TUBA8**		**SGCG**		**NA**	**ZNF365**	**USP46**	**MCL1**			
	**TUBA3E**		**SLC6A8**		**DOCK10**	**DCLRE1C**	**LRRC32**	**SPTBN1**			
	**TUBA3D**		**SLIT2**		**NA**	**IL9**	**ANGPT2**	**BCAT1**			
	**TUBAL3**		**SLIT3**		**C5orf13**	**RASGRP3**	**FADS3**	**NCOA3**			
			**SNAI2**		**DNAJC18**	**BMP1**	**ADAM9**	**CALU**			
			**SNTB1**		**DACT1**	**SLC1A4**	**CD151**	**SMARCA2**			
			**SPARC**		**WNT9A**	**RGS16**	**RGS16**	**MCFD2**			
			**SPOCK1**		**ETV6**	**ATP1B1**	**GPX3**	**TRIM23**			
			**SPP1**		**FGF18**	**IL7R**	**SLC44A2**	**NAB1**			
			**TAGLN**		**HBEGF**	**SLC1A4**	**GULP1**	**ZEB1**			
			**TFPI2**		**TNC**	**TIAM1**	**MID2**	**ADAM10**			
			**TGFB1**		**SDC1**	**CDYL**	**EBF1**	**MAP3K7**			
			**TGFBI**		**KIAA1755**	**ZNF238**	**ADAMTS2**	**TPP1**			
			**TGFBR3**		**COL27A1**	**MYH6**	**LYNX1**	**FZD6**			
			**TGM2**		**EDN1**	**LRIG1**	**COL22A1**	**THBS1**			
			**THBS1**		**HBEGF**	**RIMS3**	**NA**	**MMP14**			

			THBS 2		ITGB6	SLC1A4	BNC2	CALU			
			THY1		MURC	C18orf1	NA	KIDINS220			
			TIMP 1		PTGS2	GPR35	SDCCAG8	HMOX1			
			TIMP 3		EPHA4	DCLRE1C	ORAI2	SPSB1			
			TNC		PLEK2	CDYL	MMP19	BICD2			
			TNFA IP3		COL27A1	SYNJ2	THBS1	ZFHX3			
			TNFR SF11 B		NA	MB	PDE4DIP	OSBPL8			
			TNFR SF12 A		RHOU	ZNF238	DENND3	WSB1			
			TPM 1		NA		BHLHE40	APPL2			
			TPM 2		LOC201651		LAMC1	USO1			
			TPM 4		NA		ZEB1	DDA1			
			VCA M1		STEAP2		HPGD	PRKAR1A			
			VCA N		EDN1		DNASE2	IL6ST			
			VEGF A		SLC46A3		NA	PPAP2B			
			VEGF C		LMCD1		NA	IFNGR1			
			VIM		SNX30		NA	SNX3			
			WIPF 1		SOX4		NA	TAB2			
			WNT 5A		NA		ADAM8	YWHAZ			
					RYBP		FST	SLC6A8			
					NA		IL1RAP	APBB2			
					SOX4		FST	MAF			
					TMEM49		GABARAPL1	LEPR			
					NA		DLC1	MMP14			
					MGC16121		AZI2	PICALM			

					SORBS2		ABHD2	CCNG2			
					KDM6B		THBS1	SAR1A			
					PTGS2		SOCS6	RC3H2			
					LOC100128178		MRC2	CCL2			
					HIC1		NA	PDLIM7			
					NEDD9		UBXN2B	HPS5			
					ARHGEF40		NA	SLC7A11			
					FN1		IL1RAP	ICAM1			
					IFIH1		HTRA4	FHL1			
					GZMK		BNC2	IFI16			
					VEPH1		SPOCD1	FAM129A			
					EPHA4		ADARB1	FHL1			
					FOXP1		ANO4	VAMP3			
					PIK3CD		DFNB31	FAM198B			
					IL6		RGS1	LAMA4			
					YIPF5		SDS	F2R			
					SKIL		DCLK1	RGS4			
					RASD1		OBSL1	DNASE2			
					JARID2		DPP4	JAG1			
					NEDD9		DFNB31	IL32			
					ETV6		TMEM180	SRGN			
					IL11		C21orf7	MAP3K7			
					SETBP1		GNG11	NCK1			
					SNAI1		NA	FHL1			
					NA		CCL7	DKK1			
					NA		UPP1	ICAM1			
					NA		FZD4	BEX4			
					SOX6		FCGR1B	TES			

					SYNE1		DPH3	MYO6			
					STK38L		UBXN2B	ANTXR1			
					NKX3-1		NA	NUPR1			
					CDH6		NA	TUG1			
					NA		N4BP2L2	TCF4			
					PELI1		GRAMD1B	RASA3			
					PRDM1		GLI3	ZMYM6			
					PDPN		FGD6	SS18			
					WNT2		FBLN5	RAB23			
					LMO4		KLF10	CELF2			
					C4orf26		CLEC5A	PRKCI			
					OSGIN2		MAMLD1	B3GNT2			
					CACHD1		NA	KIAA1033			
					PRR5L		TCEAL3	ARF6			
					SOX6		SEMA4B	GOLGA2			
					TMEM2		ST3GAL6	ANKRD12			
					DDX10		IRS1	PARVA			
					MTSS1		BNC2	NID1			
					ARHGEF40		GPC1	CLIP1			
					CLDN14		WBP5	11-Sep			
					JHDM1D		MYOZ1	EID1			
					SLC19A2		NA	MET			
					PLCE1		FPR3	PTPN12			
					PRR9		PDE4DIP	SCARB2			
					MEGF9		NA	C3orf52			
					GOPC		PROC	PROSC			
					NA		FNDC3B	HSP90AA1			
					NA		FZD10	ITGA2			

					NA		MRO	HIPK3			
					MSC		ADM	AOX1			
					PPP1R14C		NA	TJP1			
					NA		NA	PRNP			
					PKNOX2		MSR1	MET			
					MSX2		NA	LAPTM5			
					SNCAIP		PLXDC2	ARL6IP5			
					SLC35F3		NA	MET			
					ETV6		MBD6	SS18			
					PRR5L		PDLIM7	NA			
					LOC727930		LDLRAD3	PICALM			
					HS3ST3B1		NA	SQLE			
					IL11		SLC22A4	SEC23A			
					MEOX1		SH2D3C	FKBP1A			
					STK38L		KCNK10	SOAT1			
					SNCAIP		FRY	TNFSF18			
					NA		PMEPA1	NPTX1			
					E2F7		MSR1	PPAP2B			
					AUTS2		RIN3	RAB3B			
					FUT4		MOAP1	BCL2L1			
					DLX2		NA	CREG1			
					ETV6		TMEM223	ITGB3			
					TBX3		PECAM1	RIOK3			
					TBX3		PDK4	PKN2			
							DIO3	ST6GALNAC4			
							NA	PDLIM5			
							NA	IQGAP1			
							TTC6	IL6ST			

							FST	TRIO			
							IL1RL1	TPR			
							DIO3OS	DAB2			
							CPE	ITGB4			
							FAM89A	GNS			
							CD300LF	PPP1R2			
							SLC29A1	LIG4			
							FMNL2	LYPLA1			
							MTUS1	PAK2			
							PDLIM7	CLDN1			
							BNC2	NA			
							GRB10	RNF146			
							NA	EIF5A			
							NA	FHL1			
							FAM110B	PTK2			
							LOC646903	NBN			
							CRTAC1	CPNE3			
							RGS1	RAB14			
							BNC2	PANX1			
							C5orf62	ARNTL			
							DOCK9	ST6GALNAC4			
							TLR7	TWSG1			
							AXL	NCOA3			
							NMBR	TSPAN3			
							SDCCAG8	IGF2BP3			
							ALOX5	PVR			
							DNM3	YY1			
							LHFPL2	PHTF2			

							ITGA2	APLP2			
							FGF18	CDH2			
							IFIT2	CDK17			
							TGFBR1	SLC6A15			
							NA	HIST1H2BE			
							BTC	TBL1XR1			
							TANC1	BCAP29			
							CDK5RAP2	CSNK1A1			
							FLRT2	HDGFRP3			
							NA	CBS			
							FAM27E3	NRP2			
							NA	CD58			
							NA	GLUD1			
							MGC24103	SLC16A1			
							TSPAN2	SPAG9			
							SLC31A2	PPFIBP1			
							SPRY1	CREBL2			
							NA	PSEN1			
							NA	RYK			
							NA	F2RL1			
							TNFRSF12A	MAX			
							SH2D3C	ARF4			
							NA	KIAA1109			
							RGL3	B4GALT1			
							FHL3	KIAA1033			
							LOC283033	ROCK1			
							LDB2	HSPG2			
							NA	TANK			

							ATP11B	RAC2			
							DAPK1	PROSC			
							NA	VAMP3			
							GAL3ST4	BCL2L1			
							TMEM163	PLEC			
							ABCD1	PXN			
							LTB4R	TM9SF1			
							SLC6A8	ECE1			
							NAV1	EID1			
							ALOX5AP	SCARB2			
							MASP1	TRIO			
							TMEM51	IL8			
							XRCC6BP1	B4GALT1			
							RNF17	NBN			
							LAIR1	SPTBN1			
							NA	DSTYK			
							NA	SCAMP1			
							NA	PLD1			
							ZNF618	LUC7L3			
							ADAM17	TPM1			
							GRB10	PLAA			
							DUSP1	ARFGEF1			
							DHX58	KDELR1			
							PHLDA1	CCPG1			
							NCEH1	LAPTM4B			
							SERPINE1	GLUD2			
							ADAMTS2	SERPINB9			
							SLC22A15	PTP4A1			

							NA	ATP2B1			
							NA	IL6ST			
							NCOR2	HSPA13			
							PMEPA1	PTP4A1			
							ZNF555	WDFY3			
							HOXC10	NA			
							PDLIM7	RIOK3			
							ARL9	MDFIC			
							TSKU	TMCO3			
							NA	IFI27			
							NA	POLG			
							SPATA12	RALB			
							CD109	MTUS1			
							NA	NA			
							C1orf204	NA			
							PHLDA1	SOS2			
							CHD3	OGT			
							IQCG	NA			
							LOC100128501	ITGA6			
							SLFN5	HIPK3			
							NA	CD58			
							CCDC40	GNB1			
							NA	IGF2BP3			
							S100A11	ATP6V1C1			
							MAPK8IP3	MCL1			
							TM7SF4	CBFB			
							AICDA	SPIN1			
							PLEKHA7	SOS2			

							**C1orf106**	**LMAN1**				
							**UNC5C**	**PPPDE1**				
							**MYOZ3**	**UBA6**				
							**ADCY2**	**ATP2B4**				
							**DNASE2**	**CDC73**				
							**NA**	**WNK1**				
							**KCNH3**	**ATP2B1**				
							**IL28RA**	**ZNF22**				
							**JAG1**	**IQGAP1**				
							**NA**	**LPP**				
							**MAP3K2**	**SCAMP1**				
							**COL8A2**	**FLI1**				
							**TPST1**	**NEDD4**				
							**B3GALNT1**	**PCDHGA3**				
							**FGF7**	**PPP1R12A**				
							**NA**	**IL6ST**				
							**DKK1**	**PON2**				
							**PRRG4**	**UBE2D3**				
							**NA**	**TTC3**				
							**PPARD**	**MEIS3P1**				
							**BTG1**	**LAPTM4B**				
							**GPR157**	**CSDA**				
							**AREG**	**TMOD3**				
							**FER1L4**	**PTPN11**				
							**UNC5B**	**SH3BGRL**				
							**LOC100240734**	**EIF3A**				
							**RP1L1**	**PHACTR2**				
							**DNM3**	**NA**				

							NUPR1	CRK			
							PHLDA1	UBE2J1			
							PKIA	RYBP			
							IQCH	DOCK9			
							WRNIP1	SDPR			
							MCOLN3	NCKAP1			
							NA	CLDN11			
							TTC7A	CD58			
							RAB23	NFAT5			
							NA	NA			
							PHF17	SHC1			
							EVX1	UBE2G1			
							FPR1	MAP4K5			
							AQP9	RPRD1A			
							JAG1	TGFB2			
							SIM1	PPFIA1			
							CACNA1G	SCAMP1			
							NA	EPAS1			
							NFKBIL2	NFATC2IP			
							NA	EPB41L5			
							FAM84B	FGF2			
							FAM7A3	KLHL7			
							PEX14	SGPP1			
							MBOAT7	HERC4			
							GUCA1A	MICAL2			
							NA	SP110			
							RUNX1	PHACTR2			
							PMEPA1	GSN			

							BEST1	CCNA1				
							DUSP1	SEC14L1				
							SPAM1	RAB5C				
							ADAMTS18	RAD21				
							HCFC1R1	PTGS2				
							PPOX	MAP3K2				
							STOX2	UTP3				
							SPATS2L	GLIPR1				
							TOP1P2	NAA35				
							ARL4A	NBR1				
							VSIG1	TAF9B				
							NA	NEDD9				
							NA	TOX4				
							P4HA2	FLRT2				
							NA	MBNL1				
							KCP	LOXL2				
							TRIM55	EPB41L3				
							LOC283050	NFIB				
							RHOB	TPP1				
							ABHD2	LIMS1				
							NA	PVR				
							DISP1	CALM1				
							MMP14	TMX1				
							NA	SIRPA				
							MMP7	DCTD				
							ABCG2	CHP				
							ABHD8	NF1				
							ARL5A	PAFAH1B1				

							**G0S2**	**PRKAR1A**			
							**CMTM1**	**ACBD3**			
							**FFAR1**	**TFDP1**			
							**MYH8**	**FEM1B**			
							**TES**	**HHLA3**			
							**NA**	**SYNM**			
							**SEMA6B**	**YIPF5**			
							**CES1**	**NFYA**			
							**NA**	**MGEA5**			
							**SLC10A2**	**PHACTR2**			
							**KLHL6**	**LYN**			
							**LAPTM5**	**KITLG**			
							**NA**	**CEACAM1**			
							**NA**	**SNAP23**			
							**CLDN12**	**TPP2**			
							**GPR26**	**NRAS**			
							**NA**	**ASF1A**			
							**PRRG4**	**XPO7**			
							**LOC151438**	**CDC5L**			
							**NA**	**RASA1**			
							**C1orf150**	**NA**			
							**PEX2**	**07-Mar**			
							**MAP3K2**	**LMAN1**			
							**NA**	**IL13RA1**			
							**NA**	**ITPR3**			
							**NA**	**GLG1**			
							**FAM113B**	**KPNA1**			
							**FABP6**	**NA**			

							NA	ATP13A3			
							NA	EXOC5			
							SNTB1	LIPG			
							LIMS1	NA			
							SNTB1	ABCF2			
							KLF15	IL1RL1			
							SOAT1	ASNS			
							MTUS1	HSP90B1			
							SH2D1B	ATG5			
							RNASEH2B	ELL2			
							MET	WAC			
							MAP3K2	ATP6AP2			
							SLC39A12	FAIM			
							NA	ABI1			
							KRT13	ADH5			
							RAB3B	PDE4B			
							MAN2A2	PWP1			
							CNBD1	ASPH			
							VASH1	DNAJC10			
							SEC22C	SELT			
							UBQLNL	02-Sep			
							SUCLG1	GLUL			
							JAG1	ENO1			
							ZFYVE19	PTPRF			
							RIF1	CAPN7			
							RHOBTB2	RBM9			
							UNC80	NA			
							ARMC8	C5orf28			

							CADM2	EIF4G1			
							VASH1	HSF1			
							C3	HSPH1			
							SLC8A1	NONO			
							ABCC3	IKBKB			
							DPY19L1	NEDD9			
							TSPAN5	TNFRSF10C			
							MCOLN3	TMEM41B			
							TMEM163	PTPRO			
							NA	GNB1			
							AAA1	KPNA4			
							ABHD2	SPTLC1			
							NA	KIAA1109			
							SLC26A10	NRCAM			
							NA	RAB5A			
							JAG1	TCF25			
							SMAD7	NUCB1			
							NA	RCN2			
							ABCC3	ZNF148			
							NA	LAMP1			
							NA	NA			
							HAMP	SMC3			
							NA	SOS1			
							NA	GTF2I			
							MERTK	RNFT1			
							TRIM62	IL1A			
							TP53I11	ITGA4			
							NA	SLC16A3			

							CLDND2	NA			
							SLC11A1	RBM25			
							SLC16A10	USP34			
							NA	ANXA4			
							AMPH	UBXN4			
							SLC6A8	ETS1			
							ZNF687	DIAPH1			
							NA	SYPL1			
							CNTNAP3B	UBXN4			
							TPM1	EXTL3			
							GPX7	CALR			
							FNDC3B	MGAT2			
							SLC44A2	CREB1			
							NCRNA00113	G3BP2			
							ABHD2	SLC4A7			
							SLIT3	KLHL9			
							MTHFD1L	ELF1			
							NOS3	HDGFRP3			
							LOC286437	YWHAB			
							SLC24A2	PPFIA1			
							RNF6	SMARCA2			
							SERINC2	CCDC6			
							PSME4	SELE			
							C8orf55	MFSD6			
							NA	SORBS3			
							NA	CYP51A1			
							NA	KITLG			
							MRO	CD44			

							NA	RAPGEF2			
							NA	PIGC			
							NA	TOX4			
							NA	EDEM3			
							TSPAN3	ATP6V1A			
							CADM1	ZFR			
							SLC16A3	USP10			
							NA	CPD			
							LOC154822	WTAP			
							TFAP2C	TPR			
							NA	GDE1			
							FPR3	DHX9			
							NA	UTRN			
							FAM40B	CDC27			
							IL3RA	GFRA1			
							SDCCAG8	TWF1			
							NA	STIP1			
							NA	PSAT1			
							STOX2	GMFB			
							IFIT3	TNFRSF10D			
							SH3GL2	RERE			
							NA	COMT			
							GLCCI1	NUP50			
							NA	CD164			
							KRTAP11-1	TGOLN2			
							NA	WIZ			
							C1orf91	ENC1			
							EPAS1	M6PR			

							OR10D3	S100P				
							ASIP	PGRMC1				
							TDRD9	RAD23A				
							NA	NUDT4				
							YPEL2	PAFAH1B1				
							MIA3	LIN7C				
							NA	GPD2				
							BTBD19	IL13RA1				
							GPR84	RABEP1				
							NA	VDAC1				
							PHC2	ANXA4				
							BNC2	MED20				
							MGST1	DMD				
							NA	EML4				
							NA	SPRY2				
							NA	SLC11A2				
							NA	TRIO				
							MFAP3L	UTRN				
							NA	SLC33A1				
							NR4A2	SMAD5				
							NA	NA				
							GLUL	BCLAF1				
							TLE3	NONO				
							TLE3	BUB3				
							NA	NUS1P3				
							MAPKAPK2	SENP3				
							DDA1	DUSP3				
							NA	ZWILCH				

							**TPD52L1**	**PSG6**			
							**NA**	**MAPK1**			
							**FOXD4**	**MANEA**			
							**GNB4**	**PPPDE1**			
							**LSR**	**SPAST**			
							**SMURF1**	**SNRNP27**			
							**C2orf40**	**RNF115**			
							**FKRP**	**PSPH**			
							**NA**	**TOR1AIP1**			
							**CLCN4**	**PTPN11**			
							**OSM**	**SMC3**			
							**SLC8A1**	**MED6**			
							**HECW1**	**MAX**			
							**NA**	**EPRS**			
							**DIRAS2**	**VPS41**			
							**CLVS1**	**USP46**			
							**MME**	**SMEK1**			
							**NA**	**YWHAE**			
							**COL22A1**	**FKBP1A**			
							**NA**	**SCYL2**			
							**ZNF549**	**SNAP23**			
							**01-Dec**	**CTH**			
							**FRY**	**HIPK2**			
							**SKIL**	**TOR1AIP1**			
							**SS18**	**ACLY**			
							**DGCR6L**	**TNFRSF10B**			
							**RWDD2A**	**PRKACB**			
							**MBD4**	**CD164**			

							MBD4	CREB1			
							NA	GBX2			
							RUNX1T1	MT1E			
							PXN	FBXW11			
							LOC283104	MT1X			
							ECE1	PRKCI			
							FZD7	CEACAM1			
							RNASE4	DDX18			
							PRIM2	G3BP2			
							BTBD11	TRRAP			
							TLX3	CLPTM1			
							NA	GRB2			
							NA	PAPOLA			
							ABHD2	PREPL			
							HP	FNDC3A			
							FAM40B	MT1M			
							MTHFD1L	NFYB			
							TLR7	NA			
							ZNF589	POT1			
							TRIO	PRPF4			
							PRPF40B	GGA3			
							SOX18	SNAPC3			
							DDHD1	SRSF2IP			
							CLPS	ABCB1			
							ERMP1	CFLAR			
							NA	KIAA0494			
							SLC16A8	C1orf144			
							CDKN1A	PTMS			

							AK7	CBFB			
							STX6	KPNA6			
							PHYHD1	C6orf106			
							PCBP3	RPE			
							OLFML3	MAGT1			
							NA	SLC26A2			
							ZBTB16	ABCC1			
							C9orf117	TPD52			
							TPD52L1	LRP8			
							SOX4	SYNCRIP			
							NA	PCBD1			
							PCDHGB8P	PVR			
							EYA2	MAPKAPK2			
							SNTB1	SLC1A4			
							TMTC1	SMARCC1			
							EN1	SLC7A1			
							NA	METAP2			
							NA	ANKFY1			
							MAP3K2	EPRS			
							ELL2	CLCN3			
							SLC45A3	SNX4			
							HRG	MBTPS1			
							DENND5B	CSNK1A1			
							MDFIC	ALG13			
							TGM2	GPR56			
							NA	FOXJ3			
							GPD2	MFNG			
							NA	TRIO			

							NA	AP1S1			
							MFGE8	DCTN4			
							NA	EDEM3			
							HBEGF	HSP90B1			
							AKR1C2	TSN			
							GLDN	SLC7A1			
							OCIAD2	TRA2B			
							LONRF3	RIPK1			
							ELL2	SERINC3			
							MAP9	GM2A			
							NA	TAGLN2			
							NA	SMG1			
							LOC283454	OSBP			
							NA	QPCT			
							PSME4	MAN1A1			
							NA	SH3GLB2			
							NTRK2	FUCA1			
							SLC6A12	APBA1			
							IL18RAP	GSPT1			
							PLEKHA5	MCM4			
							NA	H2AFY			
							NT5DC4	RANBP2			
							CPD	HADHA			
							NA	PTPRF			
							SLC8A1	TLR4			
							ANKRD29	DAPK3			
							PCMTD1	ELP3			
							FAM178B	NA			

							**ZFP36L1**	**SETD8**				
							**SLFN5**	**ARFIP1**				
							**SMURF2**	**ZFY**				
							**SIGLEC8**	**C19orf6**				
							**LOC284454**	**TGFBR2**				
							**LOC401097**	**TANK**				
							**C17orf91**	**BSG**				
							**NLRP12**	**NA**				
							**USP53**	**NETO2**				
							**AZI2**	**SERINC3**				
							**NA**	**ENG**				
							**C19orf59**	**MAPK14**				
							**NPR1**	**ADD1**				
							**IFIT3**	**FAS**				
							**NA**	**CD46**				
							**FMNL3**	**ARF3**				
							**NA**	**HMGA1**				
							**SLC11A1**	**AGFG1**				
							**NA**	**HNRNPC**				
							**SLC41A1**	**MYO9B**				
							**KCNA3**	**YAP1**				
							**CACNA2D1**	**DDX3X**				
							**ARID3B**	**PCK2**				
							**GAPT**	**PRPF40A**				
							**ALOX5**	**SEL1L**				
							**BLOC1S2**	**RAB6A**				
							**IGF2BP1**	**GLIPR1**				
							**NA**	**ACLY**				

							NA	NOLC1			
							CADM1	MAPRE1			
							NA	CDV3			
							PCNX	SKAP2			
							NA	ACTR2			
							SCD	ZC3H15			
							NA	NA			
							NA	MAPK1			
							CCDC157	ZMYND11			
							NA	ITPR2			
							BLMH	DAZAP2			
							NA	EDC3			
							SLC5A7	PCYOX1			
							MFI2	MAP2K2			
							NA	TFG			
							FKBP15	KCTD20			
							CPD	C19orf6			
							TRMT1	DOCK9			
							NA	MTMR1			
							FCGR3B	CAPRIN1			
							NA	SMARCC1			
							NA	PIP5K1A			
							TEX14	HNRNPH1			
							NA	BCLAF1			
							OK/SW-CL.36	C14orf101			
							ABCC3	TMED2			
							COX15	CD46			
							MEX3D	CLN5			

							MERTK	KIAA0494				
							LTA4H	SEL1L				
							NA	GRLF1				
							AKR1C1	MPZL1				
							CNIH4	ROD1				
							NPTXR	SH3BP4				
							NA	CFLAR				
							PPARD	SKAP2				
							TP53INP2	BCLAF1				
							C12orf59	ACTR2				
							KLHL29	AP2B1				
							LARGE	PRKDC				
							NA	SEC23IP				
							MMP2	RRN3P1				
							NA	NUMA1				
							NA	GDI2				
							NA	ME2				
							KCNC3	CYB5R4				
							NAPSB	DCLRE1C				
							ACADVL	NAA15				
							NA	NNT				
							C1orf126	TRIM27				
							NA	DLG1				
							NA	IGF2R				
							CYP19A1	GTF2F1				
							NA	TUBGCP2				
							TNNT2	ERC1				
							NRK	COPA				

							MSTN	NA			
							CYLD	DLAT			
							RGS8	DHX9			
							NA	SCP2			
							HDGFRP3	MAGOH			
							SLC16A10	SRP72			
							HS3ST5	TLE4			
							LOC255167	MAT2A			
							KCNIP2	UBE4B			
							IL17RB	FAM120A			
							ANG	SOCS2			
							FLJ13197	CANX			
							TPSB2	SSR1			
							NA	FAS			
							FURIN	AASDHPPT			
							ABCC3	NA			
							TGFBR1	API5			
							CLDN11	ROD1			
							NEK6	SLC30A1			
							SLC11A1	GGA2			
							CD58	VAC14			
							PYROXD1	KPNA3			
							ALK	TTC37			
							GOLT1A	SSB			
							CNKSR3	C1orf103			
							CHD9	KIAA0494			
							RHOH	ARF1			
							L3MBTL4	AP1S1			

							GLDN	06-Mar			
							NA	RELN			
							HAL	ARHGDIA			
							NA	USP46			
							S100A8	TNFRSF10B			
							NLRC4	PVR			
							PTGER3	C20orf30			
							NA	FAS			
							CYP19A1	PNO1			
							CLP1	ADD3			
							SLC35F4	CANX			
							IL27RA	PSME4			
							PARVA	MPZL1			
							CARD11	TTC3			
							NA	TFRC			
							MTMR11	PRPF4B			
							PDK4	RAB1A			
							FSTL5	RHOBTB3			
							NA	ZNF192			
							CSN1S1	CHD4			
							ANK1	ATP1B1			
							BCAS4	NA			
							CNIH4	EIF1AX			
							VASH1	DEDD			
							PPIH	MOBKL1B			
							NA	TP53			
							TP53I11	TMBIM6			
							NA	API5			

							**NA**	**DDX3X**			
							**SLC11A1**	**HPCAL1**			
							**DIXDC1**	**ANAPC5**			
							**ALOX5**	**STX16**			
							**VASH1**	**EIF4E**			
							**FOS**	**TGOLN2**			
							**CCDC40**	**TMED2**			
							**MIA2**	**NA**			
							**FOXQ1**	**MBTPS2**			
							**MNDA**	**COMMD10**			
							**NA**	**CLCN3**			
							**NA**	**ERLIN1**			
							**SLC1A2**	**SRSF10**			
							**CNIH4**	**PSG3**			
							**KIAA1274**	**FKBP15**			
							**TGM2**	**GLYR1**			
							**NA**	**STS**			
							**NA**	**TIA1**			
							**NA**	**ANP32A**			
							**COQ2**	**SYNJ1**			
							**WLS**	**KCTD5**			
							**MSRA**	**NFYC**			
							**NR4A3**	**LARP4B**			
							**APOC2**	**KDM6A**			
							**GSN**	**ARHGDIA**			
							**TFAP2B**	**POLR2E**			
							**CCNA1**	**WDR1**			
							**LRRC1**	**CCT2**			

							PRDM14	STX3				
							NA	AMD1				
							SMAD6	PDE8A				
							NA	U2AF2				
							NA	BRCC3				
							NA	SERBP1				
							NA	KIF5B				
							ST8SIA2	HSPD1				
							MAP3K2	POLDIP3				
							PHGDH	PNO1				
							NA	YKT6				
							TMEM111	UBE3B				
							STX6	SEC63				
							BEST1	G3BP1				
							PSEN1	SLC1A4				
							NA	ADD3				
							HIPK2	ILF3				
							MSR1	CYB5B				
							ARPP21	ROCK2				
							SBNO1	PTPN11				
							SP100	MOBKL1B				
							NEDD9	PTBP1				
							PKD2L1	RHOBTB3				
							FBP1	KIAA0776				
							CCDC40	ITGA4				
							BEST1	AGA				
							TTC12	GATC				
							FAM71F2	GPR137				

							TOPORS	GFPT1			
							STX6	STAM2			
							B3GNT5	DLG1			
							GPR25	ACTR2			
							NA	CDYL			
							NA	SMPD1			
							EWSR1	MARCKS			
							ADC	UBE2G2			
							FBXO9	EPB41L2			
							NA	AKIRIN1			
							DYX1C1	PSG6			
							CELF6	PREPL			
							TMEM38B	MCM3AP			
							NA	GART			
							TMED5	ESF1			
							NA	NA			
							DENND5B	CHD4			
							ZNF527	CLINT1			
							SPTBN1	DLG1			
							NA	SRPR			
							MEX3C	SEL1L3			
							NA	SLC35A2			
							LRRC27	SDHC			
							GCHFR	MMP1			
							PGLYRP2	AGPS			
							CPD	PLXNA2			
							TRIO	HNRNPR			
							LMX1A	SLC1A4			

							NA	GRAP			
							NA	SRRT			
							KCP	KIAA0182			
							GPR34	C6orf62			
							CADM4	ORC5L			
							ADAM8	NUDT21			
							AZI2	LSS			
							RIT2	CTSS			
							UTY	PDCD4			
							NA	TBX1			
							NA	RNF14			
							NA	DIMT1L			
							ARFGEF2	PLEKHB2			
							CDK5RAP2	SRSF1			
							NUP62CL	CSTF2T			
							PDCD1	HS2ST1			
							PTPDC1	CSNK2A1			
							PWRN2	GM2A			
							NA	TOP1			
							NA	PTPRB			
							PTPRZ1	LSM12			
							CDK3	USP1			
							RBP4	ZFPL1			
							NA	TSPAN12			
							NA	SLC35A3			
							PLAC1L	DLAT			
							C12orf49	UBE2K			
							LOC339807	CCND2			

							DYNC1H1	DSCR3				
							LRRC6	GCLM				
							NA	PAPSS2				
							CCDC40	SEMA3C				
							EFCAB5	SRPK1				
							SLC46A2	SEL1L3				
							NA	CALM1				
							NCR3	GOT1				
							TCEB3B	MOBKL1B				
							SDCCAG8	ACOT7				
							TUBE1	CSNK2A1				
							SKIL	DCAF7				
							FCRL1	IDH3A				
							NA	GALNT2				
							GPR37	PRPF6				
							NA	CCND2				
							NA	YBX1				
							RALA	C6orf62				
							SYTL1	UBE2N				
							NA	PPIF				
							NKTR	HSPA9				
							CES4	LARP4				
							CAMP	CYP20A1				
							DDX52	SLC35D1				
							IL17RB	EPHB2				
							EPB41L5	TFAM				
							FOXG1					
							NA					

							GOLGA8IP				
							FLJ33996				
							NA				
							GGT7				
							NA				
							NA				
							ECE1				
							SLC35D2				
							TCTN2				
							NA				
							MYO6				
							SLC44A5				
							ATP13A3				
							LIPG				
							DENR				
							ZNF540				
							TPTE2P1				
							NA				
							POU3F1				
							B3GNT5				
							OSM				
							BOLL				
							NA				
							A2ML1				
							S100A8				
							SH2D4A				
							KLHL6				
							LOC100287525				

							**SERPINB11**				
							**TBC1D10C**				
							**IL18BP**				
							**SDC1**				
							**NA**				
							**GPR110**				
							**NA**				
							**TPSAB1**				
							**SSH2**				
							**NA**				
							**C14orf109**				
							**NA**				
							**ELL2**				
							**COL8A2**				
							**DNAL1**				
							**TMEM38B**				
							**NDP**				
							**FGFRL1**				
							**PDE4DIP**				
							**XPO1**				
							**NA**				
							**MKI67IP**				
							**SLC7A1**				
							**NA**				
							**ALDH1A1**				
							**NA**				
							**PDXK**				
							**RDH12**				

							**DNMT3B**				
							**CYP2W1**				
							**SYT2**				
							**GLUL**				
							**NA**				
							**ZNF540**				
							**CLIP3**				
							**C9orf9**				
							**NA**				
							**NA**				
							**NA**				
							**DDX28**				
							**NSFL1C**				
							**STOX2**				
							**SPDEF**				
							**AGAP3**				
							**TPSAB1**				
							**NA**				
							**CCDC123**				
							**NA**				
							**ORAI2**				
							**KLF12**				
							**NA**				
							**SPINT1**				
							**NA**				
							**MIR155HG**				
							**NA**				
							**NA**				

							**KLHL29**				
							**DDX3X**				
							**ALK**				
							**LOC158696**				
							**ID3**				
							**LOC728431**				
							**NA**				
							**B3GNT7**				
							**MAB21L1**				
							**TGIF1**				
							**NA**				
							**NA**				
							**NA**				
							**CACNA1G**				
							**NA**				
							**FAM82A2**				
							**LOC100133131**				
							**RNFT1**				
							**LCK**				
							**IL1A**				
							**SLC16A3**				
							**NA**				
							**NA**				
							**STX6**				
							**MXD1**				
							**REPS2**				
							**C11orf75**				
							**MLPH**				

							**CCDC63**				
							**ENO3**				
							**RABGAP1L**				
							**SORL1**				
							**PHACTR1**				
							**ERBB3**				
							**NA**				
							**C11orf63**				
							**LOC731157**				
							**CCDC114**				
							**CHERP**				
							**SKI**				
							**ROCK1**				
							**NA**				
							**CPEB3**				
							**NA**				
							**NA**				
							**AZI2**				
							**TYRO3**				
							**CRX**				
							**FARP1**				
							**NA**				
							**NA**				
							**ESR2**				
							**ISM1**				
							**NA**				
							**FNDC3B**				
							**GLUL**				

							**UBE2E1**				
							**SMAD6**				
							**NA**				
							**BLID**				
							**WNT4**				
							**PTPN22**				
							**NA**				
							**NA**				
							**LOC285423**				
							**NA**				
							**LRPAP1**				
							**TNS1**				
							**NA**				
							**NA**				
							**RALGPS2**				
							**NA**				
							**SEMA7A**				
							**ADAMTS19**				
							**CPD**				
							**C17orf66**				
							**NA**				
							**SNAI3**				
							**DSTN**				
							**STOM**				
							**KCNIP4**				
							**CHD9**				
							**TACC2**				
							**SLC25A30**				

							**CXorf31**				
							**TP63**				
							**PDE4DIP**				
							**SLC5A12**				
							**NA**				
							**RRAD**				
							**NA**				
							**HAPLN1**				
							**SSH2**				
							**LTBR**				
							**NA**				
							**NA**				
							**ARL5A**				
							**SYT14**				
							**NA**				
							**MBD4**				
							**TNFRSF10D**				
							**03-Mar**				
							**PTPDC1**				
							**TPSAB1**				
							**CCDC68**				
							**NA**				
							**NA**				
							**NA**				
							**AK1**				
							**CALHM3**				
							**MAP4K2**				
							**MID2**				

							**SSH2**				
							**NA**				
							**NA**				
							**MAP1LC3A**				
							**IL1R2**				
							**KIAA0317**				
							**NA**				
							**NA**				
							**SCGN**				
							**CNTNAP3**				
							**ABCC3**				
							**NA**				
							**NA**				
							**AGPAT2**				
							**GNRHR**				
							**NA**				
							**AGRP**				
							**GPD2**				
							**TTC7A**				
							**HES1**				
							**MTMR11**				
							**DMD**				
							**MTMR3**				
							**EML4**				
							**DNASE1**				
							**PNKD**				
							**NA**				
							**ACER3**				

							**NPAS1**				
							**PSEN2**				
							**HIPK2**				
							**MAPK8**				
							**NA**				
							**LOC553137**				
							**UTRN**				
							**NA**				
							**BUB1**				
							**NA**				
							**TMEM38B**				
							**NA**				
							**DCUN1D3**				
							**NA**				
							**C8orf8**				
							**CDH1**				
							**NA**				
							**NA**				
							**IGKC**				
							**EDNRB**				
							**NA**				
							**NA**				
							**CDS1**				
							**DNASE1**				
							**GATA4**				
							**SLC44A5**				
							**WIPI1**				
							**PHTF1**				

							**FTCD**				
							**ZNF79**				
							**SH3RF3**				
							**C21orf67**				
							**FLJ30375**				
							**NA**				
							**ZFP41**				
							**NA**				
							**GLRX**				
							**NA**				
							**C10orf95**				
							**NA**				
							**ID2B**				
							**NA**				
							**NA**				
							**LOC541473**				
							**LAIR1**				
							**C6orf195**				
							**FNDC5**				
							**NA**				
							**NA**				
							**NA**				
							**WDR52**				
							**SPINK5**				
							**NA**				
							**LOC642587**				
							**NA**				
							**NA**				

							**RGS12**				
							**CNIH4**				
							**TGM5**				
							**KCP**				
							**NA**				
							**NA**				
							**CYGB**				
							**LONRF3**				
							**SYT5**				
							**SMAD6**				
							**NA**				
							**WLS**				
							**LOC389634**				
							**NA**				
							**LOC121952**				
							**DEFB124**				
							**NA**				
							**TNS1**				
							**CYB5A**				
							**NA**				
							**NA**				
							**MYO5BP2**				
							**NLRP12**				
							**P2RY11**				
							**RNF183**				
							**TLE3**				
							**ABHD5**				
							**C15orf62**				

							**TRIM2**				
							**LOC643837**				
							**PRSS21**				
							**SLC6A4**				
							**TBC1D16**				
							**ICK**				
							**AQP3**				
							**ABCD1**				
							**OR51E1**				
							**MINK1**				
							**ZNF589**				
							**NA**				
							**ZNF800**				
							**OLIG2**				
							**KLHL6**				
							**UNC13C**				
							**RNF166**				
							**LOC729178**				
							**NA**				
							**ST18**				
							**PDPN**				
							**NA**				
							**CDKN1C**				
							**PPP2R2C**				
							**GAD2**				
							**NA**				
							**KCNK13**				
							**C14orf56**				

							**NA**				
							**KLK2**				
							**LRRC43**				
							**TRAIP**				
							**NA**				
							**NA**				
							**TSPAN13**				
							**MASP1**				
							**FMNL3**				
							**CACNA1E**				
							**FAM71B**				
							**BPY2**				
							**DBF4B**				
							**SYTL3**				
							**NA**				
							**CYP2F1**				
							**OR1J4**				
							**GBP6**				
							**PLCG1**				
							**NA**				
							**NA**				
							**NA**				
							**FBXO9**				
							**LIPJ**				
							**SCD**				
							**NA**				
							**NA**				
							**TPD52**				

							**NA**				
							**KLHL11**				
							**C9orf44**				
							**LRRC37BP1**				
							**NA**				
							**IL1R2**				
							**NA**				
							**MAPKAPK2**				
							**REPS2**				
							**TMEM207**				
							**TANC2**				
							**NA**				
							**GPR157**				
							**TFEB**				
							**TRIM36**				
							**NA**				
							**TPSAB1**				
							**SLCO2B1**				
							**PRR5L**				
							**PPM1H**				
							**PEX14**				
							**CLDN23**				
							**TRPM3**				
							**ERMP1**				
							**AMBRA1**				
							**REPS2**				
							**IL17RB**				
							**SLC31A1**				

							NA				
							TRIO				
							CYP19A1				
							ASB15				
							NA				
							NA				
							NA				
							NA				
							NKG7				
							C20orf7				
							C12orf48				
							C18orf8				
							SNX7				
							LHX6				
							USP22				
							TLR3				
							NA				
							TASP1				
							NA				
							SNX10				
							SLC4A11				
							NA				
							MBD4				
							RAPGEF3				
							RNFT1				
							NA				
							ST3GAL6				
							TFCP2L1				

							**APOC4**				
							**NA**				
							**HOXB8**				
							**NEB**				
							**QPCT**				
							**SGCB**				
							**DUSP16**				
							**ICOSLG**				
							**NEK6**				
							**ACER3**				
							**CAPN5**				
							**EHF**				
							**NA**				
							**ENOPH1**				
							**NA**				
							**NA**				
							**SCD**				
							**DGAT2**				
							**TIAM2**				
							**INADL**				
							**N4BP2L1**				
							**LOC149773**				
							**TFR2**				
							**HN1**				
							**SLC23A3**				
							**LOC646576**				
							**CYP27A1**				
							**CYB5A**				

							**NA**				
							**KIAA1244**				
							**NA**				
							**HPS4**				
							**SKIL**				
							**PNPLA7**				
							**NEK6**				
							**CKLF**				
							**GPR125**				
							**NA**				
							**NA**				
							**EPB41L5**				
							**BUB1**				
							**PTH2R**				
							**NA**				
							**ESR1**				
							**PAQR5**				
							**ENG**				
							**NA**				
							**ABHD5**				
							**DNAJC6**				
							**AACSL**				
							**OR4D2**				
							**ATP2B2**				
							**PARP10**				
							**SLC24A1**				
							**LFNG**				
							**TFEB**				

							**NA**				
							**TFCP2L1**				
							**CYP4Z2P**				
							**ABCG1**				
							**NA**				
							**NA**				
							**EPB41**				
							**SLC6A15**				
							**PKD1L2**				
							**NA**				
							**NA**				
							**KLF14**				
							**NA**				
							**ARHGAP11A**				
							**LOC441461**				
							**ABHD5**				
							**NA**				
							**NA**				
							**RALA**				
							**RHEB**				
							**SRRM3**				
							**NA**				
							**SERHL2**				
							**POPDC3**				
							**APOL6**				
							**NA**				
							**CLDN6**				
							**NA**				

							**NA**				
							**CYB5A**				
							**SLC31A1**				
							**NA**				
							**TBC1D2**				
							**NA**				
							**NA**				
							**NA**				
							**GCG**				
							**NSUN7**				
							**NA**				
							**AZI2**				
							**C12orf37**				
							**CKLF**				
							**KIAA2026**				
							**NA**				
							**MGST1**				
							**FANCC**				
							**RSPO2**				
							**CCR7**				
							**SCCPDH**				
							**PSG7**				
							**C14orf142**				
							**IQCF3**				
							**CN5H6.4**				
							**LPAR3**				
							**MCOLN3**				
							**ST14**				

							NA				
							ST8SIA1				
							NA				
							SORL1				
							FAM65C				
							CXXC4				
							ASGR1				
							ZAN				
							C1orf64				
							CNTN5				
							OPALIN				
							RASGEF1B				
							NA				
							LOC100240726				
							HES1				
							NA				
							NA				
							SPATA2				
							SLC36A4				
							FBXL17				
							NA				
							NA				
							SVEP1				
							NA				
							ATP9A				
							PRAM1				
							SKIL				
							NA				

							OGFOD1				
							SDS				
							CEACAM21				
							CIT				
							TYRO3				
							BMX				
							EDNRB				
							TAGAP				
							C10orf35				
							TBC1D16				
							SH3BP4				
							NA				
							LOC339751				
							NA				
							SLCO2B1				
							IL7R				
							MAPKAP1				
							KRT7				
							SAMD14				
							SPAG1				
							PDPN				
							GPRIN3				
							TLE3				
							CHDH				
							NA				
							NAPSB				
							TGM2				
							NA				

							**DYX1C1**				
							**CARNS1**				
							**NA**				
							**DENND1B**				
							**FHAD1**				
							**MCOLN3**				
							**NA**				
							**ABCG1**				
							**WIPI1**				
							**BIN2**				
							**NA**				
							**LOC100293679**				
							**NA**				
							**KCNA2**				
							**ZNF81**				
							**TRA2A**				
							**NCRNA00257**				
							**TMEM144**				
							**MFI2**				
							**MS4A12**				
							**KL**				
							**ITGA9**				
							**NA**				
							**TNFSF13**				
							**KLHL6**				
							**TRIO**				
							**NA**				
							**GPCPD1**				

							NA				
							NCRNA00221				
							ACER3				
							NA				
							CADM1				
							NUDT22				
							CHGB				
							LOC440602				
							SDSL				
							ID2				
							NA				
							SLC31A1				
							CLDN23				
							NA				
							PARD3B				
							PTPN22				
							LOC728705				
							SLC9A5				
							NA				
							PTGDR				
							PDE4DIP				
							SARDH				
							GPCPD1				
							NA				
							CD1D				
							NA				
							GRAMD4				
							NA				

							**LTB**				
							**RGS6**				
							**NA**				
							**NA**				
							**ERN2**				
							**ST14**				
							**CNR1**				
							**CRNDE**				
							**HIVEP3**				
							**NA**				
							**NA**				
							**NA**				
							**TGM4**				
							**CKLF**				
							**NA**				
							**NA**				
							**ACER3**				
							**CHDH**				
							**ALDOB**				
							**TPMT**				
							**SLC27A2**				
							**NA**				
							**NA**				
							**PSME4**				
							**PTPRD**				
							**CASZ1**				
							**CAPN9**				
							**ZC3H12B**				

							**LOC646482**				
							**NA**				
							**MAP3K2**				
							**NA**				
							**SPIRE2**				
							**ZNF589**				
							**SCML4**				
							**NA**				
							**RNF213**				
							**PRSS8**				
							**STRBP**				
							**KLHL23**				
							**OR10H3**				
							**C14orf162**				
							**NA**				
							**NA**				
							**MRPS12**				
							**ITGB3**				
							**ITGB1BP1**				
							**WDHD1**				
							**C22orf45**				
							**FAM164A**				
							**NA**				
							**IL4**				
							**SYDE1**				
							**NA**				
							**NUSAP1**				
							**TAS2R4**				

							**ZNF451**				
							**NA**				
							**NA**				
							**C17orf99**				
							**NA**				
							**C18orf62**				
							**EDNRB**				
							**ACER3**				
							**NA**				
							**WLS**				
							**VDR**				
							**TRIM2**				
							**NA**				
							**PRR15**				
							**PGP**				
							**ATP6V0A2**				
							**DHDH**				
							**NA**				
							**NA**				
							**DGAT2**				
							**NA**				
							**TRAF3IP2**				
							**MAST3**				
							**NA**				
							**NA**				
							**ERLIN1**				
							**NA**				
							**NA**				

							**C6orf142**				
							**TNXB**				
							**NA**				
							**TRIM58**				
							**KIAA1543**				
							**SLC22A5**				
							**NA**				
							**LOC151121**				
							**LOC553103**				
							**KIF23**				
							**TNFSF14**				
							**PSME4**				
							**IL18R1**				
							**PDE4B**				
							**GKAP1**				
							**LOC285045**				
							**NA**				
							**IL7R**				
							**AKR1C3**				
							**VPS45**				
							**NA**				
							**VWF**				
							**HES1**				
							**GPRIN3**				
							**GH1**				
							**NA**				
							**NA**				
							**CCDC129**				

							**CEACAM21**				
							**STEAP3**				
							**NA**				
							**TSPAN17**				
							**PM20D2**				
							**PKHD1L1**				
							**NA**				
							**ZNF608**				
							**SOX1**				
							**HMGB3**				
							**MYOZ2**				
							**NA**				
							**FLJ37453**				
							**ATOH8**				
							**ERGIC1**				
							**SERPINA1**				
							**NA**				
							**ATP8B1**				
							**CDS1**				
							**KRT222**				
							**IL1B**				
							**NA**				
							**NA**				
							**FAM195A**				
							**NA**				
							**VRK3**				
							**MRE11A**				
							**DENND1B**				

							**C18orf16**				
							**GPCPD1**				
							**NA**				
							**CNTNAP2**				
							**FAM195A**				
							**NEK11**				
							**NA**				
							**NA**				
							**EDN3**				
							**HNF4A**				
							**NA**				
							**GLTSCR2**				
							**ARID5B**				
							**DAB2IP**				
							**STX6**				
							**TRIM50**				
							**PNPLA3**				
							**LOC389023**				
							**BCL2L2**				
							**DKFZp566F0947**				
							**NA**				
							**MGST1**				
							**VDR**				
							**MAOA**				
							**H2AFY2**				
							**HRK**				
							**NA**				
							**SYNPR**				

							BSND				
							HN1				
							GPD1				
							ALX4				
							CRMP1				
							NA				
							TBC1D16				
							NA				
							SLC25A37				
							WDHD1				
							NA				
							MGST1				
							PRY				
							PTK2B				
							LOC340017				
							CAMK4				
							MGST1				
							FANCI				
							C15orf48				
							NA				
							ATP9B				
							CHDH				
							ADAM3A				
							HOXA3				
							GNA14				
							MPP7				
							MBNL1				
							FLJ90680				

							NA				
							C11orf93				
							TFCP2L1				
							SLC34A1				
							TMEM185A				
							NA				
							C6orf182				
							KRT38				
							PAPLN				
							SEMA6A				
							HS6ST3				
							MAP1LC3A				
							C5orf56				
							BRSK1				
							IL1B				
							APCDD1				
							SLC35F1				
							NA				
							TMEM144				
							CCDC125				
							FDX1				
							RFFL				
							NA				
							SLC25A35				
							ANP32C				
							SERPINA1				
							LOC100131733				
							APOA2				

							ID1				
							SV2B				
							GALNT2				
							LOC652993				
							NA				
							ZNF552				
							TRAC				
							TOP2A				
							ID2				
							LYPD6				
							NA				
							NA				
							EPB41L5				
							IQGAP3				
							NA				
							ZNF627				
							NA				
							GPD2				
							NA				
							CDH23				
							L3MBTL4				
							NA				
							FNBP1L				
							PDE1B				
							NA				
							NA				
							LOC645638				
							DUSP16				

							**VDR**				
							**NKD1**				
							**PPP1R9A**				
							**PSPH**				
							**GABRA1**				
							**C9orf68**				
							**STRBP**				
							**NA**				
							**GLTPD2**				
							**SAMD11**				
							**MAOA**				
							**SLC35D1**				
							**CEACAM7**				
							**FGFRL1**				
							**MAPK13**				
							**NA**				
							**GAS2L1**				
							**ASF1B**				
							**TCF21**				
							**APBB1IP**				
							**CXCL14**				
							**CCDC67**				
							**HES5**				
							**NUSAP1**				
							**BLM**				
							**NA**				
							**TMEM54**				
							**NA**				

							**STOX1**				
							**XYLT1**				
							**CELSR2**				
							**NA**				
							**CDK1**				
							**CXCR6**				
							**SEMA3C**				
							**NA**				
							**NA**				
							**ZNF124**				
							**MAPK13**				
							**NA**				
							**TIAL1**				
							**RREB1**				
							**TBC1D16**				
							**C12orf49**				
							**APOB48R**				
							**HSD11B2**				
							**ERGIC1**				
							**CENPA**				
							**LOC100289219**				
							**ERGIC1**				
							**NA**				
							**AQP11**				
							**GRAP2**				
							**NA**				
							**CDC25A**				
							**SMAD6**				

							FAM84A				
							TBC1D16				
							PRLR				
							BTNL8				
							SEMA3C				
							GALNT2				
							WNT5A				
							CEBPA				
							NCRNA00265				
							WNT5A				
							NA				
							ARHGEF19				
							GPD1				
							NA				
							SOX6				
							PTTG1				
							ACVR1B				
							SAE1				
							MAEA				
							PPIF				
							ALDH2				
							NA				
							AGMAT				
							FAM46C				
							WNT5A				
							PAPOLB				
							NA				
							NA				

							**ASRGL1**				

Table S15: Significant differential expression of miRNA between each RECIST response group comparison

Group comparison	# of significant miRNA	mirna list
**SD (N=5) vs ARES (N=4)**	14	***miR-9-5p***, miR-184, miR-1269a, ***miR-204-5p***, miR-504-5p, miR-7641, miR-150-5p, miR-23b-5p, miR-27b-5p, miR342-3p, ***miR-365a-5p***, ***miR6510-3p***, miR-455-5p, ***miR-934***
**SD (n=5) vs IRES (n=4)**	14	***miR-9-5p***, miR-9-3p, ***miR-204-5p***, miR-1269a, miR-499a-5p, miR-200b-5p, ***miR-365a-5p***, miR-128-3p, ***miR-6510-3p***, miR-4664-3p, miR-144-5p, miR-486-5p, ***miR-934***, miR-206
**IRES (N=4) vs ARES (n=4)**	1	miR-184
